# Wandering throughout South America: Taxonomic revision of Tradescantia subg. Austrotradescantia (D.R.Hunt) M.Pell. (Commelinaceae)

**DOI:** 10.3897/phytokeys.101.25057

**Published:** 2018-06-27

**Authors:** Marco O.O. Pellegrini

**Affiliations:** 1 Universidade de São Paulo, Departamento de Botânica, Rua do Matão 277, CEP 05508-900, São Paulo, SP, Brazil

**Keywords:** Commelinales, Invasive species, *Tradescantia fluminensis*, Tradescantieae, Tradescantiinae, spiderworts, wandering Jew

## Abstract

I present the first taxonomic revision for T. subg. Austrotradescantia, based on extensive field, cultivation and herbaria studies. I accept 13 species, three of them (i.e. *T. atlantica*, *T. hertweckii* and *T. tucumanensis*) being described as new in the present study. I provide an identification key to the species, distribution maps, descriptions, comments, conservation assessments and illustrations for all species. The troublesome weed *T. fluminensis* has its specific limits clarified and its native range is presented so it can serve as a basis to better understanding its ecological requirements and to help control it throughout its invasive range. Furthermore, I highlight that *T. mundula*, a commonly neglected species closely related to *T. fluminensis*, might also represent a troublesome weed. *Tradescantia mundula* has been widely introduced in cultivation under the name *T. albiflora* and seems to have also escaped from cultivation. However, due to the hitherto poorly understood specific limits of *T. fluminensis*, *T. mundula* has been treated as a mere cultivar of *T. fluminensis* *s.s.*

## Introduction

Commelinaceae is an economically important family, due to the ornamental value of many genera and by the great number of invasive species, especially in *Commelina* L. and *Tradescantia* L. *emend* M.Pell. (Hunt 2001; Burns 2008). *Tradescantia*, is the second largest genus in the family, with ca. 90 species (Pellegrini 2017). It is characterised by its actinomorphic flowers with six equal to subequal stamens, seeds with linear hilum and main florescences composed by a double-cincinni fused back to back, with each cincinni subtended by a frondose bract (Faden 1998; Panigo et al. 2011; Pellegrini 2017). Throughout the years, four infrageneric classifications were proposed for *Tradescantia*: Clarke (1881), Brückner (1930), Hunt (1975, 1980, 1986) and Pellegrini (2017). The current classification divides *Tradescantia* into five monophyletic subgenera (Hertweck and Pires 2014; Pellegrini 2017), further supported by micro- and macromorphological characters, anatomy, phytochemistry and cytology (Pellegrini 2017).

Tradescantia sect. Austrotradescantia was described by Hunt (1980), being composed of five species, based on the *T. fluminensis* Vell. species complex (*sensu* Woodson Jr. 1942) and as the only exclusively South American section of the genus, with its diversity centre in Southeastern Brazil (Hunt 1980). It was characterised by its prostrate stems, leaf-like to spathaceous cincinni bracts, free petals and stamens, dorsal embryotega and numerous and bimodal chromosomes (Jones and Jopling 1972; Hunt 1980). Other studies showed that the section also presented a rather peculiar phytochemical profile (Martínez and Martínez 1993) and stigmatic morphology (Owens and Kimmins 1981; Owens et al. 1984), which differentiated it from the remaining sections and species of the genus. Based on micro- and macromorphological characters, anatomy, phytochemistry and cytology characters and supported by the molecular evidence published by Hertweck and Pires (2014), Pellegrini (2017) elevated T. sect. Austrotradescantia to the subgeneric rank, also broadening and clarifying the group’s morphological circumscription. Hunt (1980) originally included five species in the section, later (i.e. Hunt 2001) reducing *T. blossfeldiana* Mildbr. to a synonym of *T. cerinthoides* Kunth. Pellegrini et al. (2015) reestablished *T. mundula* Kunth and typified *T. fluminensis* Vell. and *T. geniculata* Vell., while dealing with the *Tradescantia* names in *Flora fluminensis* (Vellozo 1829, 1831). Pellegrini et al. (2016) solved the application of *T. cymbispatha* C.B.Clarke and considered the latter and *T. umbraculifera* Hand.-Mazz. as members of this section. Pellegrini (2016) described *T. seubertiana* M.Pell. and proposed an informal group named the *T. crassula* group. Pellegrini et al. (2017) described *T. chrysophylla* M.Pell. as a new species, treated *T. schwirkowskiana* Funez et al. as a synonym of *T. crassula* Link & Otto and included *T. valida* G.Brückn. in the section, as being the only species in the *T. crassula* group to present spathaceous and supernumerary bracts. Two recent floristic studies have recognised *T. multibracteata* M.Ferrarese et al. (Büneker et al. 2017) and *T. serrana* Hassemer & Funez (in Hassemer et al. 2017) as new species for T. sect. Austrotradescantia. Nonetheless, in my new infrageneric classification for *Tradescantia* (i.e. Pellegrini 2017), I intentionally do not mention these names amongst the species accepted by me for T. subg. Austrotradescantia, as a way to imply that I consider these names to be synonymous with other species. In the overview for T. subg. Austrotradescantia, presented by me as part of the new infrageneric classification for *Tradescantia* (i.e. Pellegrini 2017), the subgenus is represented by ca. 15 species, out of which I mention: *T. cerinthoides*, *T. chrysophylla*, *T. crassula*, *T. cymbispatha*, *T. fluminensis*, *T. mundula*, *T. seubertiana*, *T. tenella* Kunth, *T. umbraculifera* and *T. valida*. Members of this subgenus are economically important due to the ornamental value of their leaves (sometimes conspicuously striped with an array of colours), their mat-forming habit and for being hardy plants, being cultivated worldwide for these reasons. Subsequently, *T. fluminensis* has escaped from cultivation and has become a major weed in countries such as Australia, Italy, Japan, New Zealand, Portugal, Russia, South Africa, USA etc. (Hunt 2001; Burns 2008; Fowler et al. 2013; GSID 2015; pers. observ.).

Despite possessing relatively few species, T. subg. Austrotradescantia has a complicated taxonomic history (Pellegrini et al. 2015, 2016, 2017), due to continuous and reticulate morphological characters and possible natural hybridisation between its species (Martínez 1984; Pellegrini 2017), which makes the terminology adopted by Woodson Jr. (1942) of “species complex” rather appropriate for this group (Pellegrini et al. 2013). Despite that, no taxonomic revision has yet been made for the subgenus and no identification key is available for its species. Furthermore, aside from the morphological phylogeny and new infrageneric classification provided by Pellegrini (2017), no detailed study exclusively dealing with the taxonomy and morphology of the species of T. subg. Austrotradescantia has ever been made. Thus, I present the first taxonomic revision for T. subg. Austrotradescantia, with the description of three new species, an identification key and synonyms, descriptions, comments, illustrations, distribution maps and conservation assessments for all accepted species.

## Methods

The description of the species, phenology and illustrations were based on over 3,000 herbarium specimens (ALCB, B, BA, BHCB, BHZB, BM, BOTU, BRIT, C, CAL, CEPEC, CESJ, CGE, CGMS, CNMT, COR, CORD, CVRD, EAC, ESA, F, FCAB, FCQ, FLOR, FUEL, FURB, GUA, HAMAB, HAS, HB, HBR, HDCF, HRB, HRCB, HSTM, HUCS, HUEFS, HUFSJ, HURB, IAC, ICN, INPA, IPA, JOI, K, L, MBM, MBML, MG, MO, MY, NY, P, PACA, PMSP, R, RB, RFA, RFFP, RUSU, SCP, SP, SPF, SPSF, U, UEC, UFRN, UPCB, US, VIC, W, WAG and WU; herbaria acronyms according to Thiers, continuously updated), spirit, fresh and cultivated material and field observations. Specimens of all species, except *T. hertweckii* M.Pell., *sp. nov.* and *T. tucumanensis* M.Pell., *sp. nov.*, were seen in the field and later kept in cultivation at the greenhouse of the Jardim Botânico do Rio de Janeiro, in order to better observe, photograph and analyse fresh flowers, fruits and seeds as well as other phenological data. Fresh specimens, field notes, photographs and specimens for cultivation were gathered during several field trips across the Brazilian Atlantic Forest, from the states of Sergipe to Rio Grande do Sul, between 2008 and 2017. Fertile specimens were deposited in RB and, whenever possible, duplicates were sent to US. Indumentum and shape terminology follows Radford et al. (1974); the inflorescence and general morphology terminology follows Weberling (1965, 1989) and Panigo et al. (2011); the fruit terminology follows Spjut (1994); the seed terminology follows Faden (1991); and general macromorphological terminology follows Pellegrini (2015, 2017). The conservation assessments followed the recommendations of IUCN Red List Categories and Criteria, Version 3.1 (IUCN 2001). GeoCAT (Bachman et al. 2011) was used for calculating the Extent of Occurrence (EOO) and the Area of Occurrence (AOO). The distribution of the species is based on herbaria materials, field data and literature. The classification of vegetation patterns follows IBGE (2012). The rainy season in the Seasonal Dry Forest corresponds to the interval between October and March, which also roughly corresponds to Spring and Summer in South America (Colombo and Joly 2010); while in Atlantic Rainforest, there is no obvious rainy season, with rain relatively evenly distributed throughout the year with a slight peak during Summer (Colombo and Joly 2010).

## Results

In the present study, I accept 13 species for T. subg. Austrotradescantia, increasing by three the total number of species accepted by Pellegrini (2017). *Tradescantia anagallidea* Seub. is here formally reduced to a synonym of *T. tenella*, while *T. decora* is shown to be the correct name for the species formerly known as *T. valida* and *T. multibracteata*. Finally, *T. serrana* is reduced to a synonym of *T. chrysophylla*. This study represents the final publication from my MSc thesis (i.e. Pellegrini 2015), which dealt with the evolution, systematics and nomenclature of *Tradescantia*, added to a taxonomic revision of T. subg. Austrotradescantia.

### Tradescantia subg. Austrotradescantia

Taxon classificationPlantaeORDOFAMILIA

(D.R.Hunt) M.Pell., PhytoKeys 89: 47. 2017.

Tradescantia sect. Austrotradescantia D.R.Hunt, Kew Bull. 35(2): 440. 1980.
Tropitria Raf., Fl. Tell. 3: 68. 1837. Type species. Tropitria crassula (Link & Otto) Raf. (≡ T. crassula Link & Otto)

#### Type species.

*Tradescantia fluminensis* Vell.

#### Description.

*Herbs* chamaephytes, base definite or indefinite, perennial, frequently succulent, terrestrial, rupicolous or epiphytes. *Roots* thin, fibrous. *Stems* prostrate with ascending apex or erect, herbaceous to succulent, rarely fibrous, little to densely branched, rooting at the basal nodes or at the distal ones when they touch the substrate. *Leaves* sessile to subpetiolate; distichously or spirally-alternate, evenly distributed along the stem, rarely congested in a rosette; sheaths closed; ptyxis involute or convolute; blades flat to falcate and/or complicate, base asymmetrical, midvein conspicuous, rarely inconspicuous, adaxially impressed, abaxially prominent, rounded, secondary veins conspicuous or inconspicuous. *Synflorescences* terminal or axillary in the distal portion of the stems, composed of a solitary main florescence, 1–4 per leaf axis. *Inflorescences (main florescences)* consisting of a pedunculate double-cincinni fused back to back, sometimes composed of 1–3(–5) cincinni; inflorescence bract hyaline, tubular, inconspicuous; peduncle bracts absent; supernumerary bracts rarely present; cincinni bracts leaf-like, rarely spathaceous, differing from the leaves mostly only in size, similar or unequal to each other, saccate or not, free from each other; cincinni sessile, contracted, opposite to subopposite; bracteoles inconspicuous, imbricate, linear-triangular to triangular, hyaline. *Flowers* bisexual, actinomorphic, flat (not forming a floral tube); pedicel gibbous at apex, upright at anthesis and pre-anthesis, deflexed at post-anthesis; sepals equal, free, chartaceous, ovate, dorsally keeled or not, apex acute; petals sessile, equal, free, elliptic to ovate to broadly ovate, flat or plicate, base cuneate to obtuse, margin glabrous, apex acute; stamens 6, arranged in two series, equal, filaments free from the petals, straight at anthesis and post-anthesis, white, rarely pink, basally densely bearded with moniliform hairs, hairs as long as the stamens, white, anthers basifixed, rimose, connective rhomboid, yellow, anther sacs ellipsoid, yellow, pollen yellow; ovary subglobose to globose, white, glabrous, locules 2-ovulate, style straight at anthesis and post-anthesis, white, obconical at base, conical at the apex, stigma punctate, pistil longer than or the same length as the stamens. *Capsules* subglobose to globose, light to medium brown when mature, glabrous, loculicidal, 3-valved, sometimes apiculate due to persistent style base. *Seeds* 1–2 per locule, ellipsoid to narrowly trigonal, ventrally flattened, cleft or not towards the embryotega, testa costate to rugose with ridges radiating from the embryotega; embryotega dorsal, relatively inconspicuous, without a prominent apicule, generally covered by a cream farina; hilum linear, on a weak ridge.

#### Habitat, distribution and ecology.

**Figure 1.**
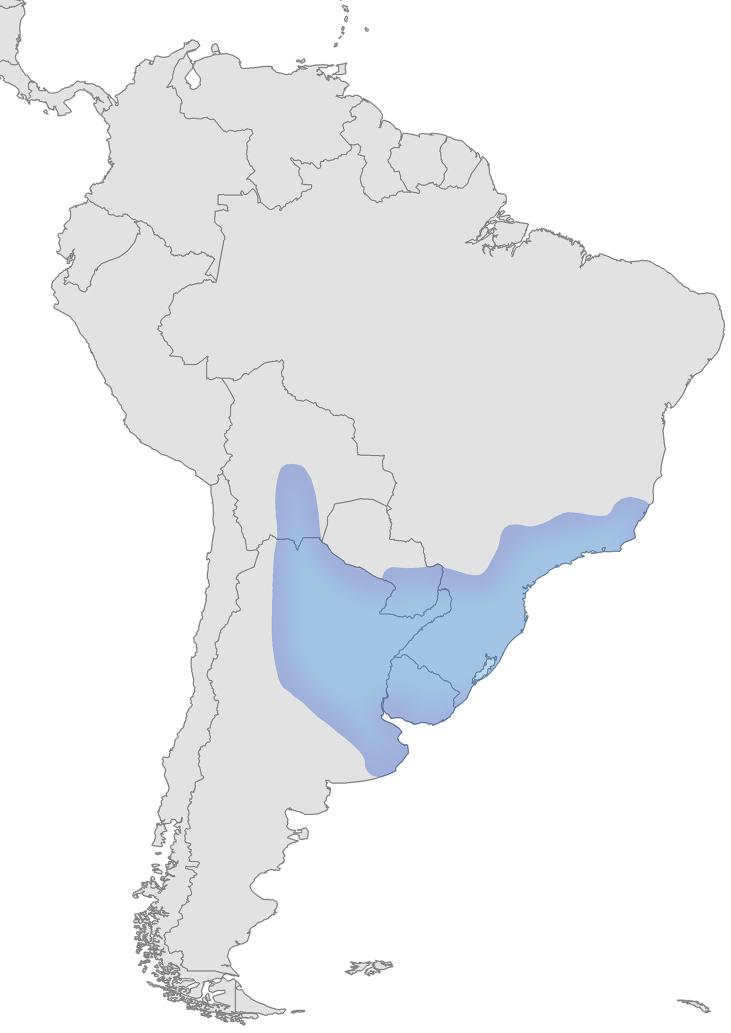
Distribution of Tradescantia subg. Austrotradescantia. Modified from Pellegrini (2017).

Tradescantia subg. Austrotradescantia is distributed from southern Bolivia, Paraguay, Argentina, Southeastern to Southern Brazil and Uruguay (Fig. 1). Its species can be found growing understorey in moist and shady forests, open fields, rocky outcrops and are especially common in disturbed areas (Pellegrini 2015, 2017; Pellegrini et al. 2016).

#### Conservation and invasiveness.

Tradescantia subg. Austrotradescantia as a whole seems to be in need of little conservationist attention. Most species possess wide native distribution, with only *T. atlantica*, *T. chrysophylla*, *T. hertweckii* and *T. seubertiana* possessing narrower distributions and thus meriting some conservationist attention. Out of the 13 species accepted in the present study, one (i.e. *T. fluminensis*) is already known to represent a troublesome weed worldwide, while the other species have never been considered invasive or to possess an invasive potential. Nonetheless, after careful examination of herbarium specimens collected outside the subgenus native distribution range, I came to the conclusion that several records of *T. fluminensis* as a weed actually represent misidentified specimens of *T. mundula* or, more rarely, specimens of *T. cymbispatha* and *T. crassula*. *Tradescantia cerinthoides* is also widely cultivated worldwide, especially its pink and lilac-flowered morphs. Despite my not having observed any unquestionable records that indicate that *T. cerinthoides* has escaped from cultivation, this species also possesses intense vegetative growth and thus a great potential to become an invasive species if not properly monitored. All invasive accessions done so far for *T. fluminensis* must urgently be redone in order to properly understand extension of this species’ invasion, discount records now known to represent other species from the subgenus and to appropriately access the threat of invasiveness of the other species of T. subg. Austrotradescantia which also possess records outside their native range.

#### Etymology.

The name of this subgenus means “*Tradescantia* from the South”, making reference to its exclusively South American distribution.

#### Comments.

Tradescantia subg. Austrotradescantia is a morphologically peculiar group in *Tradescantia*, being easily recognised by its generally distichously-alternate leaves, sepals ovate, generally all dorsally keeled; filaments basally densely bearded with long moniliform hairs; style obconic at base and conic at apex, stigma punctate with type D papillae; seeds with costate testa and relatively inconspicuous embryotega; small bimodal and numerous chromosomes (*n* = 10–numerous); and a unique chemical profile (Pellegrini 2017). Two morphological groups are accepted for the subgenus, both supported by recent phylogenetic studies (Pellegrini 2017). The *T. fluminensis* group is composed of generally more delicate plants, with prostrate stems with ascending apex, indefinite bases, subpetiolate leaves, cincinni bracts saccate at base and white petals. Nonetheless, this group also includes the *T. tenella* complex, which possesses erect stems, definite base, flowers that range from white to pink, seeds with rugose testa and hilum shorter than half the length of the seeds. The species in the *T. fluminensis* group occur almost exclusively in Tropical and Subtropical Rainforests but are also commonly found growing as weedy plants throughout their distribution range (Pellegrini 2017). The *T. crassula* group is composed of succulent plants, with erect stems, complicate leaves, cincinni bracts not saccate at base, petals ranging from white to pink to lilac and seeds cleft towards the embryotega. These species are intimately related to the two southern domains of South America, characterised by open and/or drier vegetation formations: the Chaco (which is part of the Dry Diagonal) and the Pampa (which is mostly represented by grasslands). The species from the *T. crassula* group are morphologically very similar due to many overlapping morphological characters; with indumentum type and distribution in the sepals being the most useful character for separating its species.

#### Recommendations for field collectors.

As widely known, Commelinaceae is a group where flowers are generally poorly preserved in herbarium specimens, making it especially difficult to work with (Faden 1991). In T. subg. Austrotradescantia, taxonomy relies greatly on indumentum characters, with its type, distribution and colouration being especially important. Moreover, the indumentum in the pedicels and sepals seems to be constant within the same species and variable between different species. Thus, I recommend that field workers pay special attention to the plant’s indumentum, collect young and mature branches in order to correctly characterise the species (since features like the presence of a subpetiole and shape of the leaf-blades might vary during development), record the colouration of vegetative and reproductive organs and, whenever possible, attach photographs to the herbarium sheets. Images are of great aid and can help identify even the most incomplete and damaged specimens. Furthermore, whenever possible, spirit collections and live specimens for cultivation are welcome, since they enable the proper study of the delicate flowers of these plants and live specimens might help us understand their morphological variation and plasticity.

#### Roots, stems and growth forms.

As stated by Pellegrini (2017), most *Tradescantia* species are perennial herbs, all of them lacking rhizomes. In T. subg. Austrotradescantia, the roots are always thin and fibrous and never tuberised as in many species of the other four subgenera. In the mat-forming species, roots are produced throughout the stems, whenever they touch the substrate. In the species with erect stems, roots are restricted to the basal-most nodes of the plants. The stems can vary in posture, from prostrate (generally with ascending apex) to erect, while the branching pattern ranges from unbranched to little branched at the base or branched to densely branched in the upper half. The leaf-opposed line of uniseriate hairs is generally observable in most species, except *T. seubertiana* which is completely glabrous. Nonetheless, it is not constant in any of the remaining species, being either present or absent, depending on the specimen or even on the maturation and/or position of the stem (i.e. younger shoots tend to produce this leaf-opposed line of uniseriate hairs, which is generally lost with age). Finally, in species densely covered by indumentum, such as *T. cerinthoides*, *T. chrysophylla*, *T. cymbispatha* and *T. mundula*, the leaf-opposed line of uniseriate hairs is generally absent or, if present, is very hard to differentiate from the dense surrounding indumentum.

#### Indumentum.

**Figure 2.**
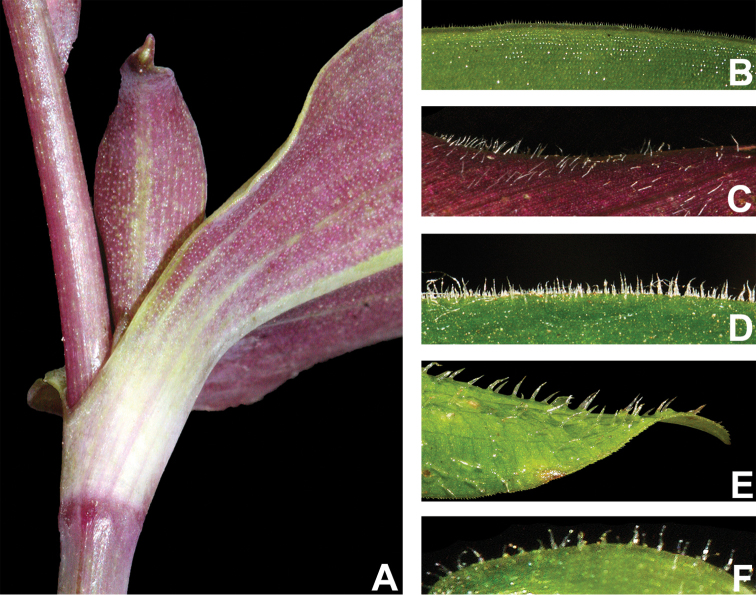
Indumentum morphology in Tradescantia subg. Austrotradescantia. **A** completely glabrous stems and leaves in *T. seubertiana* **B** glabrous leaf-blade of *T. crassula*, showing the ciliate margin **C** abaxial side of a leaf-blade of *T. cerinthoides*, showing the sparsely hispid indumentum **D** adaxial side of a leaf-blade of *T. cerinthoides*, showing the hispid indumentum **E** adaxial side of a leaf-blade of *T. tenella*, showing the hirsute indumentum and ciliate margin **F** sepal of *T. tenella*, showing the glandular-pubescent indumentum. Photos by M.O.O. Pellegrini.

The indumentum in T. subg. Austrotradescantia is consistently composed of uniseriate hairs, ranging from eglandular to glandular (Fig. 2C–F). Special attention should be also given to the prickle-hairs that are generally 2-celled and present at the margins of the leaf-sheaths and blades (Fig. 2B, E), but consistently absent only in *T. seubertiana* (Fig. 2A). Glandular hairs can be found in the vegetative organs of *T. cerinthoides* but are consistently found in the pedicels of most species and the sepals of many (Fig. 2F). As aforementioned, indumentum morphology is key for species delimitation in T. subg. Austrotradescantia, being easily observed most of the time. Nonetheless, some species are especially susceptible to the excessive heat of some plant driers, which can lead to an artificial loss of hairs. Two good examples are *T. cymbispatha* and *T. mundula*, where the first has been observed to sometimes lose its characteristic dense strigose indumentum in some excessively dried specimens from Argentina and Southern Brazil, while the second presents delicate sepal hairs that commonly fall during the drying process. However, due to the almost unique combination of morphological characters (e.g. sessile and succulent leaves, with generally elliptic blades and sepals lacking dorsal keels and evenly velutine), *T. cymbispatha* can be easily identified even when the vegetative hairs are almost completely lost during the excessive drying process. In *T. mundula*, this partial loss of sepal hairs can lead to confusion in the identification of dried specimens, making it even more similar to *T. fluminensis* *s.s.*

#### Leaves.

**Figure 3.**
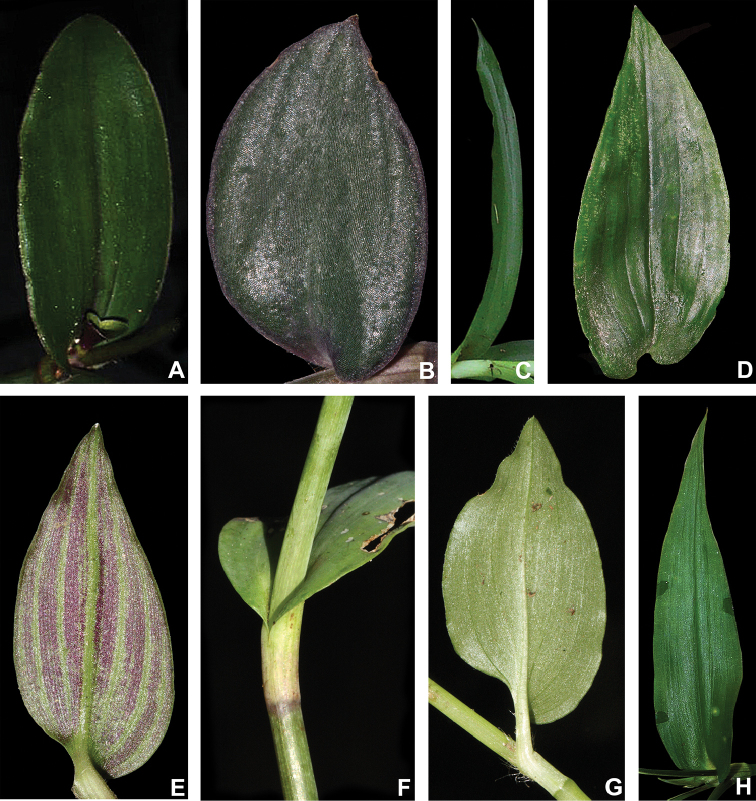
Leaf morphology in Tradescantia subg. Austrotradescantia. **A** elliptic blade of *T. cerinthoides*, showing the dark green colouration and obtuse apex **B** broadly ovate blade of *T. cymbispatha*, showing the bluish-green colouration, vinaceous margins, the densely strigose indumentum and cordate base **C** linear blade of *T. decora*, showing the conduplicate and falcate posture and truncate base **D** one of the uppermost leaves from *T. fluminensis*, showing the dark green colouration, inconspicuous subpetiole, impressed secondary veins and cordate base **E** blade of *T. mundula*, showing the short subpetiole, impressed secondary veins and round base **F** detail of the amplexicaulous leaf-blade base of *T. seubertiana* **G** blade of *T. tenella*, showing the distinctively subpetiolate leaf and strongly asymmetric base **H** blade of *T. umbraculifera*, showing the amplexicaulous base and caudate apex. All photos by M.O.O. Pellegrini, except for **C** by H.M. Bünecker.

The leaves in T. subg. Austrotradescantia can range from distichously- to spirally-alternate, sometimes in the same species (e.g. most species belonging to *T. crassula* group), from sessile to distinctively subpetiolate (Fig. 3), but always presenting an asymmetric base (Fig. 3). The blades tend to decrease in overall size towards the apex of the stem, while subpetioles decrease in length. Alternatively, the basal-most leaves tend to possess wider bases when compared to the leaves towards the apex of the stem. Subpetioles are restricted to some species in the *T. fluminensis* group, with sessile leaves being plesiomorphic in Commelinaceae (Pellegrini 2017). The apex of the leaf-blades is generally acute but can also range from acute to caudate or obtuse (Fig. 3). The base of the leaf-blades can range from cuneate to obtuse to cordate or from truncate to amplexicaulous (Fig. 3). Finally, the ptyxis can either be convolute or involute, with convolute leaves being restricted to the *T. crassula* group and involute leaves restricted to the *T. fluminensis* group.

#### Variegation.

**Figure 4.**
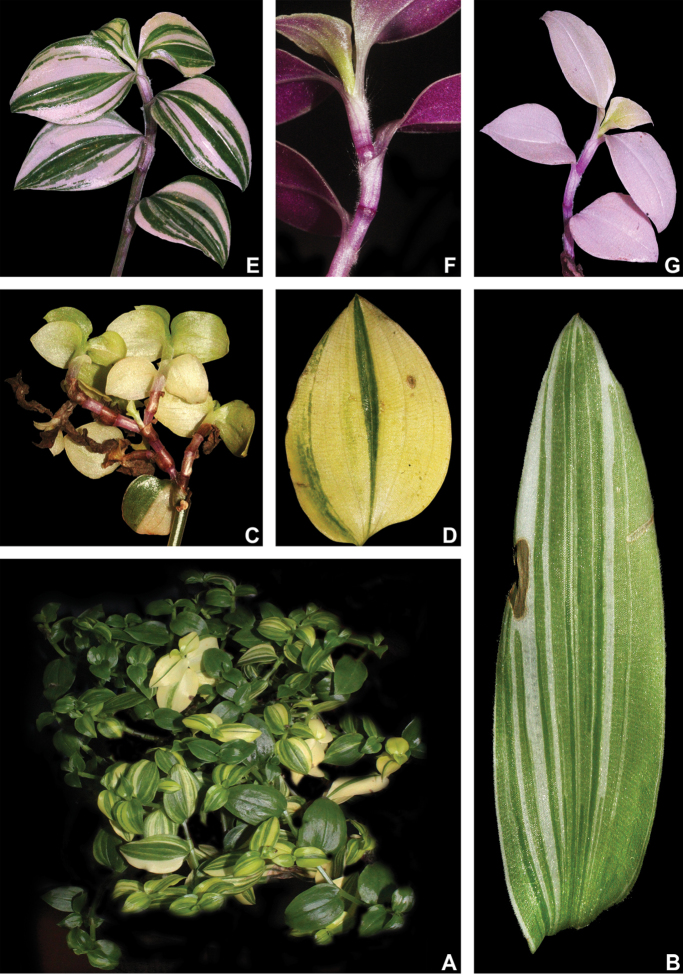
Leaf variegation in Tradescantia subg. Austrotradescantia cultivars **A** habit of *T. fluminensis*, showing different degrees of yellow variegation in the same plant **B** sick leaf blade of *T. decora*, showing the white variegation and a necrotic spot **C–D** *T. fluminensis* with yellow variegation **C** sick branch, showing atrophied and almost completely lacking chlorophyll **D** sick leaf blade, showing small portions of chlorophyllate tissue and necrotic spots. **E–H**, *T. mundula* with pink to vinaceous variegation **E** habit, showing different variegation patterns and degrees in the same plant **F** healthy branch, showing leaves with stripes in shades of pink and lilac **G** sick branch, showing the almost complete lack of chlorophyll and the great amount anthocyanin on the abaxial side of the blades **H** sick branch, showing the almost complete lack of chlorophyll and the pink hue produced by the anthocyanin from the abaxial side of the blades. Photos by M.O.O. Pellegrini.

Striped leaves have long been observed, described and utilised for species delimitation in the family. In some species (e.g. *T. soconuscana* Matuda and *T. zebrina* Heynh. *ex* Bosse and their relatives), it is a marking feature that greatly aids their recognition (Pellegrini 2017). According to my anatomical observations and ongoing studies on the matter, the silver stripes in Commelinaceae seem to consist of aerenchymatous tissue that might help understorey plants increase the amount of light they are able to absorb, by directing light through reflection to the inside of the chlorophyllate parenchyma (unpublished data). Nonetheless, this feature has been observed not to be constant in most taxa of the family, being environmentally controlled in at least *Buforrestia* C.B.Clarke, *Dichorisandra* J.C.Mikan, *Floscopa* Lour., *Plowmanianthus* Faden & C.R.Hardy and *Siderasis* Raf. emend. M.Pell. & Faden (pers. observ.). In *Tradescantia*, this feature seems to be phylogenetically related (at least to some degree), since it is only known to occur in T. subg. Campelia (Pellegrini 2017). In T. subg. Austrotradescantia, a myriad of cultivars of several species are known for their variegated leaves (e.g. *T. cerinthoides*, *T. decora*, *T. fluminensis* and *T. mundula*; Fig. 4), but also found in members of different subgenera [e.g. *T. spathacea* Sw., *T. zanonia* (L.) Sw. and *T. zebrina* – T. subg. Campelia –, *T. pallida* (Rose) D.R.Hunt and *T. sillamontana* Matuda – T. subg. Setcreasea –, but it is unknown to me to occur in T. subg. Mandonia and T. subg. Tradescantia; pers. observ.]. However, this variegation does not seem to be homologous to the silver stripes commonly observed in other members of the family. These stripes do not seem to be produced by the concentration of aerenchymatous tissue, but actually seem to be caused by the loss of pigmentation in the leaves. They actually look similar to the symptoms caused by some strains of the tulip breaking virus (family Potyviridae) in the perianth of Liliales (McKay and Warner 1933; McWhorter 1938) and more precisely to symptoms caused the commelina yellow mottle virus (*Badnavirus* spp., family Caulimoviridae), being generally malefic to the infected plants (Hull 2007; Valverde et al. 2012). These white stripes (sometimes also yellow or pink to vinaceous, due to the presence of secondary pigments; Fig. 4) are not commonly observed in natural populations, being almost exclusively recorded in cultivated plants (pers. observ.). During cultivation in the greenhouses of the Jardim Botânico do Rio de Janeiro, some specimens not originally striped, were observed to acquire such features. The appearance of white to yellow stripes in the leaves occurred shortly after a great aphid and mealybug infestation struck the live collection. Specimens from different genera, such as *Commelina* and *Floscopa* also acquired such stripes. After some months, the affected specimens either withered and died or survived and lost the stripes (pers. observ.). This pattern is coherent with the one observed in the transmission and spread of viruses from families Potyviridae, Caulimoviridae and other plant infecting families that cause mosaic and breaking patterns in plants (McKay and Warner 1933; McWhorter 1938; Andret-Link and Fuchs 2005; Hull 2007; Valverde et al. 2012). Thus, in the present study, striped specimens of T. subg. Austrotradescantia are disregarded, being considered merely as sick plants or artificially selected morphotypes of no taxonomic relevance.

#### Inflorescences.

**Figure 5.**
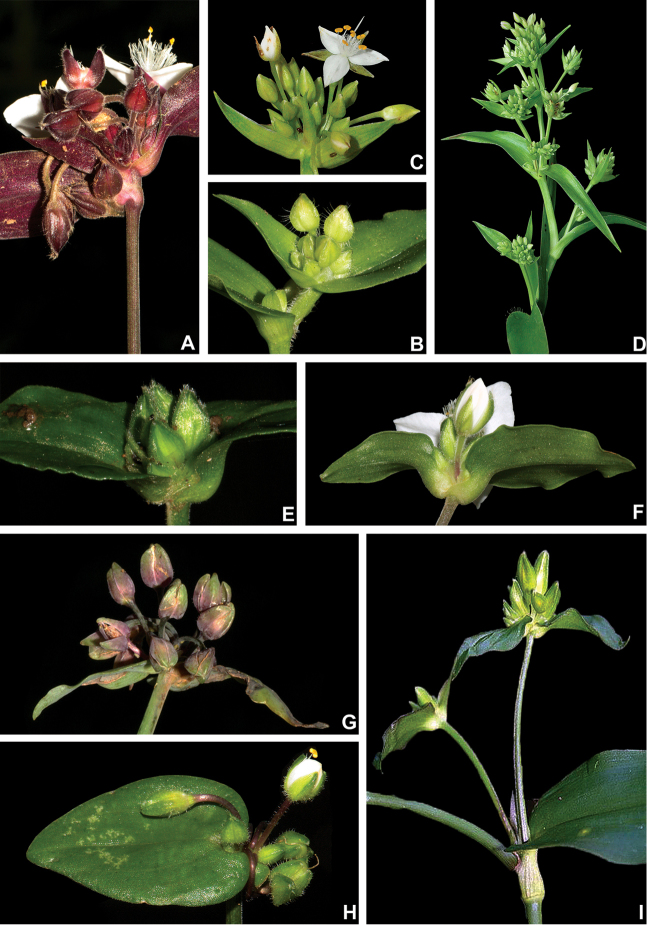
Inflorescence architecture in Tradescantia subg. Austrotradescantia **A** malformed main florescence of *T. cymbispatha*, showing the subopposite cincinni **B** detail of the synflorescence of *T. crassula*, showing an axillary inflorescence composed of solitary cincinnus and a terminal with the typical double-cincinni with non-saccate cincinni bracts **C–D** inflorescence in *T. decora* **C** inflorescence composed of 4-cincinni, showing the spathaceous and supernumerary bracts **D** synflorescence, showing main florescences and coflorescences ranging from regular double-cincinni to 3–5-cincinni **E** front view of the main florescence of *T. fluminensis*, showing the saccate cincinni bracts **F** dorsal view of the main florescence of *T. mundula*, showing the saccate cincinni bracts **G** main florescence of *T. seubertiana*, showing the unequal and non-saccate cincinni bracts **H** main florescence of *T. tenella*, showing the strongly unequal cincinni bracts **I** inflorescence of *T. umbraculifera*, showing the main florescence and a coflorescence emerging from the same leaf axil. All photos by M.O.O. Pellegrini, except **C–D** by H.M. Bünecker.

The inflorescence architecture in T. subg. Austrotradescantia follows the double-cincinni pattern, as described by Panigo et al. (2011) and indicated by Pellegrini (2017) as characteristic to *Callisia* *s.l.*, *Tradescantia* and *Tripogandra* *s.l.* The cincinni bracts are always frondose, being leaf-like in most species but spathaceous in *T. decora* and *T. umbraculifera* (Fig. 5C, D, I). The base of the cincinni bracts can be saccate or not, with saccate bracts being synapomorphic to the *T. fluminensis* group (Pellegrini 2017). Supernumerary bracts are rare in T. subg. Austrotradescantia, being exclusively recorded for *T. decora*. The cincinni are always sessile, contracted and fused back to back. Due to great reduction in the main florescence, the cincinni seem opposite in most specimens. Nonetheless, in some specimens, a malformation in the inflorescence can cause the internodes between the cincinni to elongate, thus producing subopposite cincinni (Fig. 5A; Pellegrini 2017). Most inflorescences are composed of two cincinni, as the double-cincinni architecture would suggest. Nonetheless, some exceptions are recorded, with *T. crassula* producing perfect double-cincinni, but also commonly producing axillary inflorescences composed of a solitary cincinnus (Fig. 5B; Pellegrini 2017). Furthermore, *T. decora* is the only species in T. subg. Austrotradescantia to regularly present main florescences with more than two cincinni, generally producing main florescences with 2–3(–5) cincinni (Fig. 5C, D; Pellegrini 2017).

#### Flowers.

**Figure 6.**
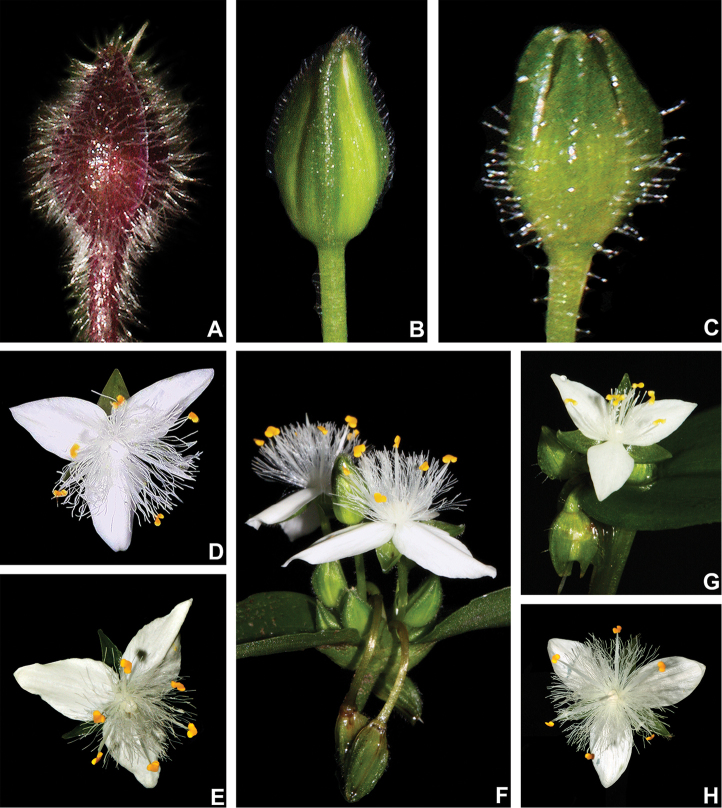
Floral morphology in Tradescantia subg. Austrotradescantia. **A–C** floral buds **A** floral bud of *T. cerinthoides*, showing the vinaceous coloration, densely hispid indumentum and absence of dorsal keels in the sepals **B** floral bud of *T. fluminensis*, showing the medium green colouration and pilose eglandular hairs restricted to dorsal keels **C** floral bud of *T. tenella*, showing the green colouration, glandular-pubescent indumentum and the presence of dorsal keels in the sepals **D–H** flowers **D** front view of a flower of *T. atlantica*, showing the plicate petals **E** front view of a flower of *T. fluminensis*, showing the plicate petals **F** side view of two flowers of *T. fluminensis*, showing the plicate petals and the deflexed flowers at post-anthesis **G** front view of a flower of *T. tenella*, showing the flat petals **H** front view of a flower of *T. umbraculifera*, showing the flat and elliptic petals. Photos by M.O.O. Pellegrini.

The flowers in T. subg. Austrotradescantia are always flat (i.e. not forming a floral tube), with flowers being held in an upright position at pre-anthesis and anthesis and later acquiring a deflexed position at post-anthesis and fruiting (Fig. 6F; Pellegrini 2017). As with the other species of *Tradescantia*, the species of T. subg. Austrotradescantia possess scentless flowers (Pellegrini 2017) and, like all species of Commelinaceae, completely lack nectaries of all kinds (Faden 1992). A marking floral conservatism can be easily observed in T. subg. Austrotradescantia, with gross floral morphology presenting little taxonomic relevance for species delimitation in the subgenus (Pellegrini 2017). Some taxonomic relevance can be given to the shape of the floral buds (that can be of great help in differentiating closely related taxa) and floral diameter (which can also help differentiate some species).

#### Sepals.

Aside from the pubescence, little variation is observed in the sepals of T. subg. Austrotradescantia. The sepals are always equal, free, chartaceous, ovate, with acute apex. They can range from medium to dark green or from purple to vinaceous to dark vinaceous and can be dorsally keeled or not (Fig. 6A–C; Pellegrini 2017). Measures can be of some help, but they do commonly overlap between closely related species.

#### Petals.

The deliquescent petals are also quite homogeneous in T. subg. Austrotradescantia, being always sessile (i.e. without a claw, like some species of T. subg. Campelia, T. subg. Mandonia and T. subg. Setcreasea and all species of T. subg. Tradescantia; Pellegrini 2017), equal, free, elliptic to ovate to broadly ovate, with cuneate to obtuse base, glabrous margin and acute apex. Little colour variation is observed, especially when compared with T. subg. Campelia and T. subg. Tradescantia. All species possess predominantly white petals, with the exception of *T. seubertiana*, which always possesses pink petals. Nonetheless, specimens also presenting petals in different hues of pink or lilac can sometimes be observed in *T. cerinthoides*, *T. cymbispatha*, *T. decora*, *T. tenella* and *T. tucumanensis*. Finally, petals in T. subg. Austrotradescantia can either be flat or plicate. The plicate petals are caused by a fold along the petals’ midvein and are exclusively found in *T. atlantica* M.Pell. *sp. nov.* and *T. fluminensis* *s.s.* (Fig. 6D–F).

#### Androecium.

As shown by Pellegrini (2017), all species of T. subg. Austrotradescantia possess filaments densely bearded at the base with moniliform hairs, these hairs being as long as the stamens or at least the filaments; anthers basifixed, with expanded, rhomboid and yellow connectives, divergent and elliptic anther sacs and yellow pollen grains *in vivo* (Fig. 6F). This pattern is exclusive to T. subg. Austrotradescantia (Pellegrini 2017), but of almost no relevance in differentiating the species within the subgenus.

#### Gynoecium.

The gynoecium in T. subg. Austrotradescantia presents a highly conservative morphology, being always white and glabrous, the ovary ranging from subglobose to globose, the locules 2-ovulate, ovules anatropous with axial placentation, the style being always straight at anthesis and post-anthesis, obconical at base, conical at the apex and culminating in a reduced and punctate stigma (Pellegrini 2017). Finally, the pistil, as a whole, can either be much longer than or approximately the same length as the stamens.

#### Capsules and seeds.

**Figure 7.**
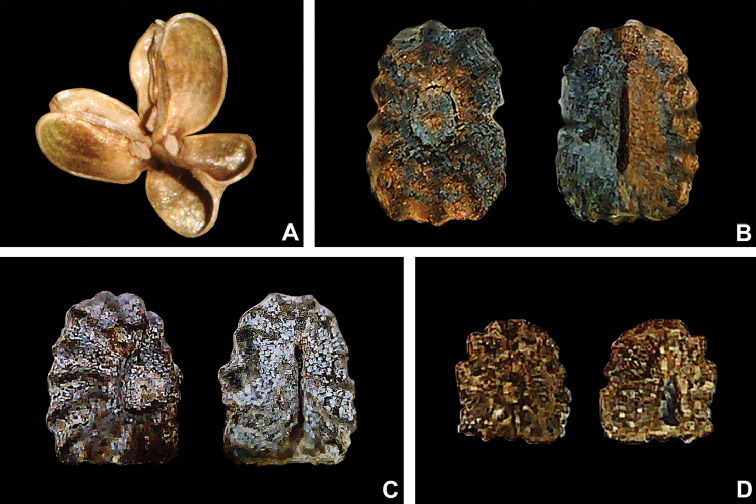
Fruit and seed morphology in Tradescantia subg. Austrotradescantia. **A** mature and dehisced capsule of *T. cerinthoides* **B** dorsal and ventral views of the seed of *T. crassula*, showing the costate testa cleft towards the embryotega and the hilum longer than ½ the length of the seed **C** dorsal and ventral views of the seed of *T. hertweckii*, showing the costate testa not cleft towards the embryotega and the hilum equal to ½ the length of the seed **D** dorsal and ventral views of the seed of *T. atlantica*, showing the rugose testa not cleft towards the embryotega and hilum shorter than ½ the length of the seed. Photos by M.O.O. Pellegrini.

Fruit morphology is extremely conservative in *Tradescantia*, being characterised by light to medium brown, thin-walled, loculicidal capsules, subglobose to globose in shape, externally glabrous and smooth and sometimes apiculate due to the persistent base of the style. The capsules are always 3-valved (Fig. 7A), with the only known exception in the genus being *T. orchidophylla* Rose & Hemsl., which seems to exclusively present a 2-locular gynoecium and, consequently, producing 2-valved capsules. Each locule can produce up to two seeds, which will directly influence their shape. The seeds range from ellipsoid to narrowly trigonal, commonly with a more truncate side if two seeds are produced in the same locule, ventrally flattened, cleft or not towards the embryotega (with cleft seeds being synapomorphic to the *T. crassula* group; Fig. 7B) and the testa can be either costate (Fig. 7B, C) or rugose, with ridges or pits radiating from the embryotega (i.e. rugose testa being exclusive to the *T. tenella* species complex; Fig. 7D). The embryotega is dorsal and relatively inconspicuous, without a prominent apicule, being generally covered by a cream farinae (Fig. 7B–D). The hilum is linear, located on a mild ridge and can vary in length depending on the species group: (1) longer than ½ the length the seed in the *T. crassula* group (Fig. 7B); (2) ca. equal to ½ the length of the seed in most species of the *T. fluminensis* group (Fig. 7C); and (3) shorter than ½ the length of the seed in the *T. tenella* species complex (Fig. 7D).

### Key to the species of T. subg. Austrotradescantia

**Table:** 

1	Stems erect, rarely prostrate with ascending apex; leaves with convolute ptyxis, blades succulent, falcate to complicate; cincinni bracts not saccate at base; seeds cleft towards the embryotega	**2**
–	Stems prostrate with ascending apex, less commonly erect; leaves with involute ptyxis, blades membranous to chartaceous, flat; cincinni bracts saccate at base; seeds not cleft towards the embryotega	**5**
2	Leaf-blades pubescent on both sides or only abaxially, rarely glabrous on both sides; pedicels and sepals densely velutine to hispid, sometimes also with a mixture of glandular and eglandular hairs	***T. cerinthoides* Kunth** (Fig. 10)
–	Leaf-blades glabrous on both sides; pedicels glabrous, rarely sparsely glandular-pubescent, sepals glabrous or with hairs restricted to the keel or apex	**3**
3	Leaf-blades with margins setose at base or until the middle with long hyaline hairs; supernumerary bracts present, cincinni bracts spathaceous, cincinni 2–3(–5); sepals not dorsally keeled	***T. decora* W.Bull** (Fig. 18)
–	Leaf-blades with margins glabrous or ciliolate to ciliate; supernumerary bracts absent, cincinni bracts leaf-like, cincinni 1–2; sepals dorsally keeled	**4**
4	Leaf-sheaths margins glabrous, base of the blades cordate to slightly amplexicaulous to obtuse; cincinni bracts unequal; floral buds ellipsoid; sepals glabrous; petals light pink to pink	***T. seubertiana* M.Pell.** (Fig. 26)
–	Leaf-sheaths margins ciliolate to ciliate, base of the blades obtuse to truncate; cincinni bracts equal; floral buds broadly ovoid; sepals sparsely setose along the keel; petals white	***T. crassula* Link & Otto** (Fig. 14)
5	Base definite; stems erect, rarely prostrate with ascending apex, succulent to fibrous; testa rugose, hilum shorter than ½ the length of the seed	**6**
–	Base indefinite; stems prostrate with ascending apex, herbaceous; testa costate, hilum longer than or equal to ½ the length of the seed	**8**
6	Stems fibrous; leaves sessile, blades velutine to hispid; sepals with indumentum restricted to the keel; petals always white, plicate	***T. atlantica* M.Pell.** (Fig. 8)
–	Stems succulent; basal leaves subpetiolate, blades hirsute to sparsely hirsute; sepals evenly covered with indumentum, rarely glabrous; petals white to pink, flat	**7**
7	Leaf-blades with ciliolate margins; floral buds ovoid, flowers 0.4–1.0 cm diam., sepals evenly to sparsely glandular-pubescent, rarely glabrous	***T. tenella* Kunth** (Fig. 28)
–	Leaf-blades with ciliate margins; floral buds ellipsoid, flowers 1.2–1.6 cm diam., sepals with a mixture of glandular and eglandular hairs, but exclusively hispid along the keel	***T. tucumanensis* M.Pell.** (Fig. 30)
8	Leaf-blades with truncate to amplexicaulous to round base, apex acuminate to caudate; 1–4 double-cincinni per leaf axis, cincinni bracts spathaceous, rarely leaf-like; pedicels white at anthesis and pre-anthesis; pistil longer than the stamens	***T. umbraculifera* Hand.-Mazz.** (Fig. 32.)
–	Leaf-blades with cuneate to obtuse to cordate base, apex acute, rarely acuminate; always 1 double-cincinni per leaf axis, cincinni bracts leaf-like; pedicels green to vinaceous at anthesis and pre-anthesis; pistil the same length as the stamens	**9**
9	Leaves sessile, blades evenly covered by indumentum on both sides, secondary veins adaxially inconspicuous; floral buds broadly ovoid, sepals not keeled	**10**
–	Leaves subpetiolate, rarely sessile (if sessile, blades with flat margins), blades glabrous or indumentum not evenly distributed on both sides, secondary veins adaxially impressed; floral buds ovoid, sepals keeled	**11**
10	Leaf-sheaths strigose, margins sparsely setose to setose, hairs hyaline to light brown, blades strigose, hairs hyaline, adaxially dark to medium bluish-green, margins glabrous; cincinni bracts similar to each other; pedicel and sepals velutine, petals white, rarely pink to lilac	***T. cymbispatha* C.B.Clarke** (Fig. 16)
–	Leaf-sheaths velutine to hispid, margins densely velutine to hispid, hairs golden, blades velutine to hispid, hairs golden to light brown, adaxially dark to medium green, margins ciliolate; cincinni bracts unequal or strongly unequal to each other; pedicels and sepals glandular-pubescent or with a mixture of glandular and eglandular hairs, petals always white	***T. chrysophylla* M.Pell.** (Fig. 12)
11	Stems strigose; leaf-sheaths strigose, blades chartaceous, abaxially vinaceous, margins slightly revolute; pedicels velutine, sometimes with some odd glandular hairs, sepals evenly velutine (hairs sometimes deciduous in dried specimens)	***T. mundula* Kunth** (Fig. 24)
–	Stems glabrous; leaf-sheaths glabrous, blades membranous to slightly fleshy, abaxially light to medium green, margins flat; pedicels glabrous to distally sparsely glandular-pubescent, sepals with hairs restricted to the keels	**12**
12	Leaves subpetiolate, blades glabrous, margins evenly ciliolate; cincinni bracts equal to each other; sepals pilose along the keels, petals plicate; hilum equal to ½ the length of the seed	***T. fluminensis* Vell.** (Fig. 20)
–	Leaf sessile, blades hispid, margins ciliate, base densely setose; cincinni bracts unequal or strongly unequal to each other; sepals setose along the keels, petals flat; hilum longer than ½ the length of the seed	***T. hertweckii* M.Pell.** (Fig. 22)

### 

#### 1. Tradescantia atlantica

Taxon classificationPlantaeORDOFAMILIA

M.Pell. sp. nov.

urn:lsid:ipni.org:names:77185915-1

Figs 8 , 9

##### Diagnosis.

Similar to *T. tenella* due to its definite base, erect and densely branched stems, involute ptyxis, leaf-blades with conspicuous secondary veins, saccate and strongly unequal cincinni bracts, keeled sepals, pistil the same length as the stamens, seeds with rugose testa and hilum shorter than ½ the length of the seed. It can be differentiated by its fibrous stems, sessile leaves with velutine to hispid, light brown to hyaline indumentum, broadly ovoid floral buds, sepals with a mixture of glandular and eglandular hairs restricted to the keel and petals always white and plicate.

##### Type.

BRAZIL. Rio de Janeiro: Nova Friburgo, Reserva Ecológica de Macaé de Cima, fl., fr., 26 Jan 2012, M.O.O. Pellegrini et al. 207 (holotype: RB barcode RB01025675!).

##### Description.

*Herbs* ca. 10–35 cm tall, with a definite base, terrestrial. *Stems* erect, fibrous, branched to densely branched; internodes 1.8–6.1 cm long at base, distally shorter, dark green to vinaceous, glabrous, except for a leaf-opposed longitudinal line of short, uniseriate, light brown to hyaline hairs. *Leaves* distichously-alternate, sessile; ptyxis involute; sheaths 4.1–7.6 mm long, light green to pink with dark green to purple striations, glabrous, margin setose, hairs light brown to hyaline; blades 3.3–10.2 × 0.9–3.4 cm, elliptic to ovate, flat, membranous to chartaceous, velutine to hispid on both sides, rarely hairs restricted to the midvein, hairs light brown to hyaline, adaxially dark green, abaxially green, sometimes with vinaceous blotches, turning dark brown to olive-green on both sides when dry, base obtuse to rounded, margin ciliolate, slightly revolute, apex acute to acuminate; midvein conspicuous, adaxially impressed, secondary veins conspicuous, adaxially impressed, abaxially prominent, becoming more evident on both sides when dry. *Synflorescences* terminal or axillar in the distal portion of the stems, composed of a solitary main florescence, 1 per leaf axis. *Inflorescences (main florescences)* consisting of a pedunculate double-cincinni fused back to back; peduncles 1.9–3.7 cm long, dark green to vinaceous, glabrous, except for a leaf-opposed longitudinal line of short, uniseriate, light brown to hyaline hairs; basal bract inconspicuous, tubular, hyaline, glabrous; peduncle bracts absent; supernumerary bracts absent; cincinni bracts 1.2–3.4 × 0.4–1.3 cm, leaf-like, unequal to strongly unequal to each other, elliptic to narrowly ovate to ovate, velutine to hispid on both sides, rarely hairs restricted to the midvein, hairs light brown to hyaline, adaxially dark green, abaxially green with vinaceous blotches, base cordate to round, saccate, margin ciliolate, slightly revolute, apex acuminate; double-cincinni (4–)6–8-flowered; bracteoles inconspicuous, imbricate, linear-triangular to triangular, hyaline. *Flowers* 1.1–1.3 cm diam.; floral buds broadly ovoid, apex acute; pedicels 1.2–3.4 mm long, upright at anthesis and pre-anthesis, reflexed at post-anthesis, vinaceous, densely glandular-pubescent, rarely with a mixture of glandular and eglandular, hyaline hairs; sepals 3.8–5.3 × 2.6–4.2 mm, dorsally keeled, with a mixture of glandular and eglandular, hyaline hairs restricted to the keel, hairs hyaline to light brown; petals 6.6–8.2 × 3.7–5.2 mm, plicate, white; filaments 3.6–4.9 mm long, anthers 0.4–0.6 × 0.5–0.6 mm; ovary 0.9–1.1 × 0.8–1.2 mm, style 3.8–4.0 cm long, pistil the same length as the stamens. *Capsules* 3–3.5 × 2.1–2.4 mm. *Seeds* 1.4–1.6 × 1.1–1.3 mm, greyish-brown to brown, not cleft towards the embryotega, rugose; hilum shorter than ½ the length of the seed.

##### Specimens seen (paratypes).

**BRAZIL. Minas Gerais**: Tiradentes, caminho para a Serra de São José, fl., fr., 16 Jan 1994, A.M. Giulietti et al. 13666 (K, SPF). **Rio de Janeiro**: Itatiaia, Parque Nacional do Itatiaia, Planalto, proximidades do Brejo da Lapa, fl., fr., 3 Dec 1996, J.M.A. Braga et al. 3708 (RB); Nova Friburgo, Furnas do Catete, fl., fr., 4 Apr 1965, J.C. Siqueira 2032 (FCAB); fl., fr., 3 Jun 1987, L.C. Giordano & D.P. Costa 313 (RB). **São Paulo**: São Paulo, Santo Amaro, Seminário Espírito Santo, fl., fr., 20 Oct 1943, L. Roth 812 (SP); Parque Estadual da Serra do Mar, margem esquerda do Rio Quilombo, fl., 9 Oct 1989, D.G. Scaravelli s.n. (ESA no. 5253, RB no. 628820); Tapiraí, Cachoeira do Chá, fl., 18 Oct 1994, K.D. Barreto et al. 3056 (ESA, RB, SPVR, US).

##### Distribution and habitat.

*Tradescantia atlantica* is endemic to Brazil, more precisely to the states of Minas Gerais, Rio de Janeiro and São Paulo; in the Atlantic Forest domain (Fig. 9). It can be found growing as a terrestrial understorey in shaded and moist forests.

##### Phenology.

It was found in bloom and fruit from October to June but peaking during January.

##### Etymology.

The epithet makes reference to this species’ distribution range, restricted to the Atlantic Forest domain.

##### Conservation status.

*Tradescantia atlantica* possesses a wide EOO (ca. 60,715.793 km^2^), but a considerably narrow AOO (ca. 32.000 km^2^). Since it is only known from seven very fragmented collections, following the IUCN (2001) recommendations, *T. atlantica* should be considered Endangered [EN, A2cde+B2ab(ii, iii, iv)+D2].

##### Comments.

**Figure 8.**
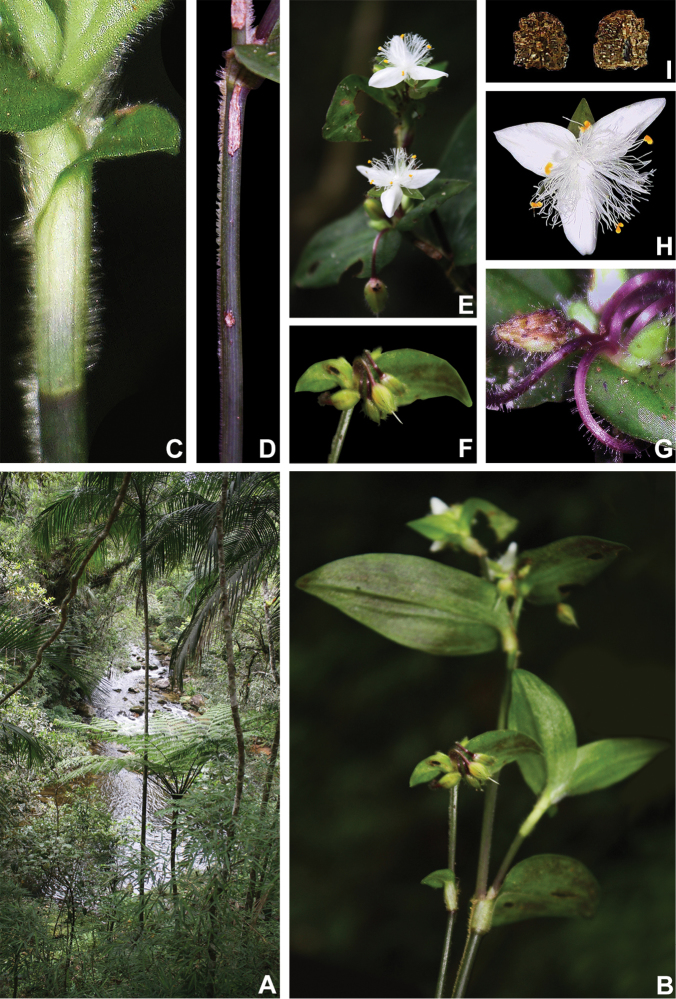
*Tradescantia atlantica* M.Pell. **A** Estação Ecológica de Macaé de Cima, Nova Friburgo, state of Rio de Janeiro, Brazil **B** habit, showing the erect, fibrous and little branched stems **C** young shoot, showing the densely hispid indumentum **D** mature stem, showing the dark green to vinaceous and glabrous internodes with the leaf-opposed line of uniseriate hairs **E–F** inflorescence **E** overview of the inflorescence **F** detail of the main florescence, showing the unequal cincinni bracts **G** detail of the pedicels, showing the vinaceous colouration and glandular hairs **H** front view of a flower, showing the plicate petals **I** dorsal and ventral views of the seed, showing the rugose testa not cleft towards the embryotega and the hilum shorter than ½ the length of the seed. Photos by M.O.O. Pellegrini.

*Tradescantia atlantica* is a member of the *T. tenella* species complex, being morphologically similar to *T. tenella* and *T. tucumanensis*, due to its definite base, conspicuous secondary veins (Fig. 8B), saccate and unequal to strongly unequal cincinni bracts (Fig. 8F), keeled sepals (Fig. 8G), pistil the same length as the stamens (Fig. 8H), seeds with rugose testa and hilum shorter than ½ the length of the seed (Fig. 8I). It was previously tentatively included by me (Pellegrini 2015) under a much broader *T. tenella*, due to its erect stems and ovoid floral buds. Nonetheless, after further herbarium and field studies, I have come to the conclusion it indeed merits taxonomic recognition. The fibrous stems and sessile leaves (Fig. 8B) vegetatively differentiate *T. atlantica* from *T. tenella*, with the fibrous stems being unique in the subgenus. Also, the velutine to hispid indumentum covering the leaves (Fig. 8C) is different from the much coarser indumentum observed in *T. tenella* (Fig. 2E), while the indumentum in the sepals is composed of a mixture of glandular and eglandular hairs (Fig. 8G), as opposed to the evenly glandular pubescent sepals of *T. tenella* (Fig. 2F). Finally, the plicate petals of *T. atlantica* (Fig. 8H) are only observed in the not so closely related *T. fluminensis* (Fig. 6E) and the distribution of *T. atlantica* and *T. tenella* has no overlaps.

*Tradescantia atlantica* can also be differentiated from the new *T. tucumanensis* by its fibrous stems (*vs.* succulent in *T. tucumanensis*), sessile leaves with velutine to hispid indumentum (*vs.* at least the basal ones subpetiolate, sparsely hirsute to hirsute), broadly ovoid floral buds (*vs.* ellipsoid), sepals with a mixture of glandular and eglandular hairs restricted to the keel (*vs.* with a mixture of glandular and eglandular hairs, but exclusively hispid along the keel in *T. tucumanensis*) and petals always white and plicate (*vs.* ranging from white to pink and flat).

**Figure 9.**
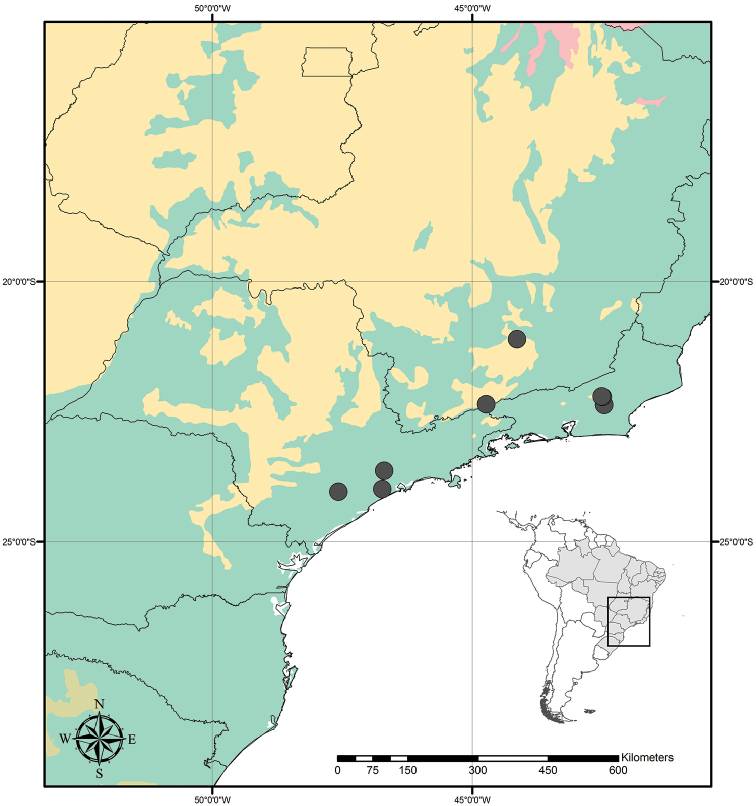
Distribution of *Tradescantia atlantica* M.Pell. Orange– Cerrado; Red– Caatinga; Dark green– Atlantic Forest.

#### 2. Tradescantia cerinthoides

Taxon classificationPlantaeORDOFAMILIA

Kunth, Enum. Pl. 4: 83. 1843.

Figs 10 , 11

Tradescantia blossfeldiana Mildbr., Notizbl. Bot. Gart. Berlin-Dahlem 15: 222. 1940. Neotype (designated here). ARGENTINA. Originally cultivated at the Botanischer Garten und Botanisches Museum Berlin-Dahlem, cuttings sent to Royal Botanical Gardens, Kew, by W. Curtis, fl., 19 Mar 1951, H. Blossfeld s.n. (K barcode K000501910!; isoneotype: K barcode K000501909!).
Tradescantia crassula var. gaudichaudii C.B.Clarke in De Candolle & De Candolle, Monogr. Phan. 3: 294. 1881. Lectotype (designated here). BRAZIL. Santa Catarina: s.loc., fl., s.dat., C. Gaudichaud 112 (P barcode P02173932!; isolectotype: P barcode P02173933!). **Syn. nov.**
Tradescantia cymbispatha var. villosissima C.B.Clarke in De Candolle & De Candolle, Monogr. Phan. 3: 296. 1881. Lectotype (designated here). BRAZIL. Provincia de São Paulo, fl., fr., 1816–1821, A. Saint-Hilaire C2 1500 (P barcode P02174044!; isolectotype: P barcode P02174047!).
Tradescantia koernickeana Seub. in Martius, Fl. bras. 3(1): 249. 1855. Lectotype (designated by Pellegrini et al. 2016) BRAZIL Rio Grande do Sul: Rio Pardo, fl., 23 Sep 1833, F. Sellow 3033a (B barcode B100521013!; isolectotypes: K barcodes K001040251!, K001096644!, P barcode P02174008!).

##### Type material.

Lectotype **(designated by Pellegrini et al. 2016).** BRAZIL. Brasilia meridionalis, fl., fr., Dec 1836, F. Sellow 2963 (B barcode B100521011!; isolectotypes: B barcode B100521012!, K barcode K000363273!; MO barcode MO3021307!).

##### Description.

*Herbs* ca. 10–60 cm tall, with a definite base, terrestrial or rupicolous, rarely epiphytes. *Stems* erect, succulent, little branched, branching at the base, rarely branching at the upper half; internodes 1–7.4 cm long at base, distally shorter, green with vertical reddish-purple striations to vinaceous, glabrous to velutine to hirsute to glandular-pubescent, light-brown to hyaline hairs. *Leaves* distichously-alternate to spirally-alternate, sessile; ptyxis convolute; sheaths 0.3–1.3 cm long, green to pink to vinaceous, glabrous or velutine to hispid, margins densely setose to hispid, hairs hyaline to light brown to golden, sometimes also with some glandular hairs; blades 1.5–17.5 × 0.6–3 cm, elliptic to broadly elliptic to ovate to broadly ovate to obovate to broadly obovate, falcate to complicate, succulent, velutine to hispid on both sides or adaxially glabrous to sparsely hispid, abaxially hispid, hairs hyaline to light brown to golden, commonly also with a mixture of glandular hairs, adaxially light to medium to dark green, sometimes with vinaceous stripes, abaxially green to vinaceous, turning olive-green to brown when dry, base cordate to obtuse, rarely cuneate, margin green to vinaceous, ciliolate to ciliate, slightly revolute, apex acute to obtuse; midvein conspicuous, adaxially impressed, secondary veins conspicuous, adaxially slightly impressed, abaxially slightly impressed, becoming more evident on both sides when dry. *Synflorescences* terminal or axillar in the distal portion of the stems, composed of a solitary main florescence, 1 per leaf axis. *Inflorescences (main florescences)* consisting of a pedunculate double-cincinni fused back to back; peduncles 0.4–5.5 cm long, green to vinaceous, glabrous to velutine to hispid, hairs hyaline to light brown to golden, commonly also with a mixture of glandular hairs; peduncle bracts absent; supernumerary bracts absent; cincinni bracts 0.8–5.1 × 0.5–2.1 cm, leaf-like, similar to each other, broadly elliptic to ovate to broadly ovate, velutine to hispid on both sides or adaxially glabrous to sparsely hispid, abaxially hispid, hairs hyaline to light brown to golden, adaxially light to medium to dark green, rarely with vinaceous stripes, abaxially green to vinaceous, base cordate to obtuse, not saccate, margin ciliolate to ciliate, slightly revolute, apex acute to obtuse; double cincinni 6–22-flowered. *Flowers* 1.3–1.6 cm diam., pedicels 0.5–2 cm long, green to vinaceous, velutine to hispid, hairs hyaline to light brown, commonly also with a mixture of glandular hairs; floral buds ovoid; sepals 5–7.8 × 2.2–3.4 mm, not keeled, green to vinaceous, velutine to hispid, commonly also with a mixture of glandular hairs, hairs hyaline to light brown, rarely golden; petals 4.9–7.8 × 4.4–7.2 mm, flat, white or white with pink apex to light pink to pink to lilac; filaments 4.7–6.7 mm long, anthers 0.8–1 × 1–1.4 mm; ovary 1–1.5 × 0.9–1.5 cm, style 2.9–5.7 cm long; pistil longer than the stamens. *Capsules* 3.5–4.5 × 2.3–3.6 cm. *Seeds* 1.2–2.2 × 0.9–1.7 mm, testa medium to dark grey, cleft towards the embryotega, costate; hilum longer than ½ the length of the seed.

##### Specimens seen.

**ARGENTINA. Without province**: s.loc., fl., 27 Nov 1936, W.A. Archer 4594 (US); fl., 27 Apr 1961, F. Mennega 3801 (U); fl., 11 Aug 1967, Delfk s.n. (L barcode L1432944). **Buenos Aires**: s.loc., fl., s.dat., N.M. Bacigalupo 351/67 (K, SI); Isla Martín García, fl., fr., 23 Jan 1997, J. Hurrell & M. Belgrano 3415 (LP); La Plata, Gonnet, 15 bis entre Papini y Bordenave, fl., 26 Nov 2000, Delucchi 2451 (LP). **Corrientes**: Capital, Corrientes, fl., fr., 30 Nov 1972, M.C. Kirchmair 4 (CORD, CTES). **Distrito Federal**: Buenos Aires, fl., 16 Dec 2005, M. Grabiele 27 (CORD). **Misiones**: Departamento de Guarani, Arroyo El Paraiso y ruta, fl., 23 Sep 1993, M. Rodriguez et al. 704 (US); Eldorado, Salto Küppers, fl., 26 Aug 2002, M. Grabiele 13 (CORD). **BRAZIL. Without province**: s.loc., fl., fr., s.dat., s.leg. 66 (K barcode K001248110); fl., Nov 1875, F. Lauth 7588 (P); cultivated at the Missouri Botanical Garden, fl., 28 Mar 1972, W.G. D’Arcy 5788 (INPA). **Minas Gerais**: São João Del Rei, fl., Oct 1969, J. Mattos s.n. (CESJ no. 67765). **Paraná**: Balsa Nova, Campina da Cascavel, fl., 8 Nov 1976, G. Hatschbach 39180 (MBM, MO, US); Chácara Payquera, fl., fr., 8 Jan 2003, E.E. Kauano 22 (MBM); Ponte dos Arcos, fl., 13 Oct 2015, C. Kozera 2416 & A. Sanches (MBM); Sprea, fl., 25 Oct 1973, G. Hatschbach 32951 (K, MBM); Rodovia BR-277, Serra São Luiz do Purunã, fl., fr., 12 Dec 1965, R. Reitz & R.M. Klein 17441 (HBR, P, US); fl., fr., 28 Oct. 1996, O.S. Ribas & M.F. Luz 1531 (MBM); fl., fr., 7 Nov 1998, W. Amaral & D. Dunaiski 384 (MBM); região dos Campos Gerais, fl., fr., 6 Oct 2012, F. Santos-Silva et al. 166 (RB, UPCB); Candói, Três Pinheiros, fl., fr., 18 Jun 2004, R. Goldenberg 630 et al. (UPCB, MBM); Vale do Rio Iguaçu, Barra do Rio Jordão, fl., fr., 23 Feb 1996, G. Hatschbach 64469 et al. (MBM); Castro, Carambeí, Rio São João, fl., fr., 3 Oct 1964, G. Hatschbach 11674 (MBM, US); Curitiba, estrada Curitiba-Ponta Grossa km 38, Serra São Luiz de Purunã, fl., fr., 18 Oct 1961, E. Pereira 6081 (RB); fl., fr., 18 Oct 1961, E. Pereira & G. Pabst 6084 (RB); Guarapuava, Canta Galo, fl., fr., 4 Feb 1969, G. Hatschbach & P.F. Ravenna 23112 (MBM, MO, UEC); Colônia São Judas Tadeu, fl., fr., 8 Dec 1982, G. Hatschbach 45805 (CORD, MBM, US); Lapa, Gruta do Monge, fl., fr., 1 Dec 1982, P.I. Oliveira 711 (MBM, MO, US); Serrinha, fl., fr., 17 Oct 1948, G. Hatschbach 1065 (MBM); Palmeira, Fazenda Santa Rita, fl., fr., 22 Nov 1987, L.T. Drombowski 14087 (MBM); Piraí do Sul, Fazenda Santa Rita, fl., 2 Nov 1998, O.S. Ribas 2812 (MBM); Ponta Grossa, fl., fr., 18 Oct 1965, G.L. Monteiro s.n. (RFA no. 7951); fl., Nov 1969, L. Krieger 7344 (CESJ, K); fl., 18 Dec 1971, L. Krieger 11280 (CESJ, K); Parque Estadual de Vila Velha, fl., fr., 21 Jan 1965, L.B. Smith & R.M. Klein 14911 (HBR, P, R); fl., fr., 9 Nov 1966, P. Occhioni 3487 (RFA); fl., fr., 15 Oct 1989, A.C. Cervi et al. 2820 (MBM); fl., 14 Oct 1997, A.C. Cervi 6358 (UPCB); próximo à Fortaleza, fl., 9 Nov 2002, R. Gonçalves 25 (UPCB); fl., 2 Feb 2011, G.A. Dettke 565 (ICN); fl., 17 Oct 2013, J.M. Silva & J.T. Motta 8377 (MBM); Buraco do Padre, fl., 27 Oct 1995, O.S. Ribas & L.B.S. Pereira 847 (MBM); Cachoeira da Mariquinha, fl., fr., 4 Nov 2010, E.L. Siqueira et al. 399 (HCF, MBM); Prudentópolis, Salto São João, fl., 16 Oct 2005, S.L. Jung-Mendaçolli 1148 (IAC); Serrinha, fl., 26 Nov 1911, P. Dusén 13449 (MBM, NY, S); Rio Bonito do Iguaçu, Rio Iguaçu, fl., 21 Jun 1995, C.B. Poliquesi & E. Barbosa 293 (MBM); Tibagí, fl., fr., 11 Oct 1959, G. Hatschbach 6382 (MBM, US); Guartelá, Canyon do Guartelá, fl., fr., 4 Nov 1994, M.E. Buim et al. s.n. (FUEL no. 14199); Canyon Rio Iapó, fl., fr., 10 Nov 1992, G. Hatschbach & E. Barbosa 58160 (MBM, US); margem direita do Rio Tibagí, próximo à ponte, fl., fr., 7 Oct 1994, D.C. Lemos et al. s.n. (FUEL no. 14551); Ventania, Fazenda Santa Inês, fl., 19 Oct 2005, D.A. Estevan 979 (FUEL); Vila Velha, Parque Estadual de Vila Velha, fl., fr., 12 Jul 1962, Gomes & J. Mattos 1139 (RB). **Rio Grande do Sul**: s.loc., fl., 1833, C. Gaudichaud 1794 (P); fl., 23 Sep 1833, F. Sellow 3033 (K, P); NE region montanosa, fl., s.dat., A.E. Burkart 25565 (CORD, K); Arroio dos Ratos, Granja Faxinal, fl., Oct 1976, K. Hagelund 10514 (ICN); Bagé, ca. 12 km de Aceguá, fl., Apr 1985, J. Mattos et al. 28847 (HAS); Barão, sudoeste de Garibaldi, estrada para Carlos Barbosa, fl., fr., 22 Nov 2005, M.C. Machado & L.Y.S. Aona 606 (HUEFS, UEC); fl., fr., 22 Nov 2005, M.C. Machado & L.Y.S Aona 607 (HUEFS, UEC); Bom Jesus, Serra da Rocinha, fl., Nov 1987, J. Meyer et al. 201 (HAS); Caçapava do Sul, Pedra do Segredo, fl., fr., 22 Nov 2005, M.C. Machado & L.Y.S. Aona 621 (HUEFS, UEC); Cambará do Sul, Fortaleza, fl., Apr 1982, J. Mattos & N. Silveira 23312 (HAS); RS-453, ca. 7.4 km a leste do entroncamento da RS-453 com a RS-020, fl., 23 Nov 2005, M.C. Machado & L.Y.S. Aona 603 (HUEFS, UEC); fr., 18 Nov 2008, J.M. Silva et al. 7342 (MBM); Capão da Canoa, estrada entre Riozinho e Maquiné, ca. 22 km leste de Riozinho, fl., fr., 22 Nov 2005, M.C. Machado & L.Y.S. Aona 595 (HUEFS, UEC); Caxias do Sul, estrada para Mulada, fl., fr., 10 Dec 2005, M.C. Machado & L.Y.S. Aona 739 (HUEFS, UEC); Cruz Alta, 10 km S de Cruz Alta, fl., fr., A. Krapovickas & R. Vanni 36768 (CTES); Morro dos Conventos, fl., fr., 8 Nov 1968, A.R. Schultz et al. 5442 (CORD, CTES, ICN); Erechim, campus da URI, fl., 6 Sep 1993, A. Butzke et al. 7140 (US); Farroupilha, fl., 10 Nov 1957, Camargo 2464 (PACA); Garibaldi, fl., 29 Oct 1957, Camargo s.n. (PACA 62807); Guaíba, Fazenda Maximiano, próximo do banhado, fl., 10 Dec 2015, M.O.O. Pellegrini & R.F. Almeida 477 (RB); Itapuã, Granja Neugebauer, fl., fr., 11 Oct 1950, B. Rambo 48966 (HBR, K, LIL); Montenegro, fl., 19 Sept 1957, Camargo 1785 (PACA); Fortaleza, fl., fr., 15 Dec 1952, B. Rambo 52904 (PACA); Nova Prata, ca. 9 km de Nova Prata, em direção à Cascata, fl., fr., Nov 1982, J. Mattos & R. Frosi 23749 (HAS); Pareci Novo, fl., 7 Sep 1949, B. Rambo 43789 (PACA); Pinhal, fl., 25 Nov 1949, A. Sehnem s.n. (PACA no. 47744); Pinheiro Machado, km 13 da rodovia para Pelotas, fl., fr., J. Mattos & N. Silveira 25312 (HAS); Piratini, Fazenda Cerro Verde, fl., fr., 1 Nov 1998, L.P. Félix 8995 (HST, RB); Porto Alegre, Morro do Osso, fl., 21 Oct 1944, B. Rambo s.n. (PACA 44043); fl., 9 Oct 1947, I. Granck s.n. (PACA no. 37124); Morro da Polícia, fl., 9 Sep 1949, B. Rambo 43324 (PACA); Morro Santana, fl., 6 Nov 1932, B. Rambo 69 (K, LIL, PACA); fl., 1947, K. Emrich s.n. (PACA no. 34269); Vila Manresa, fl., 27 Nov 1945, B. Rambo 30624 (PACA); fl., 27 Nov 1945, B. Rambo 30669 (K, LIL, PACA); fl., 1948, B. Rambo 37887 (PACA); fl., 1 Oct 1948, B. Rambo 37779 (PACA); fl., 18 Oct 1950, B. Rambo 49022 (PACA); fl., 22 Oct 1955, B. Rambo 57077 (PACA); fl., fr., 21 Oct 1951, B. Rambo 51329 (PACA); Universidade Federal do Rio Grande do Sul, Campus do Vale, escadaria do campus, próximo ao ponto de ônibus, fl., 9 Oct 2014, M.O.O. Pellegrini & F. Santos-Silva 448 (RB); São Francisco de Paula, Linha Feixe, fl., fr., 17 Oct 2003, R.A. Wasum & J. Bordin 1995 (HUCS, K, US); Taimbesinho, fl., 13 Nov 1953, B. Rambo 54493 (PACA); São João do Polêsine, fl., 6 Oct 2011, G.A. Dettke & J. Durigon 951 (ICN); São Leopoldo, arredores de São Leopoldo, fl., Oct 1941, J. Eugênio 433 (NY); São Marcos, km 138 da rodovia Porto Alegre-Vacaria, fl., fr., J. Mattos 20332 (HAS); Soledade, rodovia Porto Alegre-Sarandi, km 232, fl., Nov 1983, J. Mattos et al. s.n. (HAS no. 67962); Vacaria, BR-116, km 57, divisa com Lages, fl., Oct 1982, L.A. Cestaro s.n. (HAS no. 28451); fl., fr., 12 Dec 2015, M.O.O. Pellegrini & R.F. Almeida 482 (RB). **Santa Catarina**: s.loc., fl., fr., s.dat., D. D’Urville s.n. (P barcodes P02174006, P02173934); Alfredo Wagner, Alto Limeirinha, fl., fr., 25 Nov 2009, A. Korte & A. Kniess 189 (FURB); Araranguá, Sombrio, fl., 19 Oct 1944, R. Reitz c781 (HBR, RB); Florianópolis, Morro da Cruz, fl., fr., Dec 1984, J. Mattos & N. Silveira 28717 (HAS); Rio Vermelho, fl., fr., 23 Nov 1965, R.M. Klein & A. Bresolin 6335 (HBR); Içara, Balneário Rincão, fl., fr., 9 Dec 2010, A. Korte & M.J. Rigon Jr. 5538 (FURB, RB); Laguna, Morro da Glória, fl., fr., 9 Dec 2015, M.O.O. Pellegrini & R.F. Almeida 472 (RB); Palhoça, Campo do Massiambú, fl., 4 Nov 1953, R. Reitz & R.M. Klein 1325 (HBR); fl., fr., 4 Nov 1953, R. Reitz & R.M. Klein 1343 (HBR); Pedra do Urubu, fl., fr., 3 Dec 2010, A. Korte 5432 (FURB); Parque Estadual do Tabuleiro, fl., fr., 2 Dec 2010, A. Korte 5318 (FURB); Guarda do Embaú, fl., 1 Apr 2013, L.A. Funez & A.E. Zermiani 2015 (FURB); Passo de Torres, fl., fr., s.dat., A.E. Burkart 25584 (CORD, K); Parque Estadual da Guarita, mata ao longo da escadaria para o mirante, fl., 8 Dec 2015, M.O.O. Pellegrini & R.F. Almeida 475 (RB); Urubici, estrada Serra do Corvo Branco, fl., fr. 6 Dec 2005, J.R. Stehmann et al. 1752 (UEC); Comunidade São Pedro, fl., fr., 15 Nov 2008, J.M. Silva et al. 7172 (MBM). **São Paulo**: Atibaia, Pedra Grande, topo do morro, fl., fr., 29 Nov 1961, J. Mattos 9525 (K, RB, SP); Campos do Jordão, cultivada em Vinhedo, Condomínio Marambaia, fl., fr., 10 Aug 2004, S.L. Jung-Mendaçolli 1137 (IAC). **URUGUAY. San José**: Balneario Kiyú, Río de la Plata, fl., fr., 21 Nov 2007, G.J. Seijo & V. Solis Neffa 3971 (CORD, CTES, FUEL).

##### Distribution and habitat.

*Tradescantia cerinthoides* is known to occur in Argentina, Brazil (states of Minas Gerais, São Paulo, Paraná, Santa Catarina and Rio Grande do Sul) and Uruguay; in the Atlantic Forest, Cerrado, Chaco and Pampa domains (Fig. 11). It can be found in grasslands growing in full sun or in shaded conditions, directly over rock or as a terrestrial plant. It can be also found growing in sand dunes and in *restinga* formations in Southern Brazil.

##### Phenology.

It was found in bloom and fruit throughout the year but peaking during the rainy season and being less commonly found in bloom during the dry season.

##### Etymology.

The epithet “*cerinthoides*” means “similar to pollen grains”, probablymaking reference to the moniliform hairs of the filaments. These hairs are theorised by Faden (1992) to simulate pollen grains and deceive pollinators into visiting the flowers of Commelinaceae.

##### Conservation status.

*Tradescantia cerinthoides* possesses a wide EOO (ca. 945,153.803 km^2^), being widely cultivated worldwide as an ornamental plant and being potentially an invasive species in the same regions as *T. fluminensis*. In its natural habitats, *T. cerinthoides* forms dense subpopulations, reproducing either by clones or sexually by seeds. Thus, following the IUCN recommendations (IUCN 2001), *T. cerinthoides* should be considered Least Concern (LC).

##### Nomenclatural notes.

*Tradescantia blossfeldiana* was described by Mildbraed (1940) based on cultivated material by H. Blossfeld at the Botanischer Garten und Botanisches Museum Berlin-Dahlem (Germany), and originally collected in Argentina. Mildbraed gives a detailed description that gives me no doubt that this species should be treated as a synonym of *T. cerinthoides*, as proposed by Hunt (2001). Nonetheless, Mildbraed (1940) cites no examined material. It is known that Mildbraed worked in Berlin (Stafleu and Cowan 1981), however no specimen matching the protologue was ever found at B. Stearn (1955) published a beautiful watercolour for *T. blossfeldiana*, together with horticultural comments for this species in Curtis’s Botanical Magazine. According to Stearn (1955), the watercolour presented by him was based on the living specimen, still in cultivation at the time at the Botanischer Garten und Botanisches Museum Berlin-Dahlem, which served as the base for Mildbraed’s description. Cuttings from the original specimens at the Botanischer Garten und Botanisches Museum Berlin-Dahlem were then sent to the Royal Botanical Gardens, Kew, by Dr. William Curtis on 1931. The plants flowered several times and vouchers were done in 1931, 1939, 1940, and 1951 and placed at K. After careful study of these voucher specimens at K, I have chosen the specimens collected in 1951, since one of the sheets is clearly the one on which the watercolour, published by Stearn (1955), was based. Thus, specimen K000501910 is here designated as the neotype of *T. blossefeldiana*, while specimen K000501909 is treated as the isoneotype.

##### Comments.

**Figure 10.**
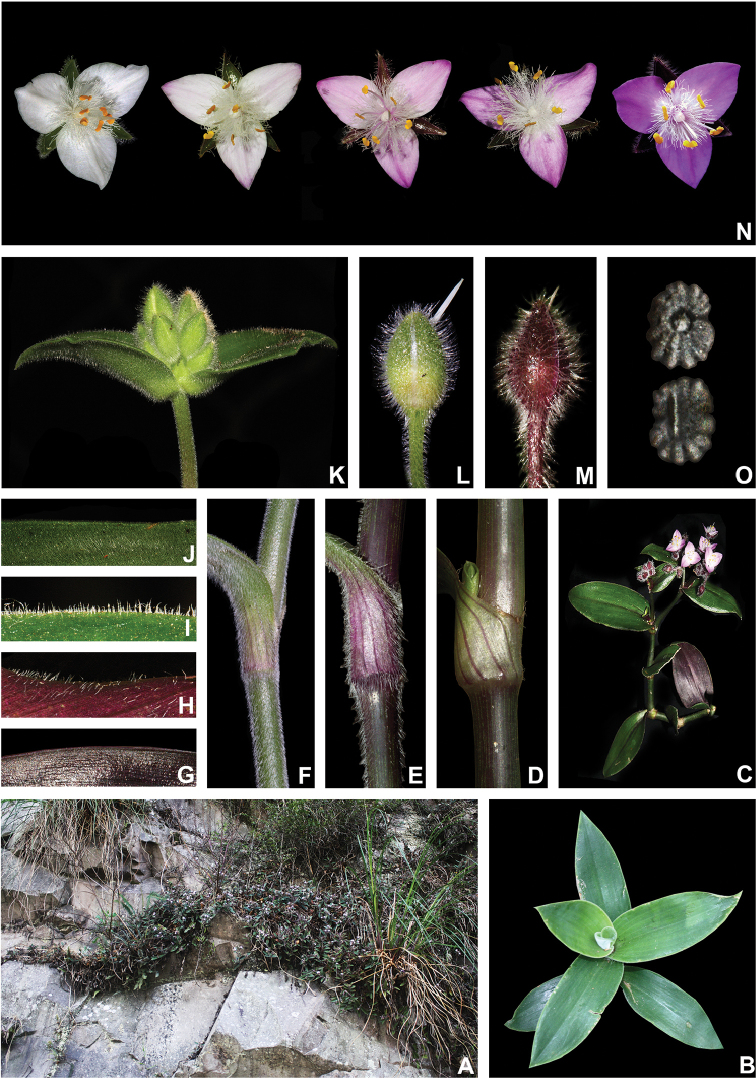
*Tradescantia cerinthoides* Kunth. **A** rupicolous subpopulation in the municipality of Vacaria, state of Rio Grande do Sul, Brazil **B–C** habit **B** habit, showing the spirally-alternate leaves **C** habit, showing the distichously-alternate leaves **D–F** stems and leaf-sheaths **D** detail of a glabrous stem, vinaceous with green striations and sparsely hispid leaf-sheath, light green with vinaceous striations **E** detail of a hispid stem, vinaceous with green striations and hispid leaf-sheath, vinaceous with darker striations **F** detail of a velutine stem, medium green and velutine, pinkish leaf-sheath with vinaceous striations **G–J** pubescence of the leaf-blades **G** abaxial side, showing the dark vinaceous colouration and lack of pubescence **H** abaxial side, showing the vinaceous colouration and sparsely hispid indumentum **I** adaxial side, showing the medium green colouration and the hispid indumentum **J** abaxial side, showing the medium green colouration and velutine indumentum **K** inflorescence, showing the non-saccate cincinni bracts and velutine indumentum **L–M** floral buds **L** green floral bud, showing the velutine indumentum **M** vinaceous floral bud, showing the hispid indumentum **N** front view of flowers from the same subpopulation (Vacaria, Rio Grande do Sul, Brazil), showing the variation of sepal colour (green to dark vinaceous), sepal pubescence (velutine to sparsely hispid to hispid to densely hispid) and petal colour (white to shades of pink to lilac) **O** dorsal and ventral views of the seed, showing the costate testa cleft towards the embryotega and the hilum longer than ½ the length of the seed. Photos by M.O.O. Pellegrini.

*Tradescantia cerinthoides* is a member of the *T. crassula* group, due to its erect stems (Fig. 10A), definite base (Fig, 10B), convolute ptyxis (Fig, 10B), complicate and/or falcate leaves (Fig. 10B, C), cincinni bracts not saccate at base (Fig. 10K), petals that range from white to pink to lilac (Fig. 10N), pistil longer than the stamens (Fig. 10N), seed cleft towards the embryotega and hilum longer than ½ the length of the seeds (Fig. 10) (Pellegrini 2015, 2016, 2017). It can be easily differentiated from the remaining species of this group by a combination of: sepals not keeled and evenly pubescent (with indumentum ranging from velutine to hispid and generally with a mixture of glandular and eglandular hairs; Fig. 10L, M). It is highly polymorphic, being together with *T. crassula*, the only two species in the subgenus where the phyllotaxy has been observed to vary in adult specimens. The individuals presenting spirally-alternate leaves with shortened internodes (i.e. producing rosette leaves) and white petals represent the morphological variation described by Seubert (1855) as *T. koernickeana*. Also, its leaves are generally densely covered by indumentum on the abaxial side, but some individuals with completely glabrous leaves can also be found. On the other hand, the specimens with spirally-alternate leaves and elongated internodes represent *T. cerinthoides* as originally described by Kunth (1843). Finally, the specimens with distichously-alternate leaves, with blades generally adaxially green with vinaceous stripes, glabrous or sparsely pubescent, abaxially vinaceous and densely pubescent and petals ranging from pink to lilac, represent the morphological variation described by Mildbraed (1940) as *T. blossfeldiana*. Nonetheless, *T. cerinthoides* shows great morphological variation in the same subpopulation. The same subpopulation can present individuals from all three aforementioned morphotypes growing together and, more importantly, with all kinds of intermediate forms between them (Fig. 10N). The same wide morphological variation was also observed in cultivation, with all morphs crossing and producing viable seeds (pers. observ.). Some of the morphological variation observed in *T. cerinthoides* can be partially explained by environmental features (e.g. plant stature and growth form, overall plant succulence, leaf shape and colouration etc.). Nonetheless, most of the obviously observed morphological variation seems to have at least a partial genetic background, with characters such as indumentum of the vegetative organs and colouration of the petals being maintained regardless of the environment. This still poorly understood variation seems to be the main cause of the description for all of its species synonyms and also as a cause for this species being so popular in cultivation. In this scenario, it seems illogical to accept several ill-defined species based on non-clear-cut character states, instead of the broader *T. cerinthoides* as proposed by Hunt (1980, 2001). Populational studies, coupled with reproductive and morphometrical studies, are needed to help us better understand this species’ morphological plasticity. *Tradescantia cerinthoides* is the most popular species from the *T. crassula* group, as a potted plant. This is especially due to the beautiful pink to lilac flowers that are common in the cultivated specimens and its generally dense leave indumentum.

**Figure 11.**
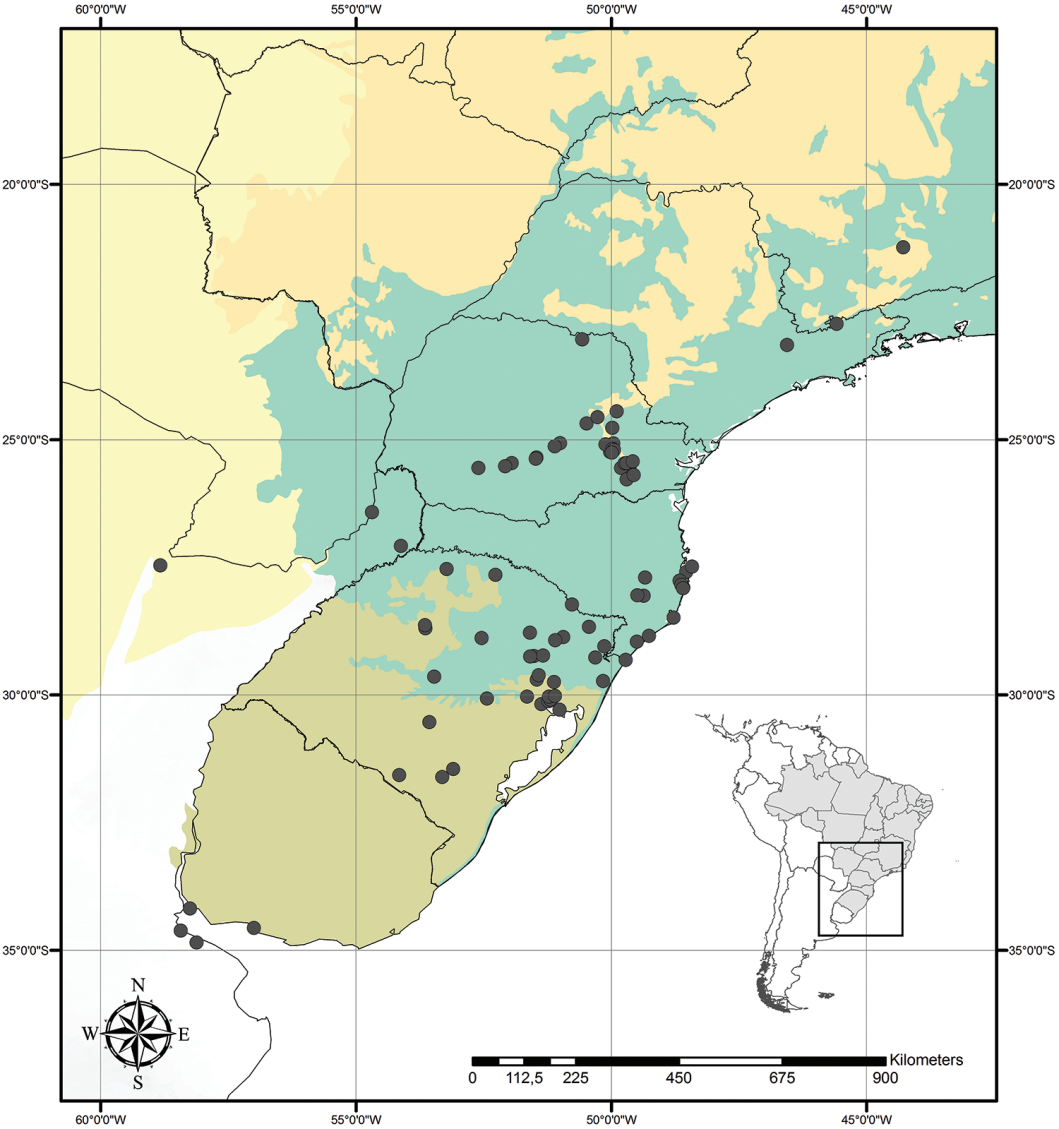
Distribution of *Tradescantia cerinthoides* Kunth. Orange– Cerrado; Yellow– Chaco and Pantanal; Olive-green– Pampa; Dark green– Atlantic Forest.

#### 3. Tradescantia chrysophylla

Taxon classificationPlantaeORDOFAMILIA

M.Pell., PhytoKeys 80: 9. 2017.

Figs 12 , 13

Tradescantia serrana Hassemer & Funez, in Hassemer et al. Phytotaxa 312 (2): 214. 2017. Holotype. BRAZIL. Santa Catarina: Urubici, SC-110, entre Bom Retiro e Urubici, fl., 12 Feb 2016, G. Hassemer & L.A. Funez 888 (FURB no. 54402!; isotypes: B n.v., C n.v., W n.v.). **Syn. nov.**

##### Holotype.

BRAZIL. São Paulo: Biritiba Mirim, Estação Biológica de Boracéia, fl., 24 Nov 1983, A. Custódio Filho 1910 (RB barcode RB00972738!; isotype: SP barcode SP195458!).

##### Description.

*Herbs* ca. 10–40 cm tall, with an indefinite base, terrestrial or rupicolous, rarely epiphytes. *Stems* prostrate with ascending apex, delicate to slightly succulent, little to densely branched; internodes 1.5–8.2 cm long at base, distally shorter, medium to dark green or vinaceous, velutine to hispid, hairs golden to light brown. *Leaves* distichously-alternate, sessile; ptyxis involute; sheaths 0.4–1 cm long, light to medium green to vinaceous, sometimes with green striations, velutine to hirsute, margin densely setose, hairs golden; blades 1.8–7.6 × 0.9–3.4 cm, elliptic to broadly elliptic or lanceolate to ovate to broadly ovate, flat, succulent, velutine to hispid on both sides, hairs golden to light brown, adaxially medium to dark green, abaxially light to medium green or vinaceous, turning black to dark brown or olive-green when dry, base cordate to obtuse, margin ciliolate, slightly revolute, apex acute, sometimes acuminate; midvein conspicuous, adaxially impressed, secondary veins inconspicuous, adaxially inconspicuous, abaxially inconspicuous, becoming more evident abaxially when dry. *Synflorescences* terminal or axillar in the distal portion of the stems, composed of a solitary main florescence, 1 per leaf axis. *Inflorescences (main florescences)* consisting of a pedunculate double-cincinni fused back to back; peduncles (0.4–)1.1–9.5 cm long, velutine to hispid, hairs golden to light brown; cincinni bracts 0.9–6.6 × 0.3–3.1 cm, unequal to strongly unequal to each other, elliptic to ovate to broadly ovate, leaf-like, velutine to hispid, hairs golden to light brown, medium to dark green, abaxially light to medium green or vinaceous, base cordate to obtuse, saccate, margin ciliolate, slightly revolute, apex acute; double cincinni (4–)6–20-flowered. *Flowers* 1.1–1.6 cm diam., pedicels 0.6–1.3 cm long, glandular-pubescent; floral buds broadly ovoid; sepals 4.7–6 × 2.2–4 mm, not keeled, light to medium green, glandular-pubescent or with a mixture of glandular and eglandular, golden to light brown hairs; petals 7.2–9 × 4.6–6.2 mm, white; filaments 5.4–6.2 mm long, anthers 0.6–1 × 0.3–0.7 mm; ovary 0.8–1.7 × 0.7–1.4 mm, style 3.8–4.6 cm long; pistil the same length as the stamens. *Capsules* 2.7–3.2 × 2.2–2.8 mm. *Seeds* 1.1–1.5 × 1.0–1.4 mm, testa grey to greyish-brown, not cleft towards the embryotega, costate; hilum ½ the length of the seed.

##### Specimens seen.

**BRAZIL. Paraná**: Campo Largo, Caverna do Pinheirinho, fl., fr., 13 Oct 1996, G. Tiepolo & A.C. Svolenski 716 (EFC, MBM). Piraquara, Mananciais da Serra, fl., 10 Oct 1967, L.T. Dombrowski & Y.S. Kuniyoshi 2697 (MBM, US). Sapopema, Barra da Balsa Rio Tibagí, Fazenda Guaporé, fl., fr., 22 Jul 1989, A.O.S. Vieira et al. 366 (FUEL). São José dos Pinhais, Guaricana, 28 Sep 1983, P.I. Oliveira 746 (MBM, US). **Rio de Janeiro**: s.loc., fl., 1816-1821, A. Saint-Hilaire S44 (P barcodes P02174007, P02174010); Itatiaia, Serra do Itatiaia, Maromba, fl., 23 Oct 1931, C. Porto 2101 (RB); Resende, Parque Nacional do Itatiaia, Maromba, fl., fr., 14 Aug 1967, J. Mattos & N. Mattos 14774 (K, SP); Teresópolis, administração do Parque Nacional da Serra dos Órgãos, fl., 11 Jul 1956, A. Castellanos 21648 (RB); fl., 14 Jul 2011, J.A. Lombardi 8616 (HRCB); trilha na descida do abrigo 4, fl., 26 Oct 2012, D. Araújo et al. 116 (HRCB). **Rio Grande do Sul**: Guaíba, Parque Natural Municipal Morro José Lutzenberger, fl., 10 Dec 2015, M.O.O. Pellegrini & R.F. Almeida 480 (RB). **Santa Catarina**: Lages, Morro do Espigão, fl., fr., 25 Dec 1956, J. Mattos 3897 (HAS). Rio do Sul, estrada Rio do Sul-Lontras, fl., 3 Dec 2013, A.L. Gasper et al. 3270 (FURB). Rodeio, borda da floresta, fl., fr., 10 Oct 2015, L.A. Funez 4549 (FURB). Urubici, Salto do rio Avencal, fl., fr., 16 Oct 2004, G. Hatschbach et al. 78097 (MBM); São Miguel D’Oeste, forest above rio Reperi-guaçu, Peperi, fl., fr., 21 Oct 1964, L.B. Smith & R. Reitz 12775 (CORD, FLOR, HBR, K, NY, P, R, US). **São Paulo**: Jundiaí, mata de planalto na Serra do Japi, fl., 11 Aug 1976, H.F. Leitão-Filho & G.J. Shepherd 2536 (MBM, NY, UEC); ca. 10 km SW de Jundiaí, fl., fr., 8 Oct 1976, H.F. Leitão-Filho et al. 3175 (E, K, MBM, NY, UEC, UFG, US, VIC); Salesópolis, Estação Biológica de Boracéia, estrada para a barragem da SABESP no Rio Guaratuba, fl., 5 Sep 1994, R. Simão-Bianchini et al. 505 (RB, SP, UEC). São Paulo, Chácara dos Morrinhos, fl., 11 Oct 1941, B. Pickel 5444 (IPA, US); São Paulo, Serra da Cantareira, fl., Oct 1958, R. Schnell 8523 (P).

##### Distribution and habitat.

*Tradescantia chrysophylla* is endemic to Brazil, more precisely to the states of Rio de Janeiro, São Paulo, Paraná, Santa Catarina and Rio Grande do Sul; in the Atlantic Forest domain (Fig. 13). It can be found growing as a terrestrial, rupicolous or as an epiphyte understorey in shaded and moist forests.

##### Phenology.

It was found in bloom and fruit from July to December but peaking during October.

##### Etymology.

The epithet “*chrysophylla*” means “golden leaves” and is given after the golden hairs that cover the whole plant, but especially the leaves.

##### Conservation status.

According to Pellegrini et al. (2017), *T. chrysophylla* possesses a wide EOO (here updated to ca. 234,968.601 km^2^), but a considerably narrow AOO (ca. 60.000 km^2^). It is known from very few and fragmented collections and, following the IUCN (2001) recommendations, it should be considered as Endangered [EN, A2cde+B2ab(ii, iii, iv)+D2].

##### Comments.

**Figure 12.**
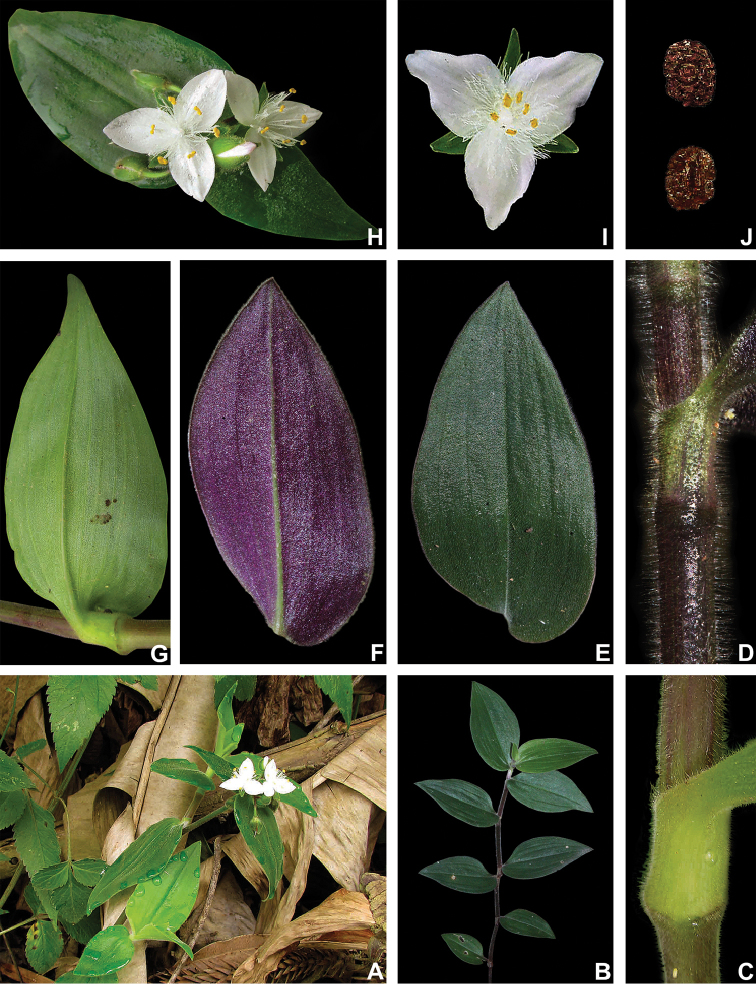
*Tradescantia chrysophylla* M.Pell. **A** habit, showing the prostrate stems **B** detail of the ascending apex **C–D** detail of the stem and leaf-sheath, showing the velutine to hispid indumentum **C** medium green to reddish-green internodes **D** vinaceous internodes **E–G** leaves **E** adaxial side **F** vinaceous abaxial side **G** light green abaxial side **H–I** inflorescence **H** upper view of an inflorescence, showing unequal cincinni bracts and a front view of a flower **I** front view of a flower. **A, C, G–I** by L.A. Funez; **B, D, E–F, J** by M.O.O. Pellegrini.

*Tradescantia chrysophylla* is morphologically similar to *T. cymbispatha*, *T. fluminensis* and *T. mundula* due to their indefinite base (Fig. 12, B), prostrate stems with ascending apex (Fig. 12A, B), involute ptyxis, saccate cincinni bracts, white petals (Fig. 12H, I), pistil as long as the stamens, seeds with uncleft testa towards the embryotega and hilum ½ the length of the seed (Fig. 12J). However, it can be easily differentiated from *T. fluminensis* and *T. mundula* by its sessile succulent leaves, blades evenly covered by indumentum and inconspicuous secondary veins (*vs.* leaves membranous, blades glabrous or unevenly covered by indumentum and impressed secondary veins), floral buds broadly ovoid (*vs.* narrowly ovoid to ovoid) and sepals without keels (*vs.* keeled sepals). *Tradescantia chrysophylla* is considerably more similar to *T. cymbispatha* due to their sessile, succulent leaves evenly covered by indumentum, inconspicuous secondary veins and sepals without keels. Nonetheless, in *T. chrysophylla*, the indumentum is velutine to hispid and golden to light brown but sometimes becoming light-brown when over-exposed to the sun (*vs.* strigose and hyaline in *T. cymbispatha*), the cincinni bracts are unequal to strongly unequal (*vs.* equal) and the pedicels and sepals are glandular-pubescent with golden to light brown hairs or covered by a mixture of glandular and eglandular hairs (*vs.* velutine, covered exclusively by eglandular hyaline hairs). Furthermore, *T. chrysophylla* can be differentiated from almost all the species of T. subg. Austrotradescantia by its golden to light brown indumentum covering almost the entire plant. The only other species known to possess a similarly coloured indumentum are *T. cerinthoides* (*T. crassula* group; Pellegrini 2015, 2016) and *T. tucumanensis* (*T. tenella* species complex; Pellegrini 2017). *Tradescantia chrysophylla* can be easily differentiated by its indefinite habit base (*vs.* definite in *T. cerinthoides*), prostrate stems (*vs.* ascending to erect), saccate cincinni bracts (*vs.* non-saccate), pistil the same length as the stamens (*vs.* longer than the stamens), petals always white (*vs.* ranging from white to pink to lilac), seed not cleft towards the embryotega (*vs.* cleft) and hilum ½ the length of the seed (*vs.* longer than ½ the length). *Tradescantia chrysophylla* and *T. tucumanensis* can be confused due to their similar habit, light brown to golden indumentum and asymmetrical cincinni bracts. Nonetheless, both species are easily differentiated by their non-overlapping distributions (endemic to Brazil in *T. chrysophylla vs.* restricted to the Tucumano-Boliviano Forest in *T. tucumanensis*), leaf morphology (leaves sessile, succulent, with inconspicuous secondary veins in *T. chrysophylla vs.* subpetiolate, membranous to chartaceous, with impressed secondary veins in *T. tucumanensis*). Finally, *T. chrysophylla* is easily identified in dried specimens, since it becomes peculiarly dark brown to black, added to the large epidermal domes in the leaf-blades.

Hassemer et al. (2017) describe *T. serrana* as a new species, known from a sole collection and endemic to the state of Santa Catarina. They compare their new species with *T. umbraculifera*, with which it bears very little resemblance. They also compare *T. serrana* with *T. chrysophylla*, differing both species based on the shape of their leaves, concentration of hairs on both sides of the blades, the presence of dorsal keels in the sepals, the posture of the petals (i.e. flat *vs.* plicate) and the relative length between the androecium and the pistil. Nonetheless, the sepals’ midvein was misinterpreted by the authors as representing dorsal keels (which, for instance, can be easily observed in *T. fluminensis*; Fig. 6B), the repandous petals as being plicate (which is only known to occur in *T. atlantica* and *T. fluminensis*; Fig. 6D–F), but they failed to realise that the relative length between the stamens and pistil used by me in my MSc thesis (Pellegrini 2015), is actually approximate and that in the *T. crassula* group, the pistil is considerably longer than the stamens, as opposed to the approximately equal length in the *T. fluminensis* group. *Tradescantia chrysophylla* and *T. serrana* share the sessile and succulent leaves with inconspicuous secondary veins, blades velutine to hispid with light brown to golden hairs, unequal to strongly unequal cincinni bracts and pedicels and sepals glandular-pubescent or with a mixture of glandular and eglandular hairs. *Tradescantia serrana* undoubtedly represents nothing more than a synonym of *T. chrysophylla* and ishere treated as such.

**Figure 13.**
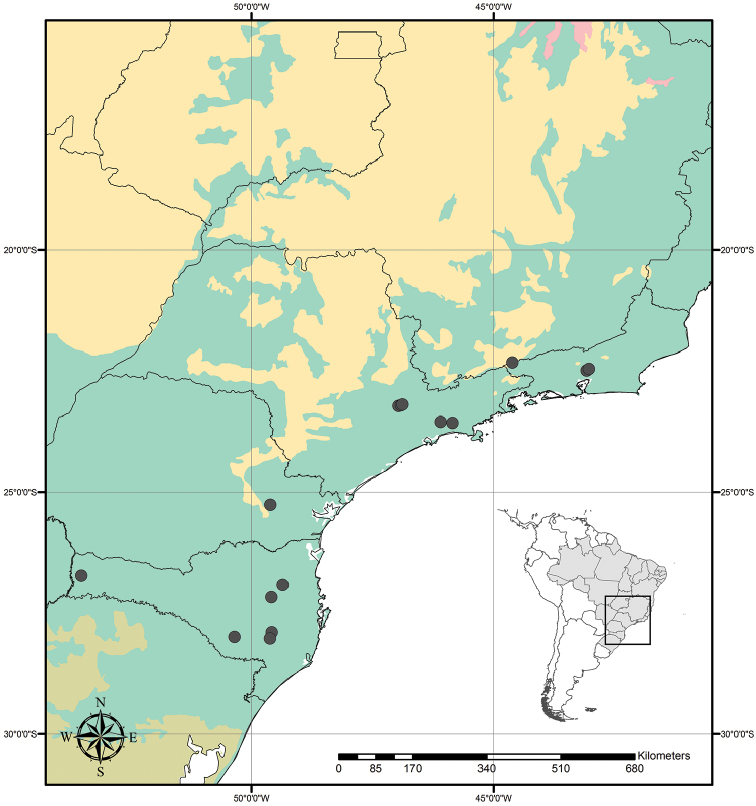
Distribution of *Tradescantia chrysophylla* M.Pell. Orange– Cerrado; Red– Caatinga; Olive-green– Pampa; Dark green– Atlantic Forest.

#### 4. Tradescantia crassula

Taxon classificationPlantaeORDOFAMILIA

Link & Otto, Icon. Pl. Rar. [Link & Otto] 2: 13, pl. 7. 1828.

Figs 14 , 15

Tropitria crassula (Link & Otto) Raf., Fl. Tell. 3: 68. 1837. Lectotype (designated by Funez et al. 2016). [Illustration] Original parchment plate of “*Icones plantarum rariorum Horti Regii Botanici Berolinensis cum descriptionibus et colendi ratione*” in the Botanischer Garten und Botanisches Museum Berlin-Dahlem and later published in Link & Otto, Icon. Pl. Rar. 2: 13, pl. 7. 1828.
Tradescantia schwirkowskiana Funez et al., Phytotaxa 272 (1): 64. 2016. Holotype. BRAZIL. Santa Catarina: São Bento do Sul, borda da ferrovia às margens do Rio Banhados, fl., fr., 16 Nov 2015, L.A. Funez & P. Schwirkowski 5037 (FURB no. 50791!; isotypes: C n.v., HURB no. 20200!).
Tradescantia crassipes Graham, Edinburgh New Philos. J. Jan.–March: 388. 1829, nom. nud.

##### Description.

*Herbs* ca. 10–50 cm tall, with a definite base, terrestrial, rupicolous or epiphytes. *Stems* erect, succulent, rarely to densely branched at the base, sometimes branching at the upper half; internodes (1.3–)3.2–5.4(–9.2) cm long at base, distally shorter, medium to dark green, glabrous, sometimes with a leaf-opposed longitudinal line of short, uniseriate, light brown to hyaline hairs in the terminal portion of the stems. *Leaves* distichously or spirally-alternate, sessile; ptyxis convolute; sheaths 0.6–2 cm long, light green, sometimes with green striations, glabrous, margin ciliate to setose, hairs hyaline; blades 4.4–12.8 × 1.4–2.8 cm, elliptic to broadly elliptic to ovate to broadly ovate to obovate, rarely lanceolate, falcate to complicate, succulent, glabrous on both sides, adaxially glossy light-green to green, sometimes glaucous, abaxially slightly lighter, turning olive-green to greyish-green to brown when dry, obtuse to truncate, rarely cuneate, margin green, glabrous or minutely ciliolate, slightly revolute, apex acute to obtuse, rarely acuminate; midvein conspicuous to inconspicuous, adaxially impressed to inconspicuous, secondary veins inconspicuous on both sides, sometimes adaxially conspicuous. *Synflorescences* terminal or axillar in the distal portion of the stems, composed of a solitary main florescence, 1 per leaf axis. *Inflorescences (main florescences)* consisting of a pedunculate double-cincinni fused back to back, sometimes reduced to a solitary cincinnus in axillary inflorescences; peduncles (0.3–)1.4–3.6(–7.3) cm long, green, the axillary ones sessile, glabrous, sometimes with a leaf-opposed longitudinal line of short, uniseriate, light brown to hyaline hairs; peduncle bracts absent; supernumerary bracts absent; cincinni bracts 1.2–3.9(–6) × 0.7–2.1 cm, leaf-like, rarely unequal or reduced in some axillary inflorescences, broadly ovate to ovate, leaf-like, glabrous, adaxially light to medium to dark green, abaxially light to medium green, base cordate to obtuse, not saccate, margin glabrous to minutely ciliolate, sparsely setose at base, slightly revolute, apex acute; main florescence 8–28-flowered. *Flowers* 0.8–1.2 cm diam., pedicels 0.7–1.5 cm long, green to vinaceous, glabrous, rarely sparsely glandular-pubescent; floral buds broadly ovoid; sepals 4.6–7.5 × 2.7–4.4 mm, dorsally keeled, green, rarely vinaceous, setose, with long hyaline hairs along the keel; petals 6–7.3 × 4.7–5.2 mm, flat, white; filaments 5.1–6.6 mm long, anthers 0.6–0.8 × 1.1–1.3 mm; ovary 1.7–1.9 × 1.5–1.7 cm, style 4.2–5 cm long; pistil longer than the stamens. *Capsules* 3.6–4.2 × 2.1–2.7 mm. *Seeds* 1.2–1.8 × 1.1–1.6 mm, testa grey to greyish-brown, cleft towards the embryotega, costate; hilum longer than ½ the length of the seed.

##### Specimens seen.

**ARGENTINA. Misiones**: Cainguás, pequeño campo a la entrada del Salto Golondrina, sobre Arroyo Guiray, fl., fr., 8 Nov 2000, M.E. Múlgura de Romero et al. 2470 (CORD, CTES, SI); General Manuel Belgrano, ruta nacional 101, 8 km de Bernardo de Irigoyen hacia San Antonio, Salto Andrecito, fl., 15 Oct 1996, O. Morrone et al. 1393 (CORD, CTES, SI). **BRAZIL. Without province**: Brasil australis, fl., s.dat., s.leg. s.n. (L barcode L1432960); cultivé en Hort. Bot. Parisiense, fl., 1834, Houller s.n. (P barcode P02173930); fl., Apr 1829, R. Graham s.n. (K, P barcode P02173931). **Minas Gerais**: Araponga, Salazar de Cima, fl., 16 Sep 2003, A.F. Carvalho 391 (IPA); Extrema, trilha para a Pedra das Flores, fl., 24 Oct 2009, G.H. Shimizu 226 (RB, UEC); Jesuânia, fl., fr., Nov 1919, D. Vincent s.n. (L barcode L1432976). **Paraná**: Adrianópolis, rio Itapeva, fl., 19 Feb 1981, G. Hatschbach 43622a (ASU, INPA, MBM, MO); Bocaiúva do Sul, arredores de Bocaiúva do Sul, fl., 5 Dec 1978, G. Hatschbach 41924 (MBM, US); Campo Largo, fl., 15 Dec 1967, L.T. Dombrowski 2744 (MBM, P, US); Carambeí, Catanduva de Fora, fl., 15 Oct 2013, M.E. Engels 1776 (MBM); estrada para o rio São João, Alto Carambeí, fl., 2 Nov 2013, E.D. Lozano & M.E. Engels 1716 (FLOR, HCF, MBM); Caxias do Sul, Ana Rech, Faxinal, fl., fr., 8 Nov 2002, L. Scur 961 (FUEL, HUCS); Cerro Azul, cabeceira do Ribeirão do Tigre, fl., 18 Jul 1984, G. Hatschbach 48100 (MBM); fl., 24 Apr 1987, G. Hatschbach et al. 51236 (MBM, US); Curitiba, estrada para São Paulo, km 14, fl., 6 Nov 1960, E.A. Moreira 70 (MBM, US); Jardim Botânico de Curitiba, fl., 24 Sep 2014, M.O.O. Pellegrini et al. 408 (RB); Parque Municipal Capão da Embuia, fl., fr., Nov 1974, L.T. Dombrowski 5392 (K, MBM); fl., 8 Nov 2001, M. Borgo 828 (UPCB); fl., 25 Sep 2014, M.O.O. Pellegrini et al. 411 (RB); Pinheirinho, fl., Nov 1966, L.T. Drombowski et al. 2067 (MBM, US); Guarapuava, Guará, fl., 5 Dec 1968, G. Hatschbach 20490 (MBM); Ipiranga, 29 km de Ipiranga, fl., fr., Nov 1984, J. Mattos & N. Silveira 26566 (HAS); Lapa, rio Passa Dois, fl., 5 Oct 1958, G. Hatschbach 5139 (MBM, UPCB); fl., 1 Oct 1969, G. Hatschbach 22307 (K, MBM, MO, US); Palmas, fl., 20 Oct 1990, G. Hatschbach 54807 (HUEFS, MBM, US); rodovia BR-449, km 5–10, fl., fr., 16 Nov 1998, G. Hatschbach 68709 UPCB); Piraquara, Fazenda Experimental de Agronomia, 13 Oct 1969, N. Imaguire 1258 (MBM); 13 Nov 1973, N. Imaguire 3399 (MBM); Quatro Barras, Morro Anhangava, 25 Sep 1998, A. Dunaiski Jr. & W. Amaral 761 (MBM); Rio Branco do Sul, Serra do Caetê, 10 Jan 1978, G. Hatschbach 40703 (MBM); São José dos Pinhais, Aeroporto Afonso Pena, 26 Nov 2009, R. Ristow et al. 231 (MBM); Caminho do Vinho, área de plantação de agrião, fl., fr., 25 Sep 2014, M.O.O. Pellegrini et al. 414 (RB); São Mateus do Sul, Fazenda do Durgo, 19 Sep 1986, W.S. Souza et al. 334 (MBM, UEC, UPCB); Sapopema, fl., fr., 27 Sep 1997, C. Medri et al. 436 (FUEL); fl., fr., 31 Oct 1998, C. Medri et al. 731 (FUEL); Tibagí, fl., 9 Apr 1999, D.C. Rocha s.n. (BOTU no. 22991); Tunas do Paraná, Parque Estadual de Campinhos, 21 Oct 2008, G. Weiss 86 (MBM). **Rio Grande do Sul**: Arroio dos Ratos, Fazenda Faxinal, ca. 15 km SW da cidade, fl., Sep 1982, N. Silveira 400 (HAS); fl., 8 Oct 1982, K. Hagelund 14026 (ICN); Augusto Pena, fl., 22 Oct 1953, P.J. Pivetta 964 (HRCB, PACA); Bocaina do Sul, Pessegueiros, fl., 8 Nov 2010, G.A. Dettke 477 (ICN); Caçapava do Sul, Guaritas, estrada para as Minas do Camaquã, fl., 9 Oct 2002, J.R. Stehmann et al. 3189 (BHCB); Cachoeira do Sul, rodovia Porto Alegre-Uruguaiana, km 145, fl., Oct 1984, N. Silveira & J. Mattos 7042 (HAS); Canguçu, fl., Nov 1986, N. Silveira 8967 (HAS); Caxias do Sul, Vila Oliva, fl., fr., 5 Jan 1946, B. Rambo 31069 (PACA); Esmeralda, fl., Nov 1982, L.A. Cestaro s.n. (HAS no. 28715); Farroupilha, fl., 25 Nov 1956, O.R. Camargo 1024 (PACA); Parque dos Pinheiros, fl., Nov 1978, L. Aguiar s.n. (Has no. 8926); Garibaldi, fl., 29 Oct 1957, O.R. Camargo 2304 (PACA); Guaíba, Fazenda Maximiano, BR-116, km 308, fl., 20 Aug 2006, L.F. Lima s.n. (ICN no. 151445); Ijuí, Dr. Pestana, fl., 5 Nov 1953, J. Pivetta 963 (PACA); Lages, UAP2/UAL2, fl., 11 Nov 2013, E.D. Santos et al. 319 (FLOR); UAP5/UAL2, fl., 10 Nov 2013, E.D. Santos et al. 265 (FLOR); Lavras do Sul, capoeira 1 km N de Lavras do Sul, fl., Oct 1971, B.E. Irgang & J.C. Lindeman 1416 (EFC, HAS); Montenegro, Campestre, fl., fr., 18 Oct 1950, A. Sehnem s.n. (PACA no. 51067); Pareci, fl., 1944, B. Rambo 26664 (PACA); Morro Reuter, fl., 17 Nov 1971, R.A. Wasum 204 (HUCS, PACA); Nova Petrópolis, fl., 15 Dec 1974, K. Hagelund 8754 (ICN); Palmares do Sul, Fazenda do Martin, fl., 24 Sep 2012, E. Valduga 442 (FLOR, FUEL, HUCS); Porto Alegre, Barra do Ribeiro, fl., 14 Nov 1948, B. Rambo 38309 (PACA); fl., 2 Nov 1957, O.R. Camargo 2453 (PACA); Morro São Pedro, Econsciência Espaço de Conservação, fl., 18 Sep 2005, R. Setubal & G. Seger 344 (ICN); fl., 12 Oct 2006, R. Setubal et al. 858 (ICN); Vila Manresa, fl., 22 Oct 1955, B. Rambo 57077 (HBR, PACA); Rio Grande, Estação Ecológica do Taim, fl., Sep 1986, J.L. Aechter 2216 (HAS); Santana da Boa Vista, Arroio Vargas, fl., 23 Sep 1998, C. Mondin 1483 (PACA); São Francisco de Paula, Cambara, fl., fr., Feb 1948, B. Rambo 36589 (PACA); 29 km em direção à Bom Jesus, fl., fr., Nov 1986, M. Neves 772 (HAS); São Leopoldo, fl., 1941, S.J. Orth s.n. (PACA no. 34066); fl., 6 Nov 1941, B. Rambo 10725 (PACA); fl., 11 Sep 1946, Ritter s.n. (PACA no. 35382); São Marcos, rodovia Porto Alegre-Vacaria, km 138, fl., Nov 1978, J. Mattos 20353 (HAS); São Martinho da Serra, fl., 12 Oct 2011, G.A. Dettke & J. Durigon 977 (ICN); Sapucaia do Sul, fl., 17 Oct 1949, B. Rambo 43939 (PACA); Torres, fl., 19 Jan 1975, K. Hagelund 8716 (ICN); Morro da Itapeva, próximo à praia, fl., Jun 1984, N. Silveira et al. 1451 (HAS); Vale Vêneto, fl., 9 Sep 1961, P.J. Pivetta s.n. (HRCB no. 46373); Viamão, Morro Grande, fl., 15 Sep 1997, S.C. Müller 2 (ICN); Parque Estadual de Itapuã, Morro do Araçá, fl., 1 Oct 2003, M. Pinheiro 425 (ICN); fl., 4 Oct 2002, M. Pinheiro 552 (ICN). **Santa Catarina**: Araranguá, Sombrio, fl., 19 Sep 1945, R. Reitz s.n. (PACA no. 31883); Bom Retiro, between Fazenda Santo Antônio and Fazenda Campo dos Padres, fl., fr., 24 Jan 1957, L.B. Smith & R. Reitz 10392 (HBR, R); estrada para Urubici, fl., fr., 3 Dec 2014, M.O.O. Pellegrini et al. 432 (RB); Campo Belo do Sul, Fazenda Gateados, fr., 15 Jul 2008, M. Verdi et al. 2028 (FURB, RB); Coronel Domingos Soares, Fazenda Nossa Senhora de Fátima, fl., fr., 24 Nov 2013, S. Campestrini et al. 688 (FLOR); Curitibanos, Capão, fl., 28 Oct 1963, R.M. Klein 4047 (HBR); Imbituba, Praia Boa Esperança, fl., 21 Aug 1992, G. Hatschbach & M. Hatschbach 57897 (ASU, FLOR, MBM, US); Jaborá, Linha Pinhal, fl., 19 Nov 2016, H.F. Uller 926 (FURB); Laguna, fl., 23 Jan 1982, A. Krapovickas et al. 37740 (CTES, MBM); margem leste da BR-101, ca. 5 km N da cidade, fl., 2 Oct 1993, D.B. Falkenberg 6243 (FLOR); Palmares, Campos Novos, Pinhal, fl., 28 Oct 1963, R.M. Klein 4104 (HBR); fl., 29 Oct 1963, R.M. Klein 4205 (HBR); São Bento do Sul, Floresta Ombrófila Mista, fl., fr., 31 Dec 2013, P. Schwirkowski 197 (FURB, MBM); Serra Alta, Vila Pilz, estrada de Ferro, fl., 30 Oct 2016, P. Schwirkowski 2003 (FURB, MBM); São Joaquim, fl., Feb 1954, J. Mattos 1037 (HAS); Bom Jardim da Serra, Fazenda da Laranja, fl., 10 Dec 1958, R. Reitz & R.M. Klein 7708 (HBR); fl., fr., 13 Dec 1958, R. Reitz & R.M. Klein 7882 (HBR); Fazenda do Sr. Antônio Carvalho, fl., Jan 1956, J. Mattos 3254 (HAS); Invernadinha, fl., Jan 1956, J. Mattos 2816 (HAS); Taperinha, Portinho, fl., Jan 1956, J. Mattos 3322 (HAS); Tipitinga, Campos Novos, fl., fr., 29 Oct 1963, R. Klein 4249 (HBR); Urubici, Morro da Igreja, 9 Nov 2001, G. Hatschbach et al. 72486 (MBM); fl., 9 Nov 2010, G.A. Dettke 475 (ICN). **São Paulo**: s.loc., fl., 1827, W.J. Burchell 4730 (K, L); Bonsucesso de Itararé, Fazenda Ibiti (Ripasa), beira da estrada Itararé-Bonsucesso, fl., fr., 30 Oct 1993, V.C. Souza et al. 4531 (ESA, RB); Cunha, estrada Cunha-Paraty, fl., fr., 8 Nov 1976, P.E. Gibbs 3431 (MBM, UEC); Jundiaí, Serra do Japi, fl., fr., 25 May 1994, J. Semir et al. 31648 (UEC); Trilha do Mirante, fl., fr., 18 Jul 1995, R. Mello-Silva et al. 1074 (SPF); Serra do Jundiaí, ca. 10km SW de Jundiaí, fl., fr., 8 Oct 1976, H.F. Leitão Filho et al. 3172 (UEC); sentido bairro Eloy Chaves, próximo à represa do DAE, fl., 23 Jan 1998, E.R. Pansarin 136 (SP, UEC); Trilha para a antena da TV Cultura, fl., 18 Aug 2015, T.L. Vieira et al. 266 (SP); São Paulo, Cidade Jardim, fl., fr., 11 Mar 1932, W. Hoehne s.n. (IPA 69219, SPF 17149); Horto da Faculdade de Farmácia, fl., fr., Aug 1938, W. Hoehne s.n. (K barcode K001248113, SP no. 10555). **URUGAUY. Without province**: Banda Oriental del Uruguay, fl., 1816–1821, A. Saint-Hilaire C2 1881 (P). **Cerro Largo**: Sierra de los Ríos, fl., fr., 16 Oct 1910, M.B. Berro 5958 (K).

##### Distribution and habitat.

*Tradescantia crassula* occurs in Argentina, Brazil (in the states of Minas Gerais, São Paulo, Paraná, Santa Catarina and Rio Grande do Sul) and Uruguay; in the Atlantic Forest, Cerrado and Pampa domains (Fig. 15). It is commonly found growing in rocky outcrops, grasslands and open areas, under full sunlight, as rupicolous or terrestrial. It is also found on roadsides and within the understorey of open forests, as terrestrial or, more rarely, as an epiphyte (Pellegrini et al. 2017).

##### Phenology.

It was found in bloom and fruit throughout the year but peaking during the rainy season and being less commonly in bloom during the dry season.

##### Etymology.

The epithet “*crassula*” makes reference to the extremely succulent vegetative parts characteristic of this species.

##### Conservation status.

As stated by Pellegrini et al. (2017), *T. crassula* possesses a wide EOO (here expanded to ca. 627,243.259 km^2^), thus following the IUCN recommendations (IUCN 2001), it should be considered Least Concern (LC).

##### Comments.

**Figure 14.**
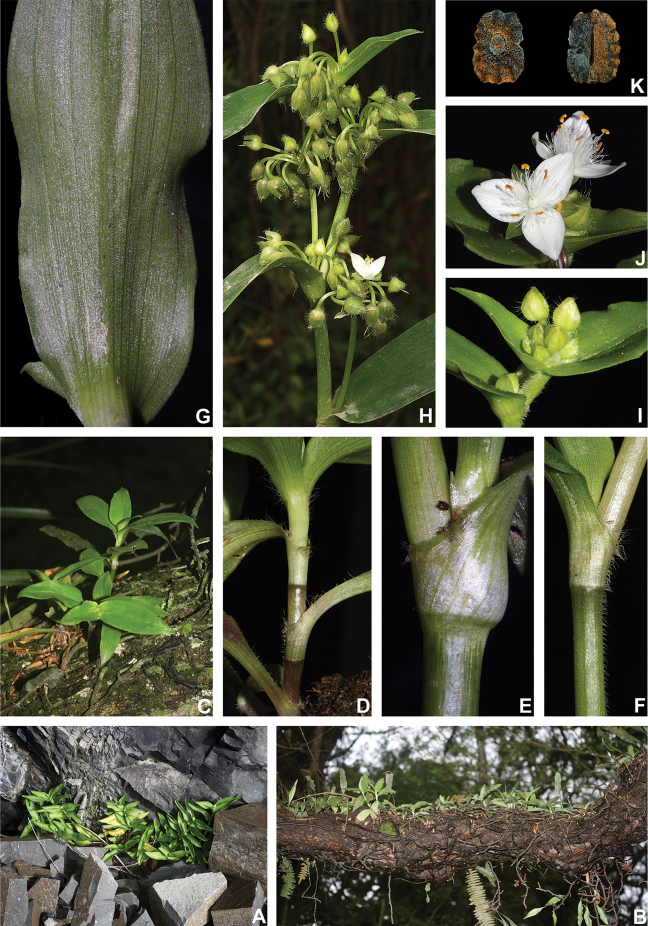
*Tradescantia crassula* Link & Otto. **A** habit, showing the erect stems and distichously-alternate leaves with conduplicate blades of a rupicolous specimen **B** habit, showing epiphytic subpopulation **C** detail of an epiphytic individual, showing the spirally-alternate leaves **D–F** stems and leaf-sheaths **D** vinaceous internodes of a young epiphytic specimen **E** medium green internode with a leaf-opposed line of uniseriate hairs **F** medium green internode, showing a leaf-opposed line of uniseriate hairs **G** detail of the leaf-blade, showing the glabrous surface **H** synflorescence **I** detail of the synflorescence, showing an axillary inflorescence composed of solitary cincinnus and a terminal with the typical double-cincinni with non-saccate cincinni bracts **J** flowers in front and side view, showing the setose sepals with hairs restricted to the dorsal keels and pistil longer than the stamens **K** dorsal and ventral views of the seed, showing the costate testa cleft towards the embryotega and the hilum longer than ½ the length of the seed. **H** by G.A. Dettke, remaining photos by M.O.O. Pellegrini.

*Tradescantia crassula* is very plastic in plant stature, leaf shape and flower size. Few vegetative characters were observed to be constant in the *T. crassula* group and thus are of little taxonomic relevance. Nonetheless, all studied individuals of *T. crassula* always present glabrous leaves (Fig. 14G), sepals with long hairs along the keel (Fig. 14H–J) and white petals (Fig. 14J). Added to that, most of the known variation can be related to ecological conditions. Furthermore, species in this subgenus are morphologically variable and, when kept in cultivation or growing in shaded areas, they can change their vegetative morphology quite drastically, changing between the different morphs (more or less succulent plants, smaller or taller plants, plants with different leaf shapes). Thus, I agree with Pellegrini et al. (2017) in treating *T. schwirkowskiana* as a mere synonym of *T. crassula*. Detailed comments on the morphological similarities between *T. crassula* and closely related species and the species morphological variation are presented by Pellegrini et al. (2017), while Pellegrini (2017) discusses its systematic affinities.

**Figure 15.**
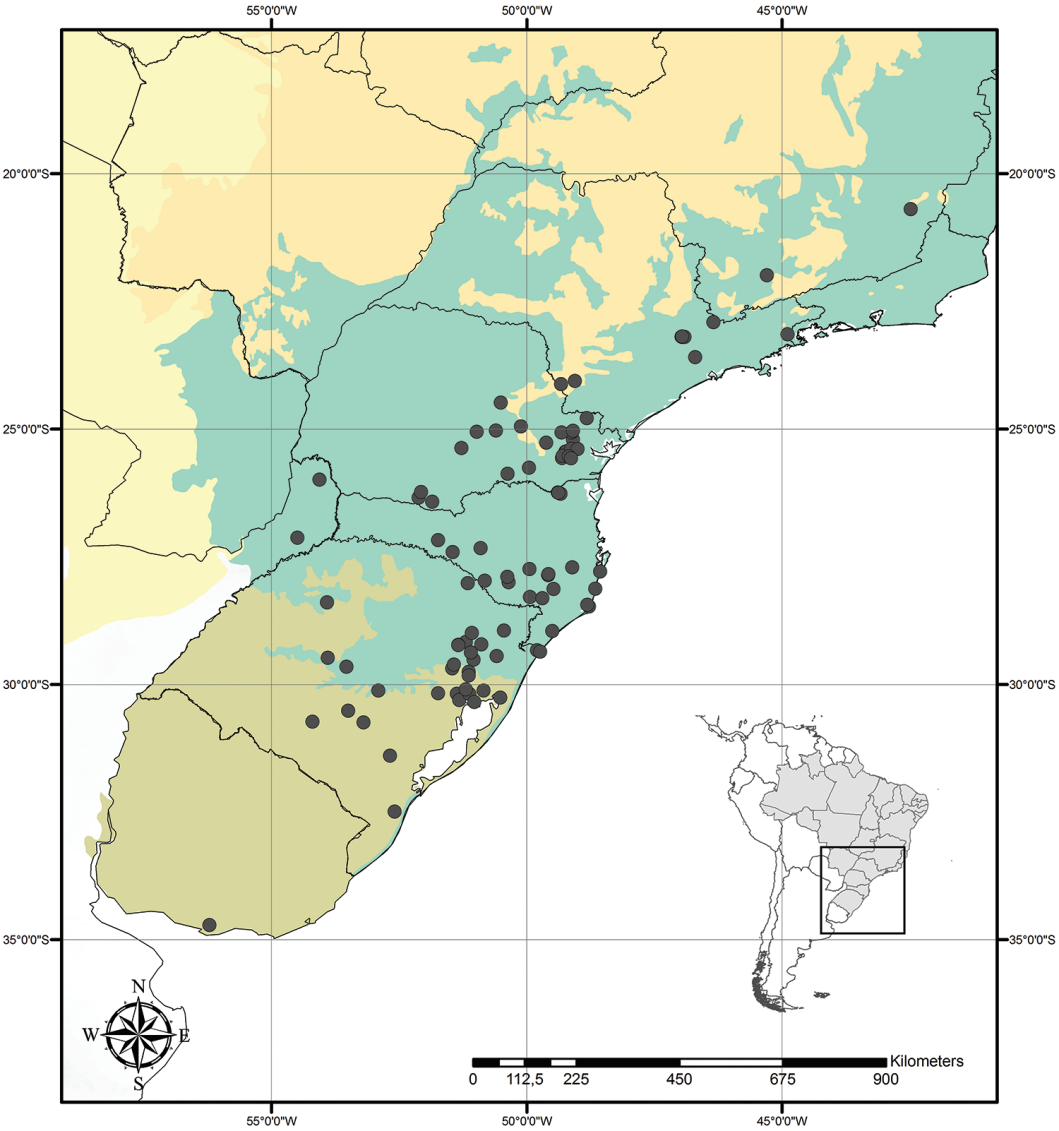
Distribution of *Tradescantia crassula* Link & Otto. Orange– Cerrado; Yellow– Chaco and Pantanal; Olive-green– Pampa; Dark green– Atlantic Forest.

#### 5. Tradescantia cymbispatha

Taxon classificationPlantaeORDOFAMILIA

C.B.Clarke in De Candolle & De Candolle, Monogr. Phan. 3: 296. 1881.

Figs 16 , 17

Cymbispatha geniculata Pichon, Notul. Syst. (Paris) 12: 224. 1946.
Tradescantia cymbispatha var. cymbispatha C.B.Clarke, in De Candolle & De Candolle Monogr. Phan. 3: 296. 1881.
Tradescantia geniculata Vell., Fl. Flumin.: 140. 1829, nom. illeg. non T. geniculata Jacq., Select. Stirp. Amer. Hist.: 94, t. 64. 1763. Lectotype (designated by Pellegrini et al. 2016). [illustration] Original parchment plate of “Flora fluminensis” in the Manuscript Section of the Biblioteca Nacional of Rio de Janeiro [cat. no.: mss1198652_157] and later published in Vellozo, Fl. Flumin. Icones 3: t. 153. 1831. Epitype (designated by Pellegrini *et al.* 2015). BRAZIL. Rio de Janeiro: Petrópolis, bairro Castelânea, fl., 8 Aug 2010, M.O.O. Pellegrini 17 (RB!; isoepitype: RFA!).
Tradescantia mundula var. scabrida Seub., in Martius Fl. Bras. 3(1): 249. 1855. Lectotype (designated by Pellegrini et al. 2015). BRAZIL. s.loc., fl., s.dat., F. Sellow 3852 (B barcode B 10 0247278!).

##### Description.

*Herbs* ca. 5–30 cm tall, with an indefinite base, terrestrial, rupicolous or epiphytes. *Stems* prostrate with ascending apex, delicate to slightly succulent, little to densely branched; internodes 0.8–6.4 cm long at base, distally shorter, medium to dark green or reddish-purple to vinaceous, strigose, hairs hyaline. *Leaves* distichously-alternate, sessile; ptyxis involute; sheaths 0.4–0.7 cm long, green to green with vinaceous striations or vinaceous, strigose, margin setose, hairs light brown; blades elliptic to broadly elliptic or broadly ovate, 1.2–4.6 × 0.6–2.2 cm, flat, succulent, strigose on both sides, adaxially dark to medium bluish-green, turning olive-green to medium brown when dry, abaxially light to medium green or vinaceous, turning tan to light brown when dry, base cordate to obtuse, margin green to vinaceous, ciliolate, slightly revolute, apex acute; midvein conspicuous, adaxially impressed, secondary veins inconspicuous, adaxially inconspicuous, abaxially inconspicuous, becoming more evident on both sides when dry. *Synflorescences* terminal or axillar in the distal portion of the stems, composed of a solitary main florescence, 1 per leaf axis. *Inflorescences (main florescences)* consisting of a pedunculate double-cincinni fused back to back; peduncles 0.6–4.3 cm long, medium to dark green or reddish-purple to vinaceous, strigose; cincinni bracts similar to each other, broadly elliptic to broadly ovate, 1–3.5 × 0.6–1.6 cm, leaf-like, strigose on both sides, adaxially dark to medium bluish-green, abaxially light to medium green or vinaceous, base cordate, saccate, margin ciliolate, slightly revolute, apex acute; double cincinni 6–10-flowered. *Flowers* 0.9–1.5 cm diam., pedicels 0.9–1.6 cm long, vinaceous, velutine, sometimes with some odd glandular hairs, hairs hyaline; floral buds broadly ovoid; sepals 6–6.5 × 2–2.6 mm, without keels, vinaceous, rarely green, velutine, hairs hyaline; petals 0.5–0.7 × 0.3–0.5 cm, flat, white, sometimes pink to lilac; filaments 4.1–4.6 mm long, anthers 0.8–1 × 1–1.3 mm; ovary 0.9–1.2 × 0.6–1 cm, style 2.6–3.2 cm long; pistil the same length as the stamens. *Capsules* 2.9–3.8 × 1.8–2.2 mm. *Seeds* 1.4–1.8 × 1.1–1.5 mm, testa grey to greyish-brown, not cleft towards the embryotega, costate; hilum ½ the length of the seed.

##### Specimens seen.

**ARGENTINA. Buenos Aires**: Campana, Reserva Natural Estricta Otamendi, fl., 1 Nov 2004, S. Torre Robles et al. 2048 (LP); General Pueyrredón, Reserva Integral Laguna De Los Padres, fl., 29 Oct 2004, S. Torres Robles et al. 2202 (LP); Punta Indio, Reserva De La Biosfera Parque Costero Del Sur, fl., 12 Oct 2002, S. Torres Robles 861 (LP). **Corrientes**: Concepción, Carambola, fl., 17 Dec 1974, A.E. Burkart 3782 (CORD, US); Mercedes, Paso Picada, orilla Laguna Iberá, frente a Carlos Pellegrini, fl., 22 Sep 1973, A. Schinini 7487 (CORD, CTES, SI); San Cosme, Ensenada Grande, ruta 12, fl., fr., 6 Oct 1971, A. Krapovickas et al. 20030 (CORD, CTES). **Misiones**: Apostóles, Escola Agrotécnica, fl., fr., Oct 1977, A.L. Cabrera et al. 28542 (CORD, CTES, SI); Cainguás, Salto Tabay, fl., 16 Oct 1947, S.A. Pierotti 6606 (CTES); fl., 12 Oct 1975, E.M. Zardini et al. 789a (LP, MO); Profundidad, fl., 19 Sep 1974, A. Krapovickas et al. 25689 (CTES); Guraní, Arroyo El Paraíso y ruta 2, fl., 23 Sep 1993, M. Rodriguez et al. 705 (CTES, K, MNES); fl., 23 Sep 1993, M. Rodriguez et al. 823 (CTES, MNES); 3 km S de Francán, fl., 27 Sep 2007, A. Schinini et al. 36874 (CTES, HUEFS); San Vicente, Cuartel Rio Victoria, campo anexo INTA, fl., 24 Aug 1995, M.E. Torri et al. 89 (CTES); Predio Guarani, picada hacia el Arroyo Paraiso, cruzando el Arroyo Itapyru, fl., 19 Sep 1995, S.G. Tressens et al. 5384 (CTES, FUEL); fl., 15 Oct 1997, S.G. Tressens et al. 5940 (CORD, CTES); parcela CIFOR, fl., 31 Aug 1999, S.G. Tressens et al. 6332 (CTES); Isla Martín García, fl., 23 Aug 1996, J. Hurrell & M. Belgrano 3131 (LP); Iguazú, Puerto Iguazú, fl., 4 Oct 1910, F.M. Rodriguez 483 (CORD, LP); fl., 2 Oct 1947, S.A. Pierotti 6569 (CTES, K, LIL); Parque Nacional Iguazú, Sendero Macuco, fl., 14 Oct 1993, S.G. Tressens et al. 4507 (CTES); Sendero Jacaratia, fl., 9 Aug 1995, R. Vanni et al. 3411 (CTES); Libertador General San Martín, Salto Encantado, 10 km NE de Aristóbulo del Valle, fl., 31 Jul 1987, R. Vanni et al. 920 (CORD, CTES). **Santa Fe**: Rosario, fl., 1 Sep 1987, Franceschi & Fernández 1216 (CTES). **BRAZIL. Without province**: s.loc., st., s.dat., Glaziou 36 (P); fl., s.dat., F. Sellow s.n. (B barcode B100247277). **Paraná**: Araucária, Campina dos Martins, fl., 17 Sep 1997, Y. Alquini s.n. (UPCB no. 30925); Coronel Vivida, fl., 27 Aug 1975, R. Kummrow & W. Golte 1926 (K, MBM); Curitiba, Centro Politécnico, fl., 28 Sep 1985, L.A. Acra s.n. (UPCB no. 14612); Parque Barigui, fl., fr., 19 Nov 1973, R. Kummrow 116 (K, MBM); Foz do Iguaçú, Iguaçú National Park, fl., 22 Aug 1985, G. Hatschbach & A.C. Cervi 49563 (MBM, US); Lapa, rodovia BR-476, km 210, Passa Dois/Pedra Alta, fl., fr., 23 Nov 2013, M.E. Engels D.T. Reynald (MBM, RB); Mangueirinha, fl., 30 Sep 1992, N.R. Marquesini s.n. (UPCB no. 21864); Morretes, Véu de Noiva, fl., 21 Aug 1985, J. Cordeiro & J.M. Silva 116 (CESJ, MBM); Palmeira, estrada Curitiba-Ponta Grossa, no trevo para Palmeira, BR-277, fl., fr., 2 Feb 1995, J.R. Stehmann & J. Semir 2143 (UEC); Fazenda Santa Rita, fl., fr., 18 Nov 1980, L.T. Dombrowski & G. Scherer 12217 (K, MBM); Piraquara, Rio de Meio, cachoeira, fl., fr., Sep 1977, P.F.B. Hertel 233 (FUEL); Rio Branco do Sul, near Curriola, fl., 14 Oct 1971, L.T. Dombrowski & Y.S. Kuniyoshi 3693 (MBM, US); Telêmaco Borba, Fazenda Monte Alegre, à beira do Ribeirão Varanal, st., 20 Apr 2005, T.I.N. Azevedo & S.I. Azevedo 117 (FUEL); fl., fr., 31 Oct 2006, T.I.N. Azevedo & S.I. Azevedo 494 (FUEL); Bota Fora Dois, fl., 19 Sep 2008, M. Kaehler 226 (UPCB); Poço Preto, foz do Rio das Antas, fl., 17 Sep 2008, M. Vallejos 3 (UPCB). **Rio de Janeiro**: s.loc., fl., Jun 1838, J. Miers 4039 (P); Petrópolis, Moinho Preto, próximo da Mosela, fl., 16 Aug 1989, L.C. Giordano et al. 771 (RB). **Rio Grande do Sul**: Bento Gonçalves, estrada para Guaporé, fl., 24 Aug 1998, R.A. Wasum et al. 12749 (HUCS, MBM, US); Bom Jesus, Fazenda do Cilho, fl., 18 Aug 2002, R.A. Wasum 1543 (HUCS, L, MBM, US); Serra do Rocinha, fl., 28 Feb 1946, B. Rambo 32428 (PACA); Caçapava do Sul, Pedra do Segredo, fr., 29 Dec 2004, T.B. Breier et al. 1250 (UEC); Canela, Vila Suzana, fl., Oct 1974, J. Thomé s.n. (HAS no. 874); fl., 1 Oct 1998, S. Diesel 1614 (US); Capão da Canoa, estrada Riozinho-Maquiné, ca. 22 km E de Riozinho, fl., 22 Nov 2005, M. Machado 594 (HUEFS); Capão do Leão, Horto Botânico Irmão Teodoro Luiz, fl., 9 Nov 1986, J.A. Jarenkow 501 (FLOR); Caxias do Sul, Vila Oliva, fl., fr., 3 Dec 1949, B. Rambo 44710 (K, LIL, PACA); Conceição, fl., 24 Oct 1987, R.A. Wasum 3373 (HUCS, US); Criúva, Ilhéus, fl., 30 Oct 1988 R.A. Wasum 4851 (HUCS, INPA, US); Derrubadas, Parque Estadual do Turvo, Tenente Portela, fl., Jan 1977, J. Mattos & N. Mattos 16433 (HAS); Esteio, fl., 8 Sep 1943, B. Rambo 1497 (PACA); Ilha do Laje, fl., Apr 1975, L. Aguiar & Z. Rosa s.n. (HAS no. 1429); Flores de Cunha, Otávio Rocha, fl., 10 Oct 1989, R.A. Wasum 6192 (HUCS, US); Galópolis, travessa Barata Góes, fl., 30 Sep 1984, G. Grazziotin 308 (US); Erechim, Chácara Linos Klein, fl., 23 Sep 1994, A. Butzke 7452 (US); Farroupilha, fl., 15 Nov 1956, O.R. Camargo 954 (PACA); Parque dos Pinheiros, fl., Sep 1978, L. Martau s.n. (HAS no. 8759); Gramado, Pousada Cabanas do Tio Müller, fl., fr., 11 Nov 2006, R. Marquete & E.V.S. Medeiros 4000 (RB); Montenegro, Kappesberg, fl., 1 Sep 1946, A. Sehnem s.n. (PACA no. 48435); fl., 11 Sep 1949, B. Rambo 43432 (PACA); Pareci Novo, st., 1944, E. Henz s.n. (PACA no. 27649); Nova Hamburgo, Ferrabraz, fl., 14 Oct 1936, B. Rambo 2785 (PACA); fl., fr., 2 Sep 1949, B. Rambo 43230 (PACA); Nova Petrópolis, Morro Reuter, fl., 23 Oct 1966, A. Sehnem 8924 (PACA); Passo Fundo, fl., 30 Oct 1971, J.C. Lindeman et al. s.n. (EFC, CTES no. 203179, ICN no. 8840); Porto Alegre, Itapu, no Morro da Grota, fl., Oct 1984, J. Mattos et al. 26110 (HAS); Morro Santana, fl., Aug 1987, N. Silveira 4840 (HAS); Rio Grande, Estação Ecológica do Taim, fl., 18 Sep 1986, J.L. Waechter 2206 (FLOR); Rolante, Arroio Chuvisqueiro, Cascata do Chuvisqueiro, fl., 5 Oct 1988, S. Diesel 69811 (US); Santo Ângelo, Granja Piratini, lagoa 7, fl., 4 Aug 1973, K. Hagelund 7053 (CORD, ICN); fl., 23 Sep 1976, K. Hagelund 10487 (CORD, ICN); Santa Maria, várzea do Rio Santa Maria, 45 km E de Don Pedrito, fl., 15 Oct 1971, J.C. Lindeman et al. s.n. (EFC, CTES no. 202945, ICN no. 8587); fl., 6 Oct 2011, G.A Dettke & J. Durigon 966 (ICN); Santiago, rodovia para Santa Maria, 26 km de Santiago, fl., Oct 1984, J. Mattos et al. 26126 (HAS); São Roque, estrada São Roque-Farroupilhas, fl., fr., 6 Oct 1985, I. Guerra 1166 (HUCS, INPA, US); Vacaria, perto do Rio Pelotas, fl., Sep 1977, J. Mattos & N. Mattos 17314 (HAS); Veranópolis, Estação Experimental Fitotécnica, fl., Oct 1983, J. Mattos 25172 (HAS); Vale do Rio das Antas, fl., Oct 1988, J. Mattos 31471 (HAS). **Santa Catarina**: Alfredo Wagner, Rua Anitápolis, fl., fr., 14 Aug 2009, M. Verdi et al. 2573 (FURB); Blumenau, Morro do Sapo, Parque Nacional da Serra do Itajaí, fl., 5 Oct 2013, A.L. Gasper et al. 3110 (FURB); Bom Retiro, Capão do Campo, fl., 25 Oct 1957, R. Reitz & R.M. Klein 5469 (HBR, L, NY, US); Campo dos Padres, fr., 22 Jan 1957, B. Rambo 60119 (PACA); Caçador, Ponte Alta do Norte, fl., fr., 24 Oct 1962, R. Reitz & R.M. Klein 13371 (HBR); Campo Novo, acesso alternativo ao Campo dos Padres, fl., fr., 12 Nov 2011, A.L. Gasper et al. 2939 (FURB); Faxinal dos Guedes, Parque Três Pinheiros, fl., 30 Sep 2015, A.A. Oliveira 2562 (FURB); Itapiranga, Tunas, fl., 31 Aug 1964, R.M. Klein 5708 (HBR); Lages, Passo do Socorro, fl., 31 Oct 1963, R.M. Klein 4353 (HBR); Major Vieira, Rio da Serra, fl., 27 Oct 2010, A. Korte & A. Kniess 4797 (FURB, HURB, RB); Papanduva, Serra do Espigão, fl., 24 Oct 1962, R. Reitz & R.M. Klein 13430 (HBR); Rio Rufino, fl., G.A. Dettke 483 (ICN); Rodeio, próximo a divisa Rodeio-Benedito Novo, fl., 18 Aug 2012, L.A. Funez 792 (FURB, HUCS); Bairro Ipiranga, fl., 10 Oct 2013, L.A. Funez 2277 (FURB); fl., 10 Oct 2013, L.A. Funez 2278 (FURB); São Joaquim, barra do Rio São Mateus, fl., fr., Jan 1957, J. Mattos 4801 (HAS); fl., Jan 1957, J. Mattos 4324 (HAS); Urubici, Serra do Corvo Branco, fl., Oct 1981, J. Mattos 22747 (HAS); fl., 9 Nov 2010, G.A. Dettke 472 (ICN); Véu da Noiva, Avencal, fl., fr., M.O.O. Pellegrini et al. 443 (RB). **São Paulo**: Itapecerica da Serra, fl., fr., Jun 1965, J. Mattos 11855 (SP); São José do Barreiro, Parque Nacional da Serra da Bocaina, fl., fr., 21 Oct 1984, R.A. Wasum 480 (HUCS, US); fl., 6 Oct 2011, F.M. Pinheiro et al. 102 (RB). **PARAGUAY. Without province**: s.loc., fl., Oct 1982, C.E.O. Kuntze s.n. (US barcode US00045775!); La Plata, fl., 1853, E. Palmer s.n. (US barcode US00045776). **Caaguazú**: Tajao, fl., 28 Aug 2002, F. González Parini 1185 (FCQ). **Central Department**: Areguá, Cerro Kõi, fl., Oct 1971, A. Schinini 4044 (CORD, CTES); Asunción, Trinidad, Jardin Botanico and Zoo, fl., Sep 1991, B. Pérez 1185 (US); Estero del Ypoa, S of Nueva Italia, fl., 21 Aug 1992, E.M. Zardini & P. Aquino 33098 (LP, MO, US); Tavarory, Rio Paraguay, Arroio Abai, fl., 3 Aug 1990, E.M. Zardini & C. Velasquéz 22425 (LP, MO, US); fl., 9 Sep 1990, E.M. Zardini 23288 (LP, MO, US). **Cordillera**: Tobatí, Cerros de Tobatí, fl., fr., 1900, E. Hassler 6093 (K, P); fl., 21 Jul 1987, A. Krapovickas 41887 (CORD, CTES); Cerro Ybitú Silla, 1 km S de Tobatí, fl., 23 Jul 1988, E.M. Zardini 6004 (FCQ, K, LP, MO, UPCB); Meseta Ybitú Silla, fl., 25 Aug 1988, E.M. Zardini 6708 (K, LP, MO); fl., 22 Sep 1988, E.M. Zardini 7383 (FCQ, K, MO); fl., 22 Sep 1988, E.M. Zardini 7422 (FCQ, K, LP, MO); San Bernardino, fl., Jun 1915, E. Hassler 236 (CORD); fl., s.dat., E. Hassler 384 (K); fl., s.dat., E. Hassler 496 (K); fl., fr., s.dat., E. Hassler 997 (K, P); Lago Ypacaraí, fl., s.dat., E. Hassler 3095 (K). **Itapúa**: Trinidad, 3.7 km de Trinidad camino a Jesus, fl., 7 Oct 1993, A. Krapovickas & C.L. Cristóbal 44503 (CORD, CTES). **Paraguarí**: Acahay, Cerro, fl., 6 May 1987, R. Degen 155 (CORD, CTES, FCQ); Macizo Acahay, fl., 21 Jul 1988, E.M. Zardini 5829 (K, LP, MO); fl., fr., 26 Jul 1988, E.M. Zardini 6082 (K, LP, MO); st., 21 Aug 1988, E.M. Zardini 6587 (K, LP, MO); fl., fr., 3 Sep 1988, E.M. Zardini & T. Florentin 6931 (K, LP, MO, PY); fl., fr., 21 Aug 1988, E.M. Zardini 6626 (K, LP, MO); fl., fr., 5 Sep 1988, E.M. Zardini & T. Florentin 7067 (K, LP, MO, PY); st., 26 Feb 1992, E.M. Zardini & P. Aquino 30682 (LP, MO, US); Atyrá, Cerro Zanja Jhú, 1 km E of road from Route 1 to Atyrá, 3 km before Atyrá, fl., fr., 30 Jul 1988, E.M. Zardini 6208 (K, LP, MO, PY); Cerro Palacios, fl., 30 Oct 1987, I. Basualdo et al. 1479 (FCQ); fl., 30 Oct 1987, E.M. Zardini & I. Basualdo 3745 (K, LP, MO); Paraguarí, San Thomas, fl., fr., 1885, E. Hassler 997 (K, P); Quiíndy, Parque Nacional Lago Ypoá, Valle Apuá, fl., 9 Nov 2000, F. Mereles et al. 8236 (FCQ); Ybycui National Park, Arroyo Minas, fl., 1 Oct 1985, A.H. Gentry 51885 (MO, US); fl., 15 Sep 1988, E.M. Zardini 7308 (K, LP, MO, PY); fl., 3 Jun 1989, A. Aguayo 230 (US); fl., 20 Aug 1991, E.M. Zardini & T. Telleria 28380 (LP, MO, US); Parque Nacional Iwykuí, fl., fr., 12 Oct 1979, E. Bordas 1099 (CORD, CTES). **San José**: Primavera, cerca del Río Capiracuai, fl., 25 Jun 1957, A. Woolston 829 (K, P, U). **URUGUAY. Without province**: Central Uruguay, fl., 1888, T. Morong 15 (US).

##### Distribution and habitat.

Argentina, Brazil (states of Rio de Janeiro, São Paulo, Paraná, Santa Catarina and Rio Grande do Sul), Paraguay and Uruguay; in the Atlantic Forest, Chaco and Pampa domains (Fig. 17). It can be found growing understorey in shaded and moist forests as terrestrial, rupicolous and sometimes as an epiphyte.

##### Phenology.

It was found in bloom throughout the year but peaking during the rainy season and being less commonly in bloom during the dry season. Fruit has been sporadically observed during the flowering period.

##### Etymology.

The epithet makes clear the great nomenclatural confusion created by Clarke’s misinterpretation of Vellozo’s plate. It means “boat-shaped bract”, a character present only in the Bolivian *T. praetermissa* M.Pell. and other members of T. subg. Campelia, on which Clarke partially based his description (Pellegrini et al. 2016; Pellegrini 2017).

##### Conservation status.

*Tradescantia cymbispatha* possesses a wide EOO (ca. 1,201,901.629 km^2^), being also cultivated throughout its distribution range as an ornamental plant. Thus, following the IUCN recommendations (IUCN 2001), it should be considered Least Concern (LC).

##### Nomenclatural notes.

*Tradescantia cymbispatha* was originally described as *T. geniculata* Vell. on *Florae fluminensis* (Vellozo 1829, 1831). However, since this name was a posterior homonym of *T. geniculata* Jacq., it was almost completely overlooked by most botanists until Clarke (1881) inadvertently proposed *T. cymbispatha* C.B.Clarke as a replacement name. These matters were only recently clarified by Pellegrini et al. (2015, 2016).

##### Comments.

**Figure 16.**
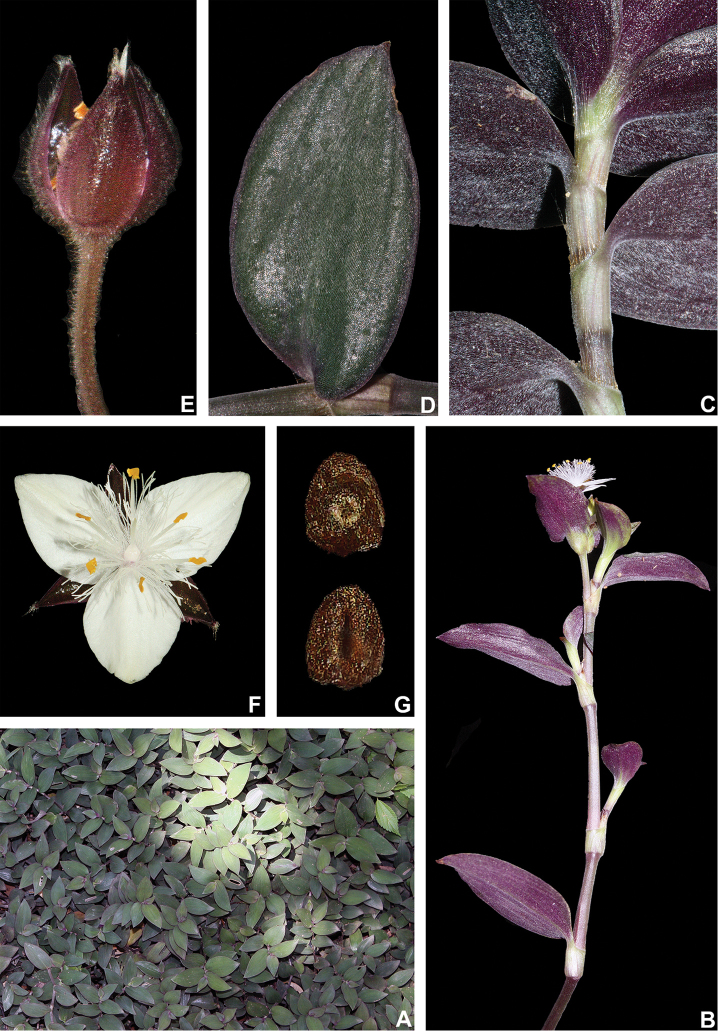
*Tradescantia cymbispatha* C.B.Clarke. **A** habit, showing the velvety aspect of the leaves due to the strigose indumentum **B** detail of the stem, showing the predominant vinaceous colour of the stem and leaves **C** detail of the stems, leaf-sheaths and abaxial side of the blades, showing the strigose and hyaline indumentum **D** adaxial side of the leaf-blade, showing the inconspicuous secondary veins and the strigose and hyaline indumentum **E** detail of flower at post-anthesis, showing the pedicel’s and sepals’ velutine indumentum **F** front view of a flower **G** dorsal and ventral views of the seed, showing the costate testa not cleft towards the embryotega and the hilum ca. ½ the length of the seed. Photos by M.O.O. Pellegrini.

*Tradescantia cymbispatha* is morphologically similar to *T. chrysophylla*, *T. fluminensis* and *T. mundula* due to their indefinite base, prostrate stems with ascending apex (Fig. 16A, B), involute ptyxis, saccate cincinni bracts (Fig. 16B), white petals (Fig. 16F), pistil as long as the stamens (Fig. 16B, E, F), seeds with uncleft testa towards the embryotega and hilum ½ the length of the seed (Fig. 16G). It can be easily differentiated from *T. fluminensis* and *T. mundula* by its sessile leaves with inconspicuous secondary veins (*vs.* subpetiolate with impressed secondary veins, in *T. fluminensis* and *T. mundula*), blades evenly covered by indumentum (*vs.* glabrous or unevenly covered by indumentum) and sepals without dorsal keels (*vs.* sepals dorsally keeled). *Tradescantia cymbispatha* is morphologically more closely related to *T. chrysophylla* due to their sessile and succulent leaves with inconspicuous secondary veins, blades abaxially vinaceous and sepals without dorsal keels. Both species can be differentiated based on pubescence of the vegetative and reproductive organs (leaves strigose, pedicels and sepals velutine, hairs hyaline in *T. cymbispatha vs.* leaves velutine to hispid, pedicels and sepals glandular-pubescent or with a mixture of glandular and eglandular hairs, hairs light brown to golden), symmetry of the cincinni bracts (equal in *T. cymbispatha vs.* unequal in *T. chrysophylla*) and petal colour (white but sometimes pink or lilac in *T. cymbispatha vs.* always white in *T. chrysophylla*). In the field, *T. cymbispatha* is a very distinctive species, being easily differentiated from the remaining species of the *T. fluminensis* group by its adaxially dark to medium bluish-green and abaxially vinaceous leaves, which possess a peculiar velvety glow due to its dense strigose hyaline hairs. The stems are prostrate, forming dense mats that may cover large areas and producing a bluish herbaceous understorey formation. Furthermore, *T. cymbispatha* possesses some very interesting characters that become evident when specimens are dried. Firstly, the intense bluish-green pigmentation of the adaxial side becomes olive-green to medium brown, while the vinaceous pigmentation (i.e. anthocyanin) evaporates, leaving the abaxial side tan to light brown. Also, despite possessing inconspicuous secondary veins due to its succulent leaves, dried leaves of *T. cymbispatha* acquire a striate aspect, due to the large and linearly arranged epidermal domes possessed by this species.

**Figure 17.**
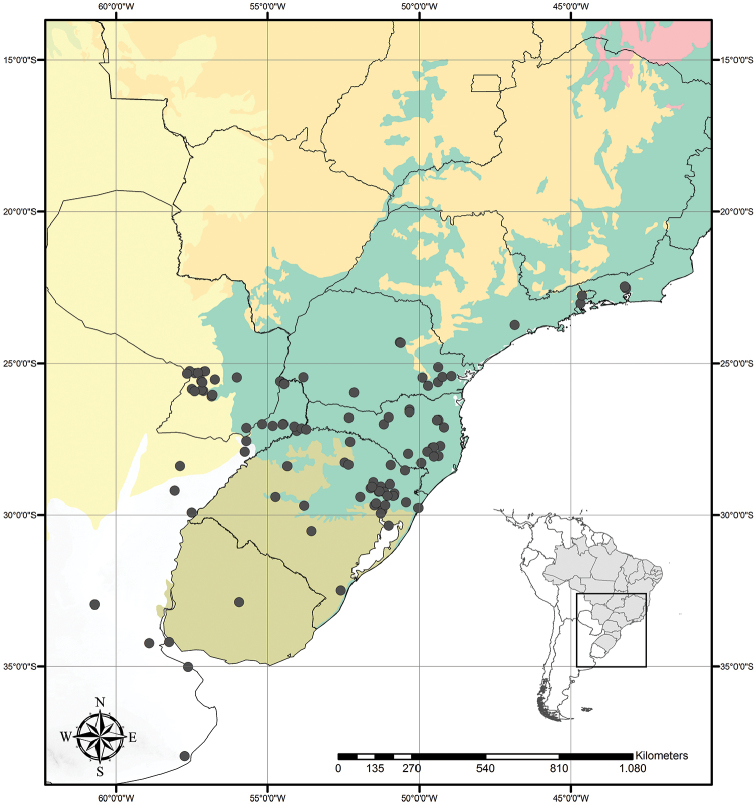
Distribution of *Tradescantia cymbispatha* C.B.Clarke. Orange– Cerrado; Red– Caatinga; Yellow– Chaco and Pantanal; Olive-green– Pampa; Dark green– Atlantic Forest.

#### 6. Tradescantia decora

Taxon classificationPlantaeORDOFAMILIA

W.Bull, Cat. 1892: 3. 1892.

Figs 18 , 19

Tradescantia multibracteata M.Ferrarese et al. in Büneker et al. Phytotaxa 308 (1): 90. 2017. Holotype. BRAZIL. Rio Grande do Sul: Júlio de Castilhos, às margens do rio Toropi, próximo à antiga Usina de Quebra Dentes, fl., 26 Sep 2014, H.M. Büneker et al. 355, (HDCF!; isotype: SMDB n.v.). **Syn. nov.**
Tradescantia valida G.Brückn., Notizbl. Bot. Gart. Berlin–Dahlem 11: 510. 1932. Holotype. BRAZIL. Without province: s.loc., cult. in Hort. Bot. Münster/W., fl., fr., 28 Apr 1932, s.leg. s.n. (B barcode B100296487!). **Syn. nov.**

##### Type material.

**Neotype (designated here).** BRAZIL. Rio Grande do Sul: Jaguari, ca. 12.5 km ao norte de Jaguari na BR-287 em direção a Santiago, fl., fr., Dec 2005, L.Y.S. Aona & M.C. Machado 958 (UEC 2ex barcode UEC057324!; isoneotype to be distributed to: RB!).

##### Description.

*Herbs* ca. 30–70 cm tall, with indefinite base, rupicolous, rarely terrestrial. *Stems* erect, succulent, little branched only at the base; internodes 1.8–7 cm long at base, distally shorter, green, sometimes with vertical reddish-purple striations, glabrous. *Leaves* spirally-alternate, sessile; ptyxis convolute; sheaths 0.4–3.8 cm long, light green, sometimes with vertical green or reddish-purple striations, glabrous, margin setose, with long hyaline hairs; blades 2.7–37.5 × 1.1–2.5 cm, linear elliptic to linear lanceolate to lanceolate, rarely ovate, falcate to complicate, succulent, glabrous, adaxially light to medium green, abaxially light green, rarely tinted vinaceous to completely vinaceous, turning olive-green to light brown when dry, base truncate to obtuse, margin green to vinaceous, setose at base or until the middle with long hyaline hairs, slightly revolute, apex acute to acuminate; midvein conspicuous to inconspicuous, secondary veins inconspicuous, becoming more evident on both sides when dry. *Synflorescences* terminal or axillar in the distal portion of the stems, composed of a solitary main florescence, 1 per leaf axis. *Inflorescences (main florescences)* consisting of a pedunculate 2–3(–5) cincinni fused back to back; peduncles 3.5–6.2 cm long, green, glabrous; basal bract inconspicuous, tubular, hyaline, glabrous; peduncle bracts absent; supernumerary bracts present, 1–3 per inflorescence, similar in shape and size to the cincinni bracts; cincinni bracts 1–3.5 × 0.3–1.2 cm, unequal to each other, lanceolate to ovate, rarely broadly ovate, spathaceous, glabrous, light green, abaxially slightly lighter, base truncate to obtuse, not saccate, margin green, setose at base or until the middle with long hyaline hairs, flat, apex acute; main florescence (4–)6–30-flowered; *Flowers* 1–1.5 cm diam.; floral buds broadly ellipsoid, apex acuminate; pedicels 0.7–2 cm long, green, glabrous, rarely sparsely glandular-pubescent, if present hairs hyaline; sepals 4.8–7.3 × 1.5–3 mm, green, without dorsal keels, glabrous, rarely sparsely pilose at the apex, when present hairs eglandular, hyaline; petals 4–8.6 × 2.7–5.4 mm, flat, white to white with pink apex to light pink; stamens with filaments 2.8–5 mm long, anthers 0.8–1 × 1–1.2 mm; ovary 1–1.7 × 1–1.3 cm, style 4–5.8 cm long; pistil longer than length the stamens. *Capsules* 2.8–4.2 × 1.8–3 mm. *Seeds* 1.1–3 × 1–1.8 mm, cleft towards the embryotega, testa grey to greyish-brown, cleft towards the embryotega, costate; hilum longer than ½ the length of the seed.

##### Specimens seen.

**BRAZIL. Rio Grande do Sul**: Jaguari, gruta linha 1, fr., 27 Dec 1985, J.N.C. Marchiori 149 (HDCF); São Martinho da Serra, Salto do Guassupi, fl., 4 Oct 2015, H.M. Büneker et al. 492 (HDCF, SMDB).

##### Distribution and habitat.

*Tradescantia decora* is endemic to the central region of Rio Grande do Sul, Brazil; in the Atlantic Forest and Pampa domains (Fig. 19). It can be found growing as rupicolous, rarely as a terrestrial, in rocky walls.

##### Phenology.

It was found in bloom and fruit in December and April.

##### Etymology.

The epithet “*decora*” means decorated, ornamented, making reference to this species beautiful appearance, decorated by its lush foliage.

##### Conservation status.

*Tradescantia decora* is only known from five collections restricted to the state of Rio Grande do Sul, one of them cultivated in Berlin and without precise locality. Furthermore, its EOO and AOO are considerably narrow (ca. 672.001 km^2^ and ca. 16.000 km^2^, respectively). According to Bünecker et al. (2017), this species is known to present small subpopulations, with its distribution range being extremely threatened by the construction of small hydroelectric powerplants in the next few years (Marchiori et al. 2014). This might cause most or all known subpopulations to become extinct in the near future, since this species grows in rock walls near water bodies. Thus, in accordance with the IUCN recommendations (IUCN 2001), *T. decora* should be considered as Critically Endangered [CR, A2ac+B2ab(iii, iv, v)+D1+E].

##### Nomenclatural notes.

After analysing the original publication (Bull 1892), I have concluded that, like most species described in Seed and Nursery Catalogues, *T. decora* was probably described based on living and cultivated material. Thus, no voucher was ever made for this name. Unfortunately, Bull (1892) does not present any kind of illustration that might be selected as the lectotype for his name. Thus, according to The Code (McNeill et al. 2012, Art. 9.7), I designate the specimen *Aona & Machado 958* (UEC barcode UEC057324) as the neotype of *T. decora* since it is in complete accordance with the protologue.

##### Comments.

**Figure 18.**
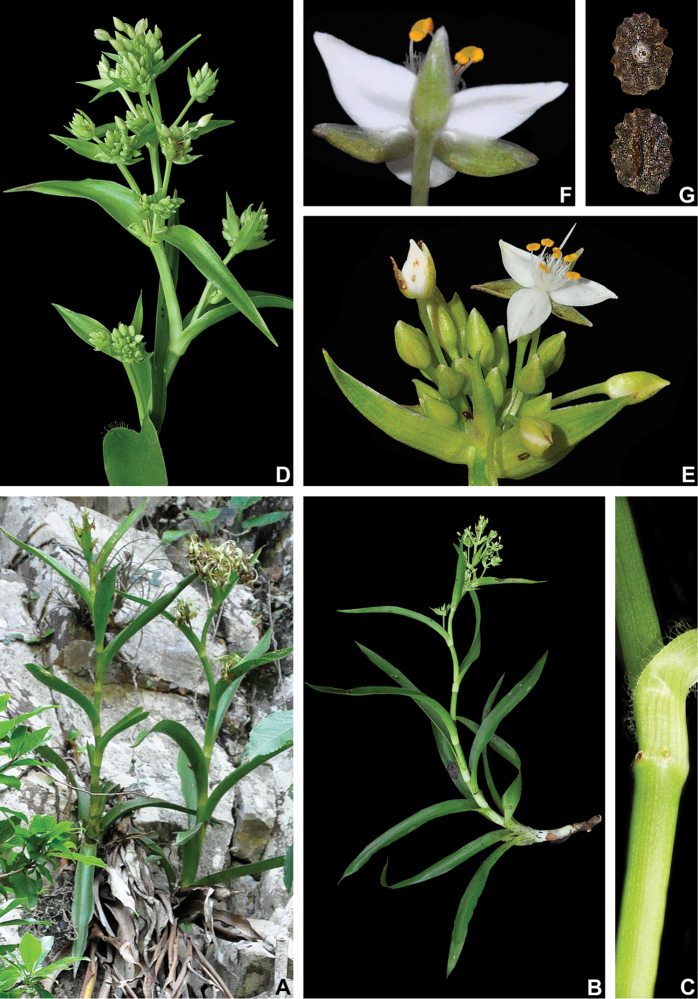
*Tradescantia decora* W.Bull. **A** detail of a rupicolous subpopulation **B** habit, showing the erect stem and spirally-alternate leaves with linear to linear lanceolate and canaliculate blades **C** detail of the stem and leaf-sheath, showing the glabrous internodes and the long-setose margin of the leaf-sheath and base of the blade **D** synflorescence, showing florescences ranging from double-cincinni to 3–5-cincinni and the leaf-blades with basally setose margins **E** inflorescence composed of 4-cincinni, showing the side view of a flower and its pistil longer than the stamens **F** lower view of a flower, showing the glabrescent sepals **G** dorsal and ventral views of the seed, showing the costate testa cleft towards the embryotega and the hilum longer than ½ the length of the seed. **C** & **G** by M.O.O. Pellegrini, remaining photos by H.M. Büneker.

**Figure 19.**
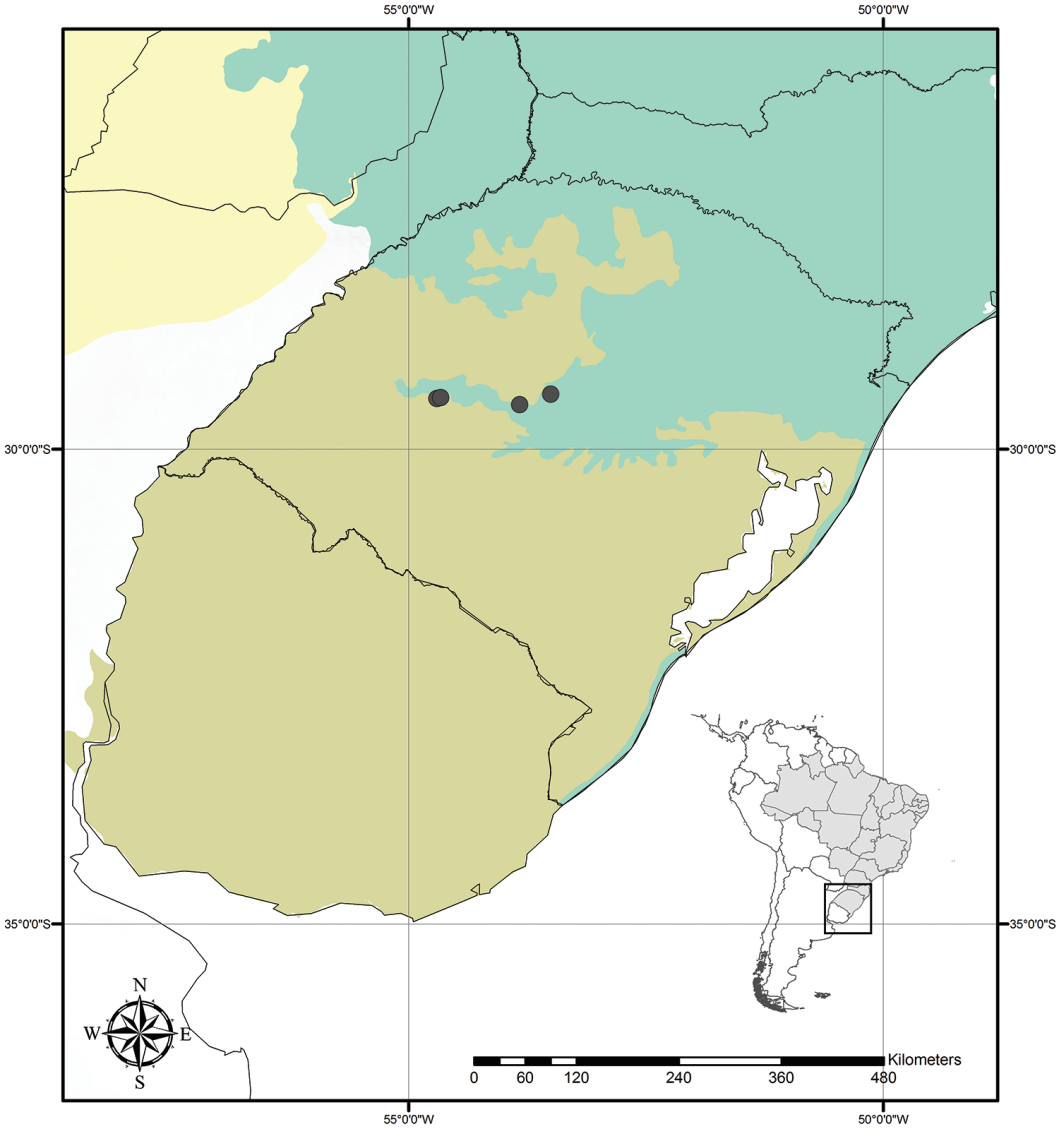
Distribution of *Tradescantia decora* W.Bull. Yellow– Chaco and Pantanal; Olive-green– Pampa; Dark green– Atlantic Forest.

After analysing the protologue of *T. decora* (Bull 1892), it became clear that that this name was conspecific to *T. valida* and *T. multibracteata*, due to its erect stems (Fig. 18A, B), spirally-alternate leaves (Fig. 18A, B), linear lanceolate to lanceolate leaf-blades with truncate base (Fig. 18B)) and, most importantly, all being restricted to the state of Rio Grande do Sul, Brazil (Fig. 19). Since *T. decora* has priority over *T. valida* and *T. multibracteata*, it should be treated as the accepted name for this species. *Tradescantia decora* can be easily differentiated from all remaining species of the *T. crassula* group by the presence of supernumerary bracts, its spathaceous and unequal cincinni bracts, its main florescence being generally composed by 2–3(–5) cincinni (Fig. 18D, E) and sepals not keeled with caducous hairs at the apex (Fig. 18E, F). It is similar to *T. cerinthoides* due to its sepals without dorsal keels (Fig. 18E, F). Nonetheless, they can be easily differentiated due to its generally linear elliptic to linear lanceolate to lanceolate leaf-blades (*vs.* elliptic to broadly elliptic or ovate to broadly ovate or obovate to broadly obovate, in *T. cerinthoides*), glabrous with margins setose at the base or until the middle (*vs.* pubescent on both sides or only abaxially, rarely glabrous on both sides and ciliate margins) and pedicels and sepals glabrous or only sparsely pubescent at apex with eglandular hairs (*vs.* evenly densely velutine to hispid, sometimes with a mixture of glandular and eglandular hairs). *Tradescantia decora* is much more similar to *T. crassula* and *T. seubertiana*, due to their leaf-blades and sepal pubescence. These species can be easily differentiated by the pubescence of the margin of their leaf-sheaths (ciliate to shortly-setose in *T. crassula*; glabrous in *T. seubertiana*; and long-setose in *T. decora*), the pubescence of their sepals (long-setose along the keels in *T. crassula*; glabrous in *T. seubertiana*; and glabrous or with few hairs at the apex in *T. decora*) and by the shape of their floral buds (broadly ovoid *T. crassula*; ellipsoid in *T. seubertiana*; and ellipsoid in *T. decora*).

#### 7. Tradescantia fluminensis

Taxon classificationPlantaeORDOFAMILIA

Vell., Fl. Flumin.: 140. 1829.

Figs 20 , 21

Tradescantia albiflora Kunth, Enum. Pl. 4: 84. 1843. Lectotype (designated here). s.loc., cultivated in Germany, Horto Berolinense, fl., s.dat., C.S. Kunth 165 (B barcode B100264915!; isolectotype: B barcode B100264916!).
Tradescantia fluminensis f. albovittata (Pynaert) Voss in Siebert & Voss, Vilm. Blumengärtn. ed. 3, 1: 1144. 1895. **Syn. nov.**
Tradescantia albovittata Pynaert in Burvenich et al., Rev. Hort. Belge Étrangère (Eds Burvenich et al.) 11: 205. 1885. Lectotype (designated here). [illustration] Original chromolithograph at the Bureau de la Revue, Ghent, and later published in Burvenich et al., Rev. Hort. Belge Étrangère 11: t. 16. 1885. **Syn. nov.**
Tradescantia fluminensis f. aureovittata Voss in Siebert & Voss, Vilm. Blumengärtn. ed. 3, 1: 1143. 1895. Type. not found. **Syn. nov.**
Tradescantia laekenensis L.H.Bailey & E.Z.Bailey, Hortus: 616. 1930, nom. nud.
Tradescantia striata L.H.Bailey & E.Z.Bailey, Hortus: 616. 1930, nom. nud.

##### Type material.

**Lectotype (designated by Pellegrini et al. 2015).** [illustration] Original parchment plate of “*Flora fluminensis*” in the Manuscript Section of the Biblioteca Nacional of Rio de Janeiro [cat. no.: mss1198652_156] and later published in Vellozo, Fl. Flumin. Icones 3: t. 152. 1831.

##### Description.

*Herbs* ca. 15–50 cm tall, with an indefinite base, terrestrial, rupicolous or epiphytes. *Stems* prostrate with ascending apex, delicate to slightly succulent, little to densely branched; internodes 1.6–9 cm long at base, distally shorter, medium to dark green, glabrous, with a leaf-opposed longitudinal line of short, uniseriate, hyaline hairs in the terminal portion of the stems. *Leaves* distichously-alternate, subpetiolate; ptyxis involute; sheaths 0.2–1.3 cm long, medium green, glabrous, margin densely setose, hairs hyaline to light brown; petiole 0.2–1.5 cm long to indistinct; blades (1.3–1.6–)2.2–11.8 × (0.6–0.8–)1.1–4.8 cm, elliptic to broadly elliptic or ovate to broadly ovate, flat, membranous to slightly succulent, glabrous on both sides, adaxially dark to medium green, abaxially light to medium green, turning olive-green or medium to dark brown when dry, base cordate to obtuse, rarely cuneate, margin green, ciliolate, flat, apex acute; midvein conspicuous, adaxially impressed, secondary veins conspicuous, adaxially impressed, abaxially conspicuous, becoming more evident on both sides when dry. *Synflorescences* terminal or axillar in the distal portion of the stems, composed of a solitary main florescence, 1 per leaf axis. *Inflorescences (main florescences)* consisting of a pedunculate double-cincinni fused back to back; peduncles 0.6–4.7 cm long, dark to medium green, glabrous to sparsely pilose near the cincinni bracts, with a longitudinal line of short, uniseriate, hyaline hairs, opposed to the first cincinni bract; cincinni bracts 1.1–5.7 × 0.5–2.6 cm, similar to each other, ovate to broadly ovate, leaf-like, glabrous, medium to dark green, abaxially light to medium green, base cordate to obtuse, saccate, margin ciliolate, sometimes sparsely setose at base, flat, apex acute; double cincinni (4–)6–12-flowered. *Flowers* 1.5–2 cm diam., pedicels 0.6–1.5 cm long, medium green, glabrous to distally sparsely glandular-pubescent, hairs hyaline; floral buds ovoid; sepals 4.6–6.7 × 2.6–4.4 mm, dorsally keeled, medium green, pilose along the keel; petals 0.7–1 × 0.4–0.6 mm, plicate, white; filaments 5–5.2 mm long, anthers 0.8–1 × 0.9–1 mm; ovary 1–2.1 × 0.9–1.3 mm, style 3.5–4.4 mm long; pistil the same length as the stamens. *Capsules* 3.3–4.1 × 2.7–2.9 cm. *Seeds* 1.4–1.6 × 1.1–1.2 mm, testa brown to greyish-brown, not cleft towards the embryotega, costate; hilum equal ½ the length of the seed.

##### Examined material.

**ARGENTINA. Buenos Aires**: Berazategui, Conchitas, fl., Dec 1919, A. Castellanos 794 (BA); La Plata, fl., 18 Oct 1928, A.L. Cabrera 441 (LP); fl., 15 Oct 1941, A.L. Cabrera 9796 (LP). **Chaco**: 1° de Mayo, Colonia Benítez, fl., 9 Oct 1970, A.G. Schulz 17677 (CORD, CTES, SI). **Córdoba**: Córdoba, fl., 24 Oct 1902, T.J.V. Stuckert 11937 (CORD); fl., 25 Nov 1902, T.J.V. Stuckert 12151 (CORD); Punilla, Capilla del Monte, en las proximidades del Cerro El Zapato, fl., 30 May 1955, M.M. Job s.n. (BA no. 29081). **Corrientes**: Mercedes, Macrosistema Ibera, Estancia Rincon del Diablo, fl., 28 Aug 1998, M.M. Arbo et al. 8010 (CORD, CTES); Santo Tomé, Garruchos, destacamento de Prefectura a orillas del Río Uruguay, fl., 6 Sep 1993, M.M. Arbo et al. 5826 (CTES). **Misiones**: Cainguás, Predio UNLP Reserva Privada Valle del Arroyo Cuña Pirú, márgenes del arroyo Cuña Pirú, cerca del balneário, fl., 14 Mar 2000, F. Biganzoli et al. 800 (CORD); Candelaria, Bonpland, fl., Oct 1906, H. Van de Venne 67 (BA); Guaraní, Ruta Nacional 14, Km 315, camino de San Vicente a San Pedro, desvío hacia el obraje de M. Carré, 13 km de R-14, fl., 13 Feb 1996, O. Morrone et al. 747 (CORD); San Ignacio, Teyucuaré, Peñón Reina Victoria, fl., 25 Sep 1972, A. Schinini 5603 (CORD); San Pedro, Parque Provincial Cruce Caballero, picada al NW, fl., 23 Sep 1997, F.O. Zuloaga & O. Morrone 6478 (CORD). **Santa Fe**: Santa Fe, fl., 1 Sep 1984, A. Gagneten 25 (CORD). **BRAZIL. Espírito Santo**: Aracruz, Guaraná, près Ribeirão do Meio, fl., 30 Jul 1993, M. Pignal H310 (BAH, P); Castelo, Parque Estadual do Forno Grande, fl., 1 Nov 2004, L. Kollmann 7221 (MBML); trilha para o Forninho, fl., 15 Oct 2008, R.C. Forzza et al. 5334 (CEPEC, MBML, RB, UPCB); Vargem Alta, fl., 2 Sep 1946, A.C. Brade 19416 (RB); Domingos Martins, between São Pedro de Urânia and Pedra Azul, fl., 31 Jul 1986, T.B. Croat 61837 (MO, R); Santa Maria de Jequitibá, Pedra do Garrafão, fl., 23 Aug 2015, M.C. Almeida 121 (R). **Minas Gerais**: Caeté, alto da Serra da Piedade, fl., 9 Nov 2002, J.A. Lombardi 4933 (HRCB, US); Caldas, Serra de São Domingos, fl., 28 Feb 2013, M.O.O. Pellegrini & J.F. Barbosa 321 (RB); Tiradentes, Serra de São José, fl., fr., 3 Oct 1987, M. Peron 358 (RB). **Paraná**: Carambeí, Catanduva de Fora, fl., 25 Aug 2013, M.E. Engels 1469 (ALCB, FURB, HCF, JOI, MBM, RB, UNOP, UPCB); Catanduvas, Barra do Guarani, fl., 10 Oct 1974, G. Hatschbach & P. Pelanda 35124 (K, MBM); Curitiba, Parque Barigui, fl., fr., 10 Nov 1970, L.T. Drombowski & Y.S. Kuniyoshi 3069 (MBM, US); fl., 18 Nov 197, R. Kummrow 107 (K, MBM); Campina do Siqueira, fl., 20 Nov 1966, C. Stellfeld 1643 (UPCB, US); Jardim Botânico de Curitiba, fl., 26 Aug 1993, J. Cordeiro & J.M. Silva 1092 (FLOR, MBM, UEC, US); fl., 24 Sep 2014, M.O.O. Pellegrini et al. 407 (US); Parque Municipal Capão da Embuia, fl., 25 Sep 2014, M.O.O. Pellegrini et al. 410 (RB); Itapecerica da Serra, Aldeinha, BR-116, km 299, fl., fr., 5 Oct 1998, L.N. Hara s.n. (FUEL no. 29803); Mariópolis, rodovia Pato Branco-Palmas, km 205, fl., 21 Nov 1999, D.C. Rocha s.n. (BOTU no. 23008); Palmeira, fl., s.dat., T.A. Preston s.n. (K barcode K001248126); São José dos Pinhais, Caminho do Vinho, área de plantação de agrião, fl., fr., 25 Sep 2014, M.O.O. Pellegrini et al. 413 (RB). **Rio de Janeiro**: s.loc., fl., fr., s.dat., G. Gardner s.n. (K barcode K001248117); fl., s.dat., C. Gaudichaud 340 (P); fl., s.dat., J. Miers 3031 (K); fl., fr., s.dat., F. Sellow 186 (K); environs de Rio de Janeiro, fl., 1843, M. Weddel 180 (P); fl., fr., 1843, M. Weddel 595 (P); fl., 1843, M. Weddel 598 (P); Guapimirim, Granja Monte Olivete, margem do Rio Bananal, fl., 17 Nov 1993, J.M.A. Braga et al. 844 (RB); Rio Bananal, fl., fr., 16 Aug 1995, J.A. Lira Neto et al. 94 (RB); Mangaratiba, Reserva Ecológica do Rio das Pedras, próximo ao poço do Rio Grande, fl., fr., 31 Nov 1996, J.A. Lira Neto et al. 472 (RB); Nova Friburgo, Morro da Caledônia, fl., fr., 8 Jun 1977, G. Martinelli et al. 2465 (RB); Macaé de Cima, Fazenda Ouro Verde, fl., 30 Jul 1994, C.M. Vieira et al. 610 (RB); fl., 26 Jan 2012, M.O.O. Pellegrini et al. 208 (RB); Mury, Cachoeira do Rio das Flores, fl., fr., 24 Jul 1986, M. Leitman 135 (RB); fl., 10 Oct 2004, A.F.P. Machado 363 (RB, SPF); Petrópolis, fl., s.dat., T.A. Preston s.n. (K barcode K001248127); prope Petrópolis, fl., fr., 10–16 Jul 1882, J. Bal s.n. (K barcode K001248130); Cascatinha, fl., fr., 18 Jul 1943, O.C. Goés & D. Constantino 348 (RB); fl., fr., Jul 1944, O.C. Goés & E. Dionisio 797 (RB); Estrada da Saudade, Morro Seco, fl., fr., Dec 1943, O.C. Goés & E. Constantino 920 (RB); fl., Castelânea, 8 Sep 2010, M.O.O. Pellegrini 48 (RB, RFA); Resende, Parque Nacional do Itatiaia, margem do Rio Campo Belo, perto do lote 17, fl., 17 Oct 1977, V.F. Ferreira & Briolanjo 136 (RB); Rio Claro, Lídice, Parque Estadual Cunhambebe, caminho para o Alto da Serra, 7 Aug 2013, fl., fr., B.C. Bandeira et al. 256 (RB); Rio de Janeiro, Serra do Andaraí, fl., 19 Dec 1946, L.E. Mello-Filho 529 (R); Tijuca, caminho do Morro do Archer, fl., fr., 10 Nov 1948, A.C. Brade et al. s.n. (RB no. 65375); Parque Nacional da Tijuca, fl., fr., 27 Oct 1995, M.G. Bovini et al. 899 (RB); Corcovado, fl., fr., 1837, G. Gardner 135 (K, P); Paineiras, fl., fr., 5 Oct 1879, R. Galvão 143 (P); trilha para o Morro da Cocanha, Alto da Boa Vista, fl., fr., 16 Jan 1994, J.M.A. Braga & R. Neves 943 (RB); fl., 1 Nov 1996, J.M.A. Braga 3647 (RB); Vargem Grande, Morro do Manga Larga, fl., fr., 2 Dec 1995, C.M. Vieira et al. 758 (RB); Santa Maria Madalena, fl., fr., s.dat., A. Lisbôa s.n. (RB no. 2653); Fazenda da Boa Fé, fl., 11 Sep 1942, L.E. Mello-Filho 88 (R); Mata do Garrafão, fl., May 1917, A. Sampaio 2655 (R); Parque Nacional da Serra dos Órgãos, Trilha Suspensa, fl., 23 Sep 2011, C.P. Bruniera et al. 385 (RB, SPFR); fl., 22 Oct 2012, D.O. Dinato et al. 11 (HRCB, UPCB); Praça da Barragem, fl., 12 Sep 2014, L.S.B. Calazans et al. 448 (RB); Venda Nova, fl., 22 Aug 2004, C.H.R. Paula 650 (RB). **Rio Grande do Sul**: Arroio do Ouro, Vale Real, fl., 31 Aug 1998, S. Diesel 1521 (US); Cachoeira do Sul, Fazenda São Carlos, fl., Sep 1987, N. Silveira & J. Mattos 6681 (HAS); Canela, fl., 10 Nov 2006, M.L. Guedes 12706 (ALCB); Erehim, campus da Uri, fl., 21 Oct 1993, A. Butzke 10824 (US); Esteio, prope Porte Alegre, fl., fr., 24 Nov 1948, B. Rambo 38326 (HBR); Centro dos Trabalhadores, fl., Nov 1974, M.L. Porto & L. Aguiar s.n. (HAS no. 1533); Farroupilha, fl., 26 Oct 1956, O.R. Camargo 885 (PACA); Montenegro, Pareci, fl., 10 Oct 1945, E. Henz s.n. (PACA no. 32711); fl., 17 Aug 1949, B. Rambo 43004 (K, LIL, PACA); Porto Alegre, fl., s.dat., O.R. Camargo 2454 (PACA); Morro do Sabiá, fl., 5 Oct 1949, B. Rambo 43764 (K, LIL, PACA); Vila Manresa, fl., 27 Nov 1945, B. Rambo 30667 (K, PACA); Reserva Biológica do Lami, fl., Sep 2003, A.S. Mello & D. Fuhro s.n. (HAS no. 45477); São Francisco de Paula, Cambara, fl., fr., Feb 1948, R. Rambo 36590 (PACA); São Leopoldo, fl., 1907, F. Theissen s.n. (PACA no. 25293); Silveira Martins, fl., 6 Oct 2011, G.A. Dettke & J. Durigon 959 (ICN); Taquarí, Estação Experimental de Pomicultura, fl., 8 Nov 1958, O.R. Camargo 3319 (PACA); Triúnfo, Bom Jardim, CMN 7, fl., Aug 1977, I. Ungaretti 532 (HAS); Viamão, bairro Tarumã, região do entorno do Lago Tarumã, fl., s.dat., P.J.S. Silva Filho 1597 (ICN). **Santa Catarina**: Bocaina do Sul, Pessegueiros, fl., 8 Nov 2010, G.A. Dettke 476 (ICN); Bom Jardim da Serra, Serra do Rio do Rastro, fl., s.dat., M. Sobral et al. 9019 (ICN); Ibirama, fl., 20 Oct 1953, R.M. Klein 614 (HBR); Itapiranga, proximidades do Rio Uruguai, fl., 6 Feb 1951, B. Rambo 49832 (PACA); Rodeio, fl., 19 Oct 2015, L.A. Funez 4550 (FURB); São Bento do Sul, Trilha do Parque Florestal do SAMAE, pequeno remanescente de mata próximo a estrada, fl., 28 Sep 2014, M.O.O. Pellegrini & P. Schwirkowski 420 (RB); São Francisco do Sul, fl., 13 Oct 2008, T.G. Fendrich 7136 (HUFSJ, JOI); Timbó, Centro, margem do Rio dos Cedros, fl., 11 Oct 2014, L.A. Funez 3558 (FURB); Turvo, Arar, 20.x.1943, fl., R. Reitz 73 (HBR, RB). **São Paulo**: s.loc., fl., Aug 1942, s.leg. s.n. (CESJ no. 812, HAS no. 67752); Biritiba Mirim, Estação Biológica de Boracéia, fl., 29 Sep 1983, A. Custódio Filho 1568 (RB, SP); Campinas, Fazenda Santa Eliza, Monjolinho, fl., 9 Sep 2004, J.L.M. Aranha Filho et al. 26 (UEC); Cunha, fl., 7 Sep 2015, A. Maruyama & L. Cicco 40 (SPSF); fl., 12 Oct 2015, A. Maruyama & L. Cicco 134 (SPSF); fl., 12 Oct 2015, A. Maruyama & L. Cicco 151 (SPSF); Reserva Estadual de Cunha, Instituto Florestal, Secretaria de Agricultura, fl., fr., 20 Nov 1979, A. Fonseca Vaz 294 (RADAM, RB); Itapeva, rodovia Francisco Alves Negrão, SP-258, entre Itapeva-Itararé, km 289, fl., 23 Oct 2007, R.C. Forzza et al. 4765 (CEPEC, RB, SPF); Santo André, Reserva Biológica do Alto da Serra de Paranapiacaba, fl., 11 Oct 2006, M. Kirizawa 3550 (RB, SP); São Paulo, Butantã, fl., 16 Sep 1919, F.C. Hoehne s.n. (SP no. 3414); Mandaqui, fl., May 1913, Toledo 637 (RB); Jardim Botânico e Parque do estado, fl., 12 Aug 1968, T. Sendulksy 981 (RB, SP); mata do Instituto de Botânica de São Paulo, fl., 22 Nov 1976, F.S. Cavalcante & I.T. Menezes 5 (RB, SP); arredores do Castelinho do Instituto de Botânica de São Paulo, fl., 21 Nov 1980, N.A. Rosa & J.M. Pires 3824 (INPA, K); fl., 14 Oct 1982, M.C.B. Attié et al. 52 (RB, SP); Reserva da Cidade Universitária Armando de Salles Oliveira, fl., 18 Aug 1998, M. Groppo Jr. 345 (SP, SPF); Reserva Biológica do Parque Estadual das Fontes do Ipiranga, fl., 20 Apr 1979, M. Kirizawa 418 (RB, SP); fl., 20 Jun 1979, M. Kirizawa 419 (RB, SP); fl., 25 Jun 1979, J.A. Correa 94 (RB, SP); fl., 9 Sep 1980, M.G.L. Wanderley 138 (RB, SP); fl., 1 Sep 1982, R.D. Marassi 5 (K, RB, SP). **PARAGUAY. Itapúa**: Isla Yacyretá, Zona de transición, extremo de dunas, fl., 14 Aug 1992, A.B. Pin 207 (CORD, CTES). **URUGUAY. Without province**: s.loc., fl., 23 Feb 1912, P. Serre s.n. (P barcode P01639779). **Canelones**: Santa Lucia, fl., 14 Nov 1922, H.M. Smith 63 (US). **Montevideo**: s.loc., fl., s.dat., J. Tweedie s.n. (K); Malvin, fl., s.dat., F. Felippone 4565 (CORD); Nico Perez, fl., 25 Nov 1947, W.G.F. Herter 436d (SI, US); Peniarolli, fl., Oct 1926, W.G.F. Herter 436a (P).

##### Distribution and habitat.

Argentina and Brazil (states of Espírito Santo, Rio de Janeiro, Minas Gerais, São Paulo, Paraná, Santa Catarina and Rio Grande do Sul), Paraguay and Uruguay; in the Atlantic Forest, Cerrado, Chaco and Pampa domains (Fig. 21). It can be found growing as terrestrial or epiphyte understorey in shaded moist forests, as a weed and at roadsides.

##### Phenology.

It was found in bloom throughout the year but peaking during the rainy season and being less commonly in bloom during the dry season. Fruiting seems to be uncommon, with most fruiting specimens recorded in the state of Rio de Janeiro, Brazil.

##### Etymology.

The epithet “*fluminensis*” makes reference to the region the species was originally described, the state of Rio de Janeiro.

##### Conservation status.

*Tradescantia fluminensis* possesses a wide EOO (ca. 1,440,097.589 km^2^), being also an aggressive weed worldwide known to occur in Australia, Bermuda, Italy, Japan, Kenya, New Zealand, Portugal, Puerto Rico, Russia, Saint Lucia, South Africa, Swaziland and USA. (Hunt 2001; Burns 2008; Fowler et al. 2013; GSID 2015; pers. observ.). Thus, following the IUCN recommendations (IUCN 2001), it should be considered Least Concern (LC).

##### Nomenclatural notes.

*Tradescantia fluminensis* was, together with *T. geniculata* Vell. (≡ *T. cymbispatha*), the first two species currently placed in T. subg. Austrotradescantia to be described (Vellozo 1829, 1831). They were both described in the infamous *Florae fluminensis* by Friar José Mariano da Conceição Vellozo, based on specimens collected by him in the state of Rio de Janeiro (Pellegrini et al. 2015).

When describing *T. albovittata*, Pynaert (in Burvenich et al. 1885) makes no reference to any herbarium specimen. Nonetheless, the author presents a gorgeous chromolithograph that shows the habit of the plant and its characteristic white-striped leaves. Thus, I designate the original illustration as the lectotype of *T. albovittata* and reduce it to a synonym of *T. fluminensis*.

According to Stafleu and Cowan (1986), it is unknown where the specimens of any names described by A. Voss are housed. Voss (in Siebert and Voss 1895), makes no reference to any kind of studied specimen and presents no illustration for most of his names, which prevents the designation of lectotypes. Since no specimens matching the diagnosis of T. fluminensis f. aureovittata were located, I was unable to designate neotypes for it.

##### Comments.

**Figure 20.**
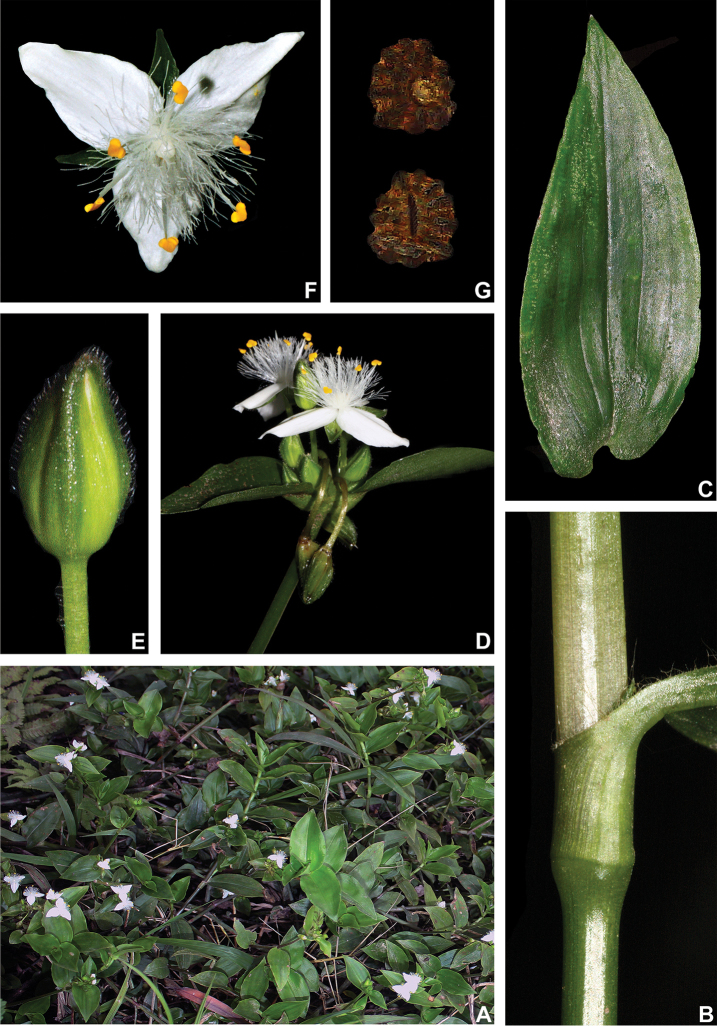
*Tradescantia fluminensis* Vell. **A** habit, showing the mat-forming prostrate stems **B** detail of the stem and leaf-sheath, showing the setose margin of the leaf-sheath and the subpetiole **C** adaxial side of the leaf-blade, showing the impressed secondary veins **D** detail of the inflorescence, showing the saccate cincinni bract base, the plicate petals and the pistil as long as the stamens **E** floral bud, showing the dorsally keeled sepals and the pilose indumentum restricted to the keels **F** front view of a flower, showing the plicate petals **G** dorsal and ventral views of the seed, showing the costate testa not cleft towards the embryotega and the hilum ca. ½ the length of the seed. Photos by M.O.O. Pellegrini.

The name *T. fluminensis* has been misapplied to almost all species of T. subg. Austrotradescantia, even to species from the *T. crassula* group. Here I consider *T. fluminensis* as a much lesser variable entity than accepted by previous authors (e.g. Seubert 1871; Clarke 1881; Hunt 1980). *Tradescantia fluminensis* is morphologically similar to *T. cymbispatha*, *T. chrysophylla*, *T. hertweckii*, *T. mundula* and *T. umbraculifera* due to their indefinite base, prostrate stems with ascending apex (Fig. 20A), involute ptyxis, saccate cincinni bracts (Fig. 20D), white petals (Fig. 20D, F), seeds with uncleft testa towards the embryotega and hilum ½ the length of the seed (Fig. 20G). It can be easily differentiated from *T. cymbispatha* and *T. chrysophylla* by its subpetiolate leaves with impressed secondary veins (*vs.* sessile leaves with inconspicuous secondary veins, in *T. cymbispatha* and *T. chrysophylla*), blades glabrous or unevenly covered by indumentum (*vs.* evenly covered by indumentum) and sepals dorsally keeled (*vs.* sepals without dorsal keels). It is morphologically more closely related to *T. hertweckii*, *T. mundula* and *T. umbraculifera* due to their leaves with impressed secondary veins, dorsally keeled sepals and petals always white. *Tradescantia fluminensis* can be differentiated from *T. mundula* due to its glabrous stems (*vs.* strigose in *T. mundula*), leaves glabrous (*vs.* unevenly to evenly strigose), blades membranous to slightly succulent and abaxially light to medium green (*vs.* chartaceous and abaxially completely to partially vinaceous), sepals pilose with hairs restricted to the keels (*vs.* evenly velutine) and plicate petals (*vs.* flat). *Tradescantia fluminensis* can be differentiated from *T. umbraculifera* due to its subpetiolate leaves (*vs.* sessile in *T. umbraculifera*), leaf-like cincinni bracts (*vs.* spathaceous), pedicels green at anthesis (*vs.* white), petals plicate (*vs.* flat) and pistil as long as the stamens (*vs.* longer than the stamens). *Tradescantia fluminensis* is easily identified in the field, due to its emerald green and glossy leaves, that give healthy plants a characteristic plastic aspect.

As aforementioned, *T. fluminensis* is a popular potted plant, as well as an aggressive weed worldwide. However, many cultivated specimens or weedy populations studied by me actually represent other species from T. subg. Austrotradescantia. In cultivation, many plants are referred to as *T. albiflora* Kunth, which is here kept as a synonym of *T. fluminensis*. Nonetheless, they actually represent specimens of *T. mundula* and, more rarely, specimens of *T. cymbispatha* and *T. crassula*. Alternatively, the only other species of T. subg. Austrotradescantia known to me to have been introduced in cultivation is the pink and lilac flowered forms of *T. cerinthoides*, generally treated by gardeners as *T. blossfeldiana*. In cultivation, *T. fluminensis* rarely set seeds and commonly reproduces itself by stem fragmentation. Seed production seems to be also uncommon throughout most of its native range, being only recurrently observed in the state of Rio de Janeiro, Brazil. The reason for this is unknown, since all observed native populations and all specimens kept in cultivation were consistently seen being visited by several insects. My hypothesis is that *T. fluminensis* actually represents a self-incompatible species and thus some subpopulations are incapable of producing seeds, since they might be exclusively composed of clonal individuals. I believe that, in light of the present taxonomic revision, the reproductive biology of T. subg. Austrotradescantia, especially of the *T. fluminensis* group, should be properly studied.

**Figure 21.**
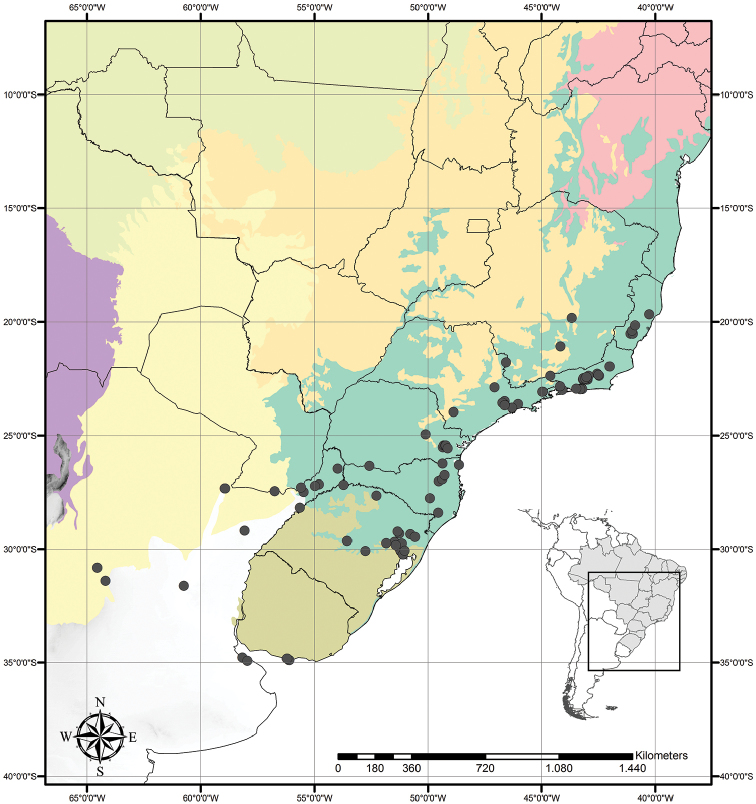
Distribution of *Tradescantia fluminensis* Vell. Light green– Amazon Forest; Orange– Cerrado; Red– Caatinga; Yellow– Chaco and Pantanal; Olive-green– Pampa; Dark green– Atlantic Forest; Purple– Andean Yungas.

#### 8. Tradescantia hertweckii

Taxon classificationPlantaeORDOFAMILIA

M.Pell. sp. nov.

urn:lsid:ipni.org:names:77185916-1

Figs 22 , 23

##### Diagnosis.

Similar to *T. fluminensis* due to its indefinite base, stems prostrate with ascending apex, involute ptyxis, leaf-blades with conspicuous secondary veins, saccate cincinni bracts, ovoid floral buds, keeled sepals, pistil the same length as the stamens and seeds with costate testa. It can be differentiated by its sessile leaves, blades hispid, margins ciliate, but setose at base, sepals setose along the keel, petals flat and hilum longer than ½ the length of the seed.

##### Type.

BRAZIL. Rio de Janeiro: Paraty, estrada para o Pico do Coriscão, próximo a um rio, 222 m.s.m., 23°14'99"S, 44°47'68"W, fl., fr., 18 Dec 2007, M.G. Bovini et al. 2694 (holotype: RB barcode RB00537845!; isotypes to be distributed to: R!, SPF!, US!).

##### Description.

*Herbs* ca. 30–60 cm tall, with an indefinite base, terrestrial. *Stems* prostrate with ascending apex, delicate to slightly succulent, branched to densely branched; internodes 1.8–4.5 cm long at base, distally shorter, medium to dark green, glabrous, with a leaf-opposed dense longitudinal line of short, uniseriate, brown to light brown hairs. *Leaves* distichously-alternate, sessile; ptyxis involute; sheaths 4.1–7.6 mm long, medium green, glabrous, with a dense setose line of uniseriate hairs opposed to the blade, margin densely setose, hairs light to medium brown; blades 6.4–13.6 × 1.9–3.8 cm, lanceolate to elliptic to linear oblong, flat, membranous, adaxially sparsely hispid to hispid, abaxially hispid, adaxially dark to medium green, abaxially light to medium green, turning olive-green or medium brown when dry, base rounded to cordate, margins green, ciliate, setose at base, flat, apex acuminate; midvein conspicuous, adaxially impressed, secondary veins conspicuous, adaxially impressed, abaxially inconspicuous, becoming more evident on both sides when dry. *Synflorescences* terminal or axillar in the distal portion of the stems, composed of a solitary main florescence, 1 per leaf axis. *Inflorescences (main florescences)* consisting of a pedunculate double-cincinni fused back to back; peduncles 1.9–3.8 cm long, medium to dark green, glabrous, with a dense longitudinal line of short, uniseriate, light to medium brown hairs; cincinni bracts 3.8–7.7 × 1–2.6 cm, unequal to strongly unequal to each other, lanceolate to ovate, leaf-like, adaxially sparsely hispid to hispid, abaxially hispid, adaxially dark to medium green, abaxially light to medium green, base cordate to obtuse, saccate, margin ciliate, setose at base, flat, apex acuminate; double-cincinni 6–12-flowered. *Flowers* 1.2–1.5 cm diam., pedicels 0.5–1.3 cm long, medium to dark green, distally sparsely glandular-pubescent, hairs hyaline; floral buds ovoid; sepals 4.6–6.5 × 3–4.8 mm, dorsally keeled, medium green, setose along the keel, hairs hyaline; petals 5.9–7.5 × 3–4.3 mm, flat, white; filaments 4.9–5.8 mm long, anthers 0.4–0.6 × 0.6–0.9 mm; ovary 1.3–1.7 × 1.1–1.3 cm, style 4–5.1 mm long; pistil the same length as the stamens. *Capsules* 4.2–5 × 3.4–4.3 cm. *Seeds* 1.5–1.8 × 1.2–1.4 mm, testa light to medium grey, not cleft towards the embryotega, costate; hilum longer than ½ the length of the seed.

##### Distribution and habitat.

*Tradescantia hertweckii* is endemic to Brazil, more precisely to the state of Rio de Janeiro, municipality of Paraty; in the Atlantic Forest domains (Fig. 23). It can be found growing as a terrestrial, understorey in shaded and moist forests, near river margins.

##### Phenology.

It was found in bloom and fruit in December, during the rainy season.

##### Etymology.

This species is named after Dr. Kate Hertweck, dear colleague and specialist in subtribe Tradescantiinae, in appreciation for her contributions to the systematics and evolution of Monocots and Commelinaceae, especially regarding the evolution of *Tradescantia*.

##### Conservation status.

*Tradescantia hertweckii* is known solely from the type collection and, following the IUCN recommendations (IUCN 2001), it should be considered Data Deficient (DD), until further collections and information becomes available.

##### Comments.

*Tradescantia hertweckii* was considered by me a doubtful specimen related to *T. fluminensis* and, for this reason, not included in my Master thesis in the initial account for T. subg. Austrotradescantia (at the time T. sect. Austrotradescantia; Pellegrini 2015). It was thought by me to putatively represent a natural hybrid between *T. fluminensis* and *T. umbraculifera*, but it differed greatly from the other putative hybrids. Added to that, the inflorescence morphology of *T. hertweckii* is very similar to the one of *T. fluminensis* and does not show the very peculiar inflorescence of *T. umbraculifera* (Fig. 5I). Finally, *T. umbraculifera* is not known to occur in the same locality as *T. hertweckii*, with the only other species in the subgenus known to occur in Paraty being *T. fluminensis*. For these reasons, I have decided to recognise *T. hertweckii* as a new species, instead of a natural hybrid.

**Figure 22.**
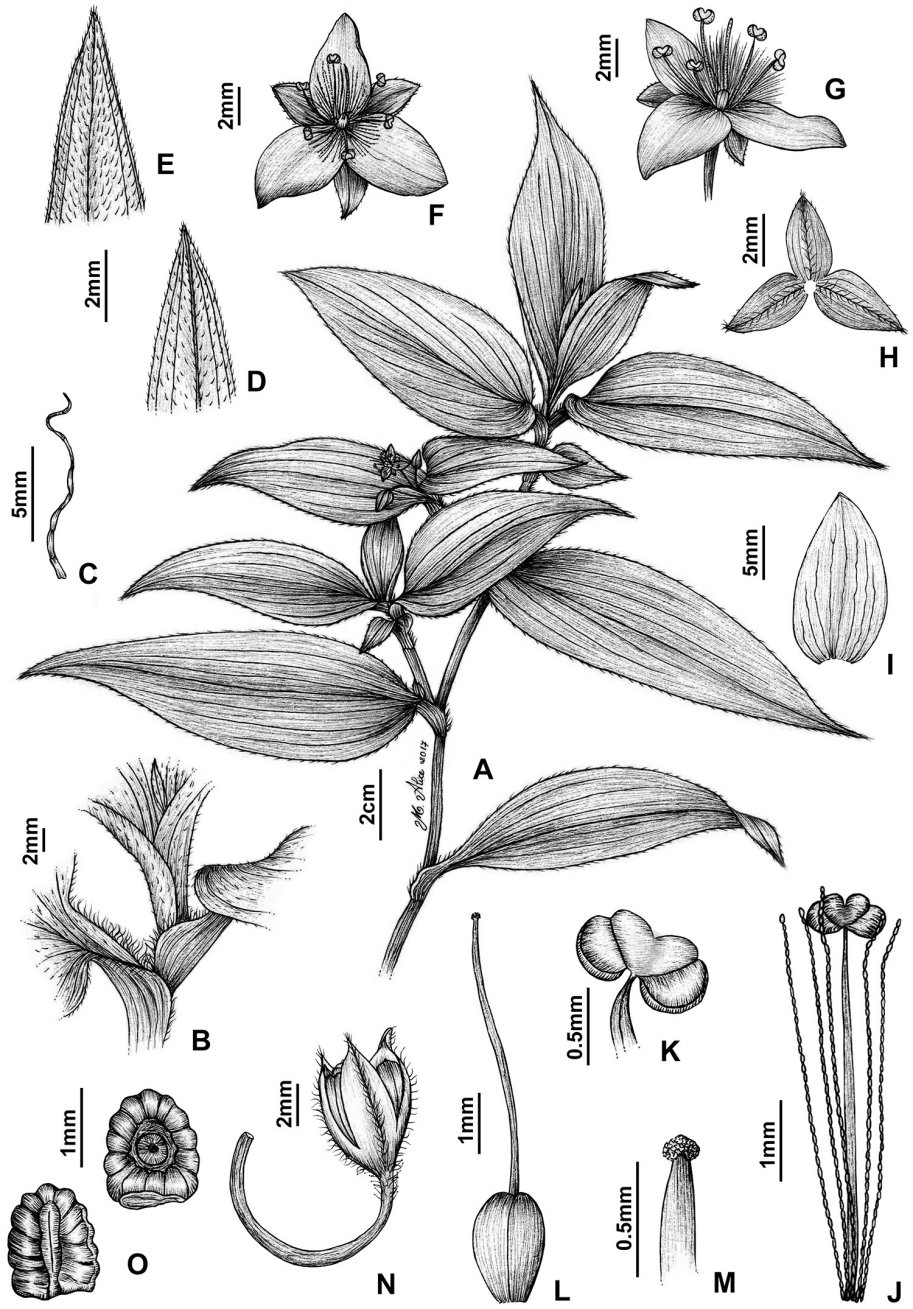
*Tradescantia hertweckii* M.Pell. **A** habit **B** detail of the leaf-sheaths, showing the setose margins **C** hair from the margin of the leaf-sheath **D–E** leaves **D** detail of the abaxial side of the apex of the blade, showing the hispid indumentum **E** detail of the adaxial side of the apex of the blade, showing the hispid indumentum **F–G** flowers **F** front view of a flower **G** oblique view of a flower **H** sepals, showing the setose indumentum restricted to the dorsal keels **I** petal **J–K** androecium **J** stamen **K** detail of the anther **L–M** gynoecium **L** pistil **M** detail of the stigma **N** open mature capsule **O** seeds, showing the costate testa not cleft towards the embryotega and the hilum longer than ½ the length of the seed. Line drawings by M.A. Rezende.

*Tradescantia hertweckii* is the only species from T. subg. Austrotradescantia not included by Pellegrini (2017) in his morphological phylogeny for the genus. However, it is a member of the *T. fluminensis* group, due to its indefinite base, stems prostrate with ascending apex, involute ptyxis, leaf-blades with conspicuous secondary veins (Fig. 22A), saccate cincinni bracts (Fig. 22A), pistil the same length as the stamens Fig. 22G) and seeds with costate testa not cleft towards the embryotega (Figs 7C, 22O). It is morphologically similar to *T. fluminensis* and *T. umbraculifera*. *Tradescantia hertweckii* is morphologically similar to *T. umbraculifera* due to its robust habit, sessile leaves, acuminate to caudate leaf-blades (Fig. 22A) and hilum longer than ½ the length of the seed (Figs 7C, 22O). Nonetheless, it can be easily differentiated from *T. umbraculifera* by its membranous to slightly fleshy leaf-blades covered by hispid indumentum (*vs.* chartaceous and glabrous or pilose in *T. umbraculifera*), 1 inflorescence per leaf axil (*vs.* 1–4), cincinni bracts leaf-like and unequal to strongly unequal (*vs.* spathaceous and equal), pedicels green at pre-anthesis and anthesis (*vs.* white) and pistil as long as the stamens (*vs.* longer than the stamens). On the other hand, *T. hertweckii* might be more easily confused with *T. fluminensis* *s.s.*, due to its glabrous stems (Fig. 22A), membranous to slightly fleshy leaf-blades, 1 inflorescence per leaf axil, leaf-like cincinni bracts (Fig. 22A), sepals with eglandular hairs restricted to the keels (Fig. 22H, N) and pistil as long as the stamens (Fig. 22 G). However, both species can be differentiated based on leaf morphology (leaves sessile, blades hispid, margins ciliate with densely setose base in *T. hertweckii vs.* subpetiolate, glabrous, evenly ciliolate in *T. fluminensis*), inflorescence morphology (cincinni bracts unequal to strongly unequal *vs.* equal), sepal pubescence (setose *vs.* pilose), petal posture (flat *vs.* plicate) and hilum relative length (longer than ½ the length of the seed *vs.* equal).

**Figure 23.**
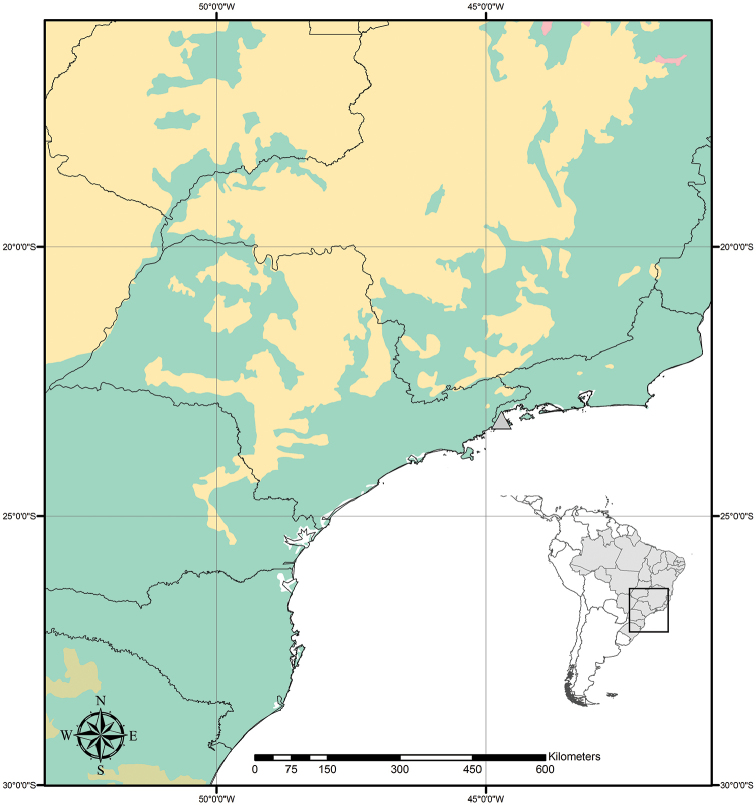
Distribution of *Tradescantia hertweckii* M.Pell. Orange– Cerrado; Olive-green– Pampa; Dark green– Atlantic Forest.

#### 9. Tradescantia mundula

Taxon classificationPlantaeORDOFAMILIA

Kunth, Enum. Pl. 4: 83. 1843.

Figs 24 , 25

Tradescantia mundula Kunth var. mundula . Holotype. URUGUAY. Montevideo: s.loc., fl., Dec 1836, F. Sellow 2103 (B barcode B100247279!).
Tradescantia fluminensis f. bicolor Voss in Siebert & Voss, Vilm. Blumengärtn. ed. 3, 1: 1143. 1895. Type. not found. **Syn. nov.**

##### Description.

*Herbs* ca. 5–30 cm tall, with an indefinite base, terrestrial, rupicolous or epiphytes. *Stems* prostrate with ascending apex, delicate to slightly succulent, little to densely branched; internodes 1–6.2(–9.3) cm long at base, distally shorter, medium to dark green or vinaceous, strigose, generally with a leaf-opposed longitudinal line of short, uniseriate, hyaline hairs. *Leaves* distichously-alternate, basal leaves subpetiolate; ptyxis involute; sheaths 0.5–1.2 cm long, green to vinaceous, strigose, margin sparsely setose to densely setose, hairs hyaline to light brown; petiole 0.2–0.5(–1–1.3) cm long to indistinct; blades 1.1–6.7(–7.8–13.4) × 0.6–3.4 cm, lanceolate to elliptic, rarely ovate, flat, chartaceous, adaxially glabrous to sparsely strigose to strigose, abaxially to sparsely strigose to strigose, rarely glabrous, adaxially medium to dark green, abaxially light to medium green or vinaceous, turning olive-green to medium brown when dry, base cuneate, rarely obtuse, margins green, ciliolate, slightly revolute, apex acute to acuminate; midvein conspicuous, adaxially impressed, secondary veins conspicuous, adaxially impressed, abaxially inconspicuous, becoming more evident on both sides when dry. *Synflorescences* terminal or axillar in the distal portion of the stems, composed of a solitary main florescence, 1 per leaf axis. *Inflorescences (main florescences)* consisting of a pedunculate double-cincinni fused back to back; peduncles 1.4–5.3(–9.3) cm long, medium to dark green or vinaceous, glabrous to sparsely strigose, with a dense longitudinal line of short, uniseriate, hyaline hairs; cincinni bracts 2.2–5.3(–8.7–10.6) × 1–2.1 cm, similar to each other, broadly elliptic to ovate, leaf-like, adaxially glabrous to sparsely strigose to strigose, abaxially sparsely strigose to strigose, adaxially green, abaxially light to medium green or vinaceous, base cordate to obtuse, saccate, margin ciliolate, slightly revolute, apex acute to acuminate; double-cincinni 6–8-flowered. *Flowers* 1–1.4 cm diam., pedicels 0.9–1.7 cm long, medium to dark green or vinaceous, velutine, sometimes with some odd glandular hairs, hairs hyaline; floral buds ovoid; sepals 5.1–7.5 × 2.4–4.1 mm, dorsally keeled, green, velutine, hairs generally caducous when dry, persistent only along the keel, hyaline; petals 6.3–7.6 × 3.8–5.1 mm, flat, white; filaments 4.4–5.6 mm long, anthers 1–1.2 × 0.8–1 mm; ovary 1.4–1.9 × 1–1.3 cm, style 3–5 mm long; pistil the same length as the stamens. *Capsules* 2.7–3.6 × 2.3–2.7 cm. *Seeds* 1–1.7 × 0.9–1.3 mm, testa greyish-brown to grey, not cleft towards the embryotega, costate; hilum equal ½ the length of the seed.

##### Specimens seen.

**ARGENTINA. Without province**: NE Argentina, fl., s.dat., s.leg. s.n. (K). **Buenos Aires**: Punta Lara, Selva marginal de La Plata, fl., 31 Oct 1948, A.B. Joly s.n. (IPA no. 69118, SPF no. 17142). **Chaco**: 1° de Mayo, Colonia Benítez, fl., 4 Oct 1973, A.G. Schulz 18472 (CORD, CTES); General Vedia, fl., fr., Oct 1939, A. Schinini 9329 (CTES); fl., 25 Aug 1974, A. Schinini 18801 (CORD, CTES). **Corrientes**: Santo Tomé, Arroyo Chimiray, fl., 23 Sep 1974, A. Krapovickas et al. 26247 (CORD, CTES); ruta 40 y Arroyo Chimiray, fl., fr., 8 Oct 1980, A. Schinini & O. Ahumada 20823 (CTES); Garruchos, Estancia San Juan Bautista, fl., 20 Sep 1974, Krapovickas et al. 25805 (CORD, CTES); Río Uruguay y Arroyo Chimiray, fl., 9 Oct 1980, A. Schinini & O. Ahumada 20931 (CTES). **Entre Rios**: Concordia, Parque Concordia, fl., s.dat., s.leg. s.n. (K); Salto Grande, fl., 21 Sep 1951, A.L. Cabrera 10773 (LP). **Misiones**: Apóstoles, San José, Bosque de Urunday, fl., fr., 3 Oct 1980, R. Martínez Crovetto 11473 (CTES); Concepción de la Sierra, fl., 3 Feb 1948, A. Schinini 6979a (CTES); Guaraní, Predio Guaraní, rumbo a Arroyo Paraíso, limite con IPS, fl., 16 Sep 1997, S.G. Tressens et al. 6037 (CORD, CTES); Iguazú, Puerto Peninsula, fl., 17 Feb 1971, A. Krapovickas et al. 18333a (CORD, CTES). **BRAZIL. Without province**: s.loc., fl., fr., s.dat., F. Sellow s.n. (B, P barcode P02173557). **Minas Gerais**: s.loc., fl., fr., 1816–1821, A. Saint-Hilaire B1 11 (P); Camanducaia, Monte Verde, fl., fr., 22 Aug 2001, L.D. Meireles et al. 511 (RB, UEC); fl., 20 Sep 2001, L.D. Meireles et al. 616 (RB, UEC); fl., fr., 19 Oct 2002, L.D. Meireles et al. 1191 (RB, UEC); trilha sentido Pedra Partida, fl., fr., 14 Oct 2014, D.Q. Domingos & B.C. Mourão 531 (ESAL, RB). **Paraná**: Antônia, Reserva Natural do Cachoeira, fl., 11 Sep 2009, B.D. Kellermann et al. 42 (FURB, JOI, UPCB); Araucária, Rio Iguaçú, fl., 13 Oct 1976, R. Kummrow 1160 (MBM, US); Balsa Nova, Serra S’Ana, fl., 11 Jan 1969, G Hatschbach 22790 (MBM, MO, US); Bocaiúva do Sul, Tunas, fl., 4 Oct 1977, G. Hatschbach 40210 (MBM, MG, US); Campo Largo, viaduto da Santa Rodovia de Café, fl., Sep 1971, L.T. Dombrowski 3829 (MBM, US); São Luiz do Purunã, fl., fr., 22 Sep 1976, L.T. Dombrowski 6424 (K, MBM); Colombo, Granja Esperança, fl., 9 Sep 1967, N. Imaguire 146 (K, MBM); Curitiba, Capanema, fl., 8 Jan 1948, G. Tessmann s.n. (MBM no. 265885, US barcode US00045774); estrada Curitiba-Ponta Grossa, km 38, Serra São Luiz de Purunã, fl., 18 Oct 1961, E. Pereira et al. 6093 (R, RB); Guabirotuba, fl., fr., 14 Dec 1978, G. Hatschbach 41895 (INPA, MBM, US); Parque Barigui, fl., 10 Oct 1973, L.T. Dombrowski 4930 (MBM, US); Parque Municipal Capão da Embuia, fl., fr., 25 Sep 2014, M.O.O. Pellegrini et al. 409 (RB); Uberaba, fl., 2 Oct 1983, G. Hatschbach 47158 (MBM, US); General Carneiro, Fazenda Lageado Grande, fl., 1 Oct 2004, A.C. Cervi 8680 (UPCB); Guarapuava, Serra da Esperança, fl., 20 Oct 1969, G. Hatschbach 22517 (CORD, NY, MBM, US); Jaguariaíva, Fazenda Chapada do Restingão, fl., 21 Oct 1999, M.K.F. Souza s.n. (UPCB no. 43238); Lapa, próximo à ponte junto ao Rio Passa Dois, fl., 4 Oct 2012, F. Santos-Silva et al. 149 (RB, UPCB); Londrina, distrito de Tamarana, Estância Dourados, fl., fr., 22 May 2008, V.M. Cotarelli 122 (FUEL); Sítio Casa das Pedras, fl., 20 Aug 1984, I.T. Takahara s.n. (FUEL no. 246); Marumby, Rio Taquaral, fl., 15 Nov 1943, G. Hatschbach 70 (RB); Serra do Mar, fl., fr., 11 Sep 1976, Y.S. Kuniyoshi 3154 (K, MBM); Mauá da Serra, Estância Manain, fl., 27 Aug 2008, V.M. Cotarelli & E.M. Francisco 251 (FUEL); Mata do Pinhão, fl., 1 Oct 2008, V.M. Cotarelli et al. 352A (FUEL); Morretes, Véu de Noiva, fl., 21 Aug 1985, J. Cordeiro & J.M. Silva 116 (HUEFS, MBM, RB, US); Palmeira, Córrego da Anta, fl., 30 Sep 1982, G. Hatschbach 45494 (INPA, MBM, US); Pato Branco, Loteamento Vila Campagna, fl., 7 Oct 2013, G. Felitto 867 (FURB); Ponta Grossa, fl., 17 Oct 1918, P. Dusén 10322 (K, S); fl., fr., 21 Sep 1971, L. Krieger 10815 (CESJ, K); Vila Velha, fl., Oct 1969, L. Krieger 7327 (CESJ, RB); fl., 25 Sep 1975, G. Hatschbach 38080 (MBM, US); próximo a Vila Velha, fl., fr., 11 Oct 1976, L.T. Dombrowski 6341 (K, MBM); Rio Branco do Sul, N of Rio Branco do Sul, fl., 28 Aug 1966, J.C. Lindeman & J.H. Haas 2385 (EFC, U, US); São Mateus do Sul, Fazenda do Durgo, fl., 31 Oct 1985, R.M. Britez 175 (UPCB); Telêmaco Borca, Fazenda Monte Alegre, à beira do Ribeirão Varanal, fl., fr., 9 Nov 2005, T.I.N. Azevedo et al. 232 (FUEL); fl., fr., 1 Nov 2006, T.I.N. Azevedo & S.I. Azevedo 534 (FUEL); Tibagí, Salto de Santa Rosa, fl., fr., 9 Oct 1994, A.C.S. Azevedo s.n. (FUEL no. 29802); fl., 27 Oct 1995, G.C. Barbosa et al. s.n. (FUEL no. 18422); Tijucas do Sul, Rincão, fl., 21 Oct 1977, G. Hatschbach 40482 (MBM, MG, US); União da Vitória, Rio do Tigre, fl., 27 Oct 1956, G. Hatschbach 3426 (MBM, US); Ventania, Fazenda Califórnia, fl., fr., 30 Aug 2005, D.A. Estevan et al. 978A (FUEL); Fazenda Santa Inês, fl., 17 Sep 2005, D.A. Estevan et al. 978B (FUEL). **Rio Grande do Sul**: s.loc., fl., 1833, C. Gaudichaud 269 (P); Augusto Pena, fl., 21 Aug 1953, P.J. Pivetta 962 (HRCB, PACA); Capão da Canoa, Estrada entre Riozinho e Maquiné, ca. 22 km E de Riozinho, fl., 23 Nov 2005, M.C. Machado & L.Y.S. Aona 594 (HUEFS, UEC); Caxias do Sul, estrada para Mulada, fl., fr., 10 Dec 2005, M.C. Machado & L.Y.S. Aona 740 (HUEFS, UEC); Esmeralda, Estação Ecológica Esmeralda, fl., Nov 1981, L.A. Cestaro 1 (HAS); Farroupilha, São José, fl., fr., 21 Oct 1984, R.A. Wasum et al. 480 (ALCB, HUCS, PACA); Palmeira das Missões, fl., 1957, K. Hagelund s.n. (CORD no. 467190, SI no. 26288a); Pelotas, Horto Botânico Irmão Teodoro Luiz, fl., Nov 1985, N. Mattos & M.H. Bassan s.n. (HAS no. 67814); Santana da Boa Vista, 60 km NE de Santana da Boa Vista, estrada para Passo do Marinheiro, fl., 10 Nov 2006, L.P. Queiroz & M.C. Machado 12344 (HUEFS); Santo Augusto, Estação Experimental Fitotécnica, fl., Oct 1981, J. Mattos & N. Mattos 22678 (HAS); fl., Nov 1983, J. Mattos et al. 25067 (HAS); São Francisco de Paula, Rincão dos Kroeff, 1 km da divisa da Barra do Ouro, fl., Nov 1985, J. Mattos & M.H. Bassan 29407 (HAS); São Lourenço do Sul, junto a ponte do Rio Camaquã, rodovia Pelotas-Porto Alegre, fl., Oct 1977, J. Mattos & N. Mattos 17375 (HAS); Torres, entre Torres e Dom Pedro de Alcântara, estrada para a colônia São Pedro, fl., Sep 1975, Z. Rosa s.n. (HAS no. 3124). **Santa Catarina**: s.loc., fl., fr., s.dat., D. D’Urville 112a (P) ;Campo Alegre, Pinheiral, lower slopes of Morro Iquererim, fl., 8 Nov 1956, L.B. Smith & R. Reitz 7374 (HBR, P, US); Campos Novos, fl., 14 Sep 2015, A.A. Oliveira 2836 (FURB); Catanduvas, E of Catanduvas, fl., 12 Oct 1964, L.B. Smith & R. Reitz 12436 (FLOR, HBR, K, P, R, US); Encanto, Indaial, fl., 21 Sep 1956, R. Reitz & R.M. Klein 3755 (HBR, MBM, NY, PACA, US); Grão Pará, Serra do Corvo Branco, fl., fr., 4 Oct 2014, M.O.O. Pellegrini et al. 434 (RB); Pano do Socorro, fl., Sep 1975, R.D. Cicilia s.n. (HAS no. 3154); Rancho Queimado, Serra da Boa Vista, fl., 14 Oct 1960, R. Reitz & R.M. Klein 10222 (HBR); Rodeio, bairro Ipiranga, fl., fr., 2 Oct 2014, M.O.O. Pellegrini et al. 427 (RB); São Bento do Sul, trilha do Parque Florestal do SAMAE, fl., 28 Sep 2014, M.O.O. Pellegrini & P. Schwirkowski 419 (RB); Rio Natal, beira da estrada sentido Rio Mandioca, curva próxima ao Restaurante Ruda, fl., fr., 28 Sep 2014, M.O.O. Pellegrini & P. Schwirkowski 423 (RB); São Joaquim, Fazenda Velha, fl., Jul 1956, J. Mattos 3703 (HAS); Urubici, Véu da Noiva, Avencal, ao longo do rio de pedras, fl., 5 Oct 2014, M.O.O. Pellegrini et al. 444 (RB). **São Paulo**: s.loc., fl., fr., 1833, C. Gaudichaud 158 (P); Atibaia, Itapetinga, fl., fr., 11 Nov 1987, A. Lofgren 347 (P, SPSF); Bonsucesso de Itararé, estrada Bonsucesso-Itararé, fl., 27 Nov 1993, V.C. Souza et al. 4789 (ESA, FUEL, RB); Pedreira Cobastalco, fl., 22 Nov 1994, K.D. Barreto et al. 3256 (ESA, RB, SPVR); descida da serra, fl., fr., 13 Nov 1994, V.C. Souza et al. 7226 (ESA, UEC, RB, SP, SPF); descida da serra entre a Pedreira Cobastalco e Bonsucesso de Itararé, fl., 19 Aug 1995, V.C. Souza et al. 8847 (ESA, HRCB, RB, SP, SPF, UEC, UFP); saindo da SP-258, 7 km de Bonsucesso, fl., 23 Oct 2007, R.C Forzza et al. 4774 (RB, SPF); Cunha, Parque Estadual da Serra do Mar, Núcleo Cunha, fl., 15 Nov 2006, E.J. Lucas et al. 301 (ESA, K, RB); Iporanga, Fazenda Intervales, base do Carmo, fl., 24 Aug 1992, M. Kirizawa et al. 2642 (RB, SP); Itapeva, Estação Experimental, trilha para o poço da Peroba, fl., 25 Aug 2009, C.R. Lima & R. Cielo Filho 243 (HURB); Jundiaí, fl., 23 Oct 2007, J.A. Lombardi 6981 (HRCB); Santa Izabel Igarata, fl., 26 Sep 1950, M. Kuhlmann 2548 (K, RB, SP); São Paulo, Butantã, fl., 27 Aug 1917, F.C. Hoehne s.n. (SP no. 463); Parque do Estado de São Paulo, fl., 28 Sep 1951, W. Hoehne 3604 (IPA, RB, SPF); Parque Morumbi, fl., 23 Oct 1987, V.C. Souza et al. 1087 (SPF). **URUGUAY. Montevideo**: s.loc., fl., s.dat., F. Sellow d1286 (B, K, P); Miguelete, fl., Nov 1925, W.G.H. Herter 436 (U).

##### Distribution and habitat.

*Tradescantia mundula* is known to occur in Argentina, Brazil (states of Minas Gerais, São Paulo, Paraná, Santa Catarina and Rio Grande do Sul) and Uruguay; in the Atlantic Forest, Chaco and Pampa domains (Fig. 25). It can be found growing understorey in shaded and moist forests and also in open forests, as a terrestrial, rupicolous or epiphyte.

##### Phenology.

It was found in bloom and fruit from July to February but peaking during the rainy season.

##### Etymology.

The epithet “*mundula*” means adorned, making reference to the strigose vegetative organs and the velutine sepals that characterise this species.

##### Conservation status.

*Tradescantia mundula* possesses a wide EOO (ca. 920,899.945 km^2^), being also a potentially aggressive weed worldwide. Thus, following the IUCN recommendations (IUCN 2001), it should be considered Least Concern (LC).

##### Nomenclatural notes.

As aforementioned, it is unknown where the specimens of any names described by A. Voss are housed (Stafleu and Cowan 1986) and, since Voss (in Siebert and Voss 1895) makes no reference to any kind of specimen, presents no illustration for most of his names and no specimens matching the diagnosis of T. fluminensis f. bicolor were located, I was unable to typify it.

##### Comments.

**Figure 24.**
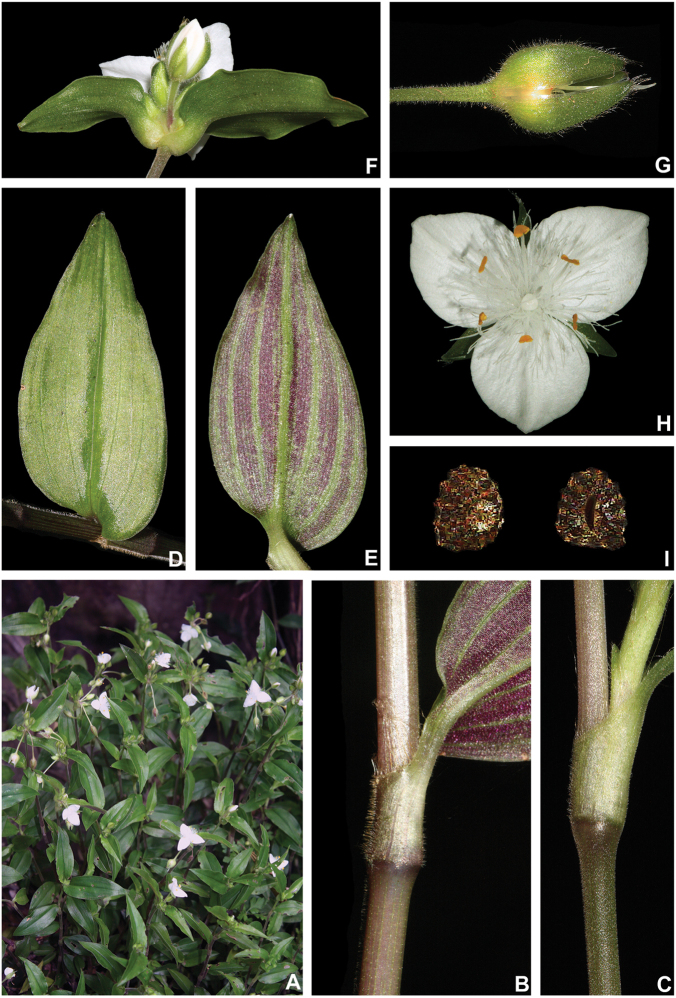
*Tradescantia mundula* Kunth. **A** habit, showing the mat-forming prostrate stems **B–C** stems and leaf-sheaths **B** detail of a sparsely strigose stem, leaf-sheath and subpetiole **C** detail of a strigose stem, leaf-sheath, subpetiole and young leaves **D–E** leaves **D** adaxial side of the blade, showing the medium green colouration and impressed secondary veins **E** abaxial side of the blade, showing the vinaceous tinted colouration **F** detail of the inflorescence, showing the saccate cincinni bract base **G** detail of flower at post-anthesis, showing the pedicel’s and sepals’ velutine indumentum **H** front view of a flower, showing the flat petals **I** dorsal and ventral views of the seed, showing the costate testa not cleft towards the embryotega and the hilum ca. ½ the length of the seed. Field photos by M.O.O. Pellegrini.

*Tradescantia mundula* is the smallest species from T. subg. Austrotradescantia, being comparable in size only with some specimens of *T. tenella*. It is morphologically similar to *T. cymbispatha* and *T. fluminensis* due to their indefinite base, prostrate stems with ascending apex (Fig. 24A), involute ptyxis, saccate cincinni bracts (Fig. 24F), white petals (Fig. 24F, H), pistil as long as the stamens (Fig. 24G, H), seeds with uncleft testa towards the embryotega and hilum ½ the length of the seed (Fig. 24I). It can be easily differentiated from *T. cymbispatha* by its subpetiolate leaves with impressed secondary veins (*vs.* sessile leaves with inconspicuous secondary veins, in *T. cymbispatha*), blades chartaceous and glabrous or unevenly covered by indumentum (*vs.* succulent and evenly covered by indumentum) and sepals dorsally keeled (*vs.* sepals without dorsal keels). It is more easily confused with *T. fluminensis*, especially in dried specimens, due to their leaves with impressed secondary veins, sepals dorsally keeled and gross floral morphology. *Tradescantia mundula* can be differentiated from *T. fluminensis* due to its strigose stems (*vs.* glabrous in *T. fluminensis*), leaves unevenly to evenly strigose (*vs.* glabrous), blades chartaceous and abaxially completely to partially vinaceous (*vs.* membranous to slightly succulent and abaxially light to medium green), sepals evenly velutine *in vivo* (*vs.* pilose with hairs restricted to the keels) and flat petals (*vs.* plicate).

**Figure 25.**
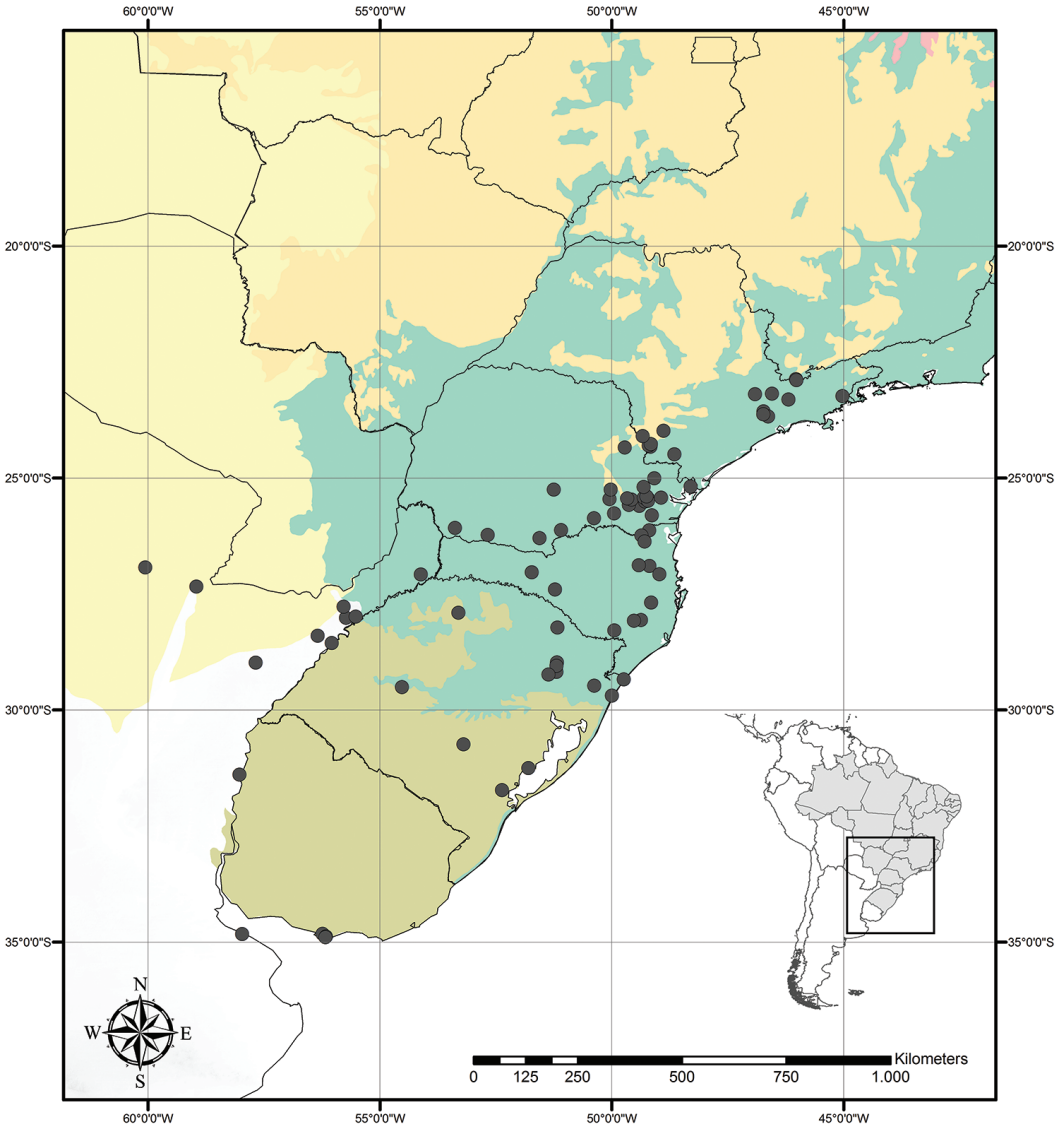
Distribution of *mundula* Kunth. Orange– Cerrado; Red– Caatinga; Yellow– Chaco and Pantanal; Olive-green– Pampa; Dark green– Atlantic Forest.

#### 10. Tradescantia seubertiana

Taxon classificationPlantaeORDOFAMILIA

M.Pell., Phytotaxa 265(1): 80. 2016.

Figs 26 , 27

##### Holotype.

BRAZIL. Santa Catarina: Grão Pará, Serra do Corvo Branco, 4 Oct 2014, fl., M.O.O. Pellegrini et al. 436 (RB barcode RB01045343!; isotypes: UEC!, US barcode US01926164!).

##### Description.

*Herbs* rupicolous, ca. 20–40 cm tall. *Stem* erect, succulent, little branched, branching at the base, rarely branching at the upper half; internodes 3.1–6.3 cm long at base, distally shorter, green to reddish-purple to vinaceous, glaucous, glabrous. *Leaves* distichously-alternate, sessile; ptyxis convolute; sheaths 0.4–1.3 cm long, green to green with vinaceous striations to vinaceous, glaucous, glabrous, margin glabrous; blades ovate to broadly ovate, 2.8–7.7 × 0.9–3.2 cm, falcate to complicate, succulent, glabrous, adaxially light-green, glaucous, abaxially slightly lighter to reddish-purple to vinaceous, glaucous, turning olive-green to light-brown when dry, base cordate to slightly amplexicaulous to obtuse, rarely cuneate, margin green, glabrous, slightly revolute, apex acute; midvein conspicuous to inconspicuous, adaxially impressed to inconspicuous, secondary veins inconspicuous on both sides, becoming more evident on both sides when dry. *Inflorescences* terminal or axillar in the distal portion of the stems, 1 per leaf axis; peduncles 1.6–3.7 cm long, green to reddish-purple to vinaceous, glaucous, glabrous; cincinni bracts unequal to strongly unequal to each other, ovate to broadly ovate, 0.7–3.3 × 0.4–1.2 cm, leaf-like, glabrous, adaxially light-green, glaucous, abaxially slightly lighter to reddish-purple to vinaceous, glaucous, base cordate to obtuse, not saccate, margin glabrous, slightly revolute, apex acute; double cincinni ca. 6–14-flowered. *Flowers* 0.8–1 cm diam., pedicels 0.7–1.4 cm long, green to reddish-purple to vinaceous, glaucous, glabrous, rarely with some odd glandular hairs; floral buds ellipsoid; sepals 4.8–5.9 × 2.4–4.6 mm, dorsally keeled, green to reddish-purple to vinaceous, glaucous, glabrous; petals 6.3–7 × 3.2–4.4 mm, light-pink to pink; filaments 2.8–3.2 mm long, anthers 0.5–0.8 × 1.3–1.5 mm; ovary 1–1.3 × 0.9–1.2 cm, style 4.3–8.5 cm long; pistil longer than the stamens. *Capsule* 1.8–2.6 × 1.8–2.2 cm. *Seeds* 1–1.2 × 0.8–1 mm, testa grey to greyish-brown, costate, cleft towards the embryotega; hilum longer than ½ the length of the seed.

##### Specimens seen.

**BRAZIL. Rio Grande do Sul**: Bagé, km 82 da rodovia Caçapava do Sul/Bagé, fl., fr., 30 Sep 1982, J. Mattos 25637 (HAS); Bento Gonçalves, Santa Teresa, fl., 20 Oct 1984, R.A. Wasum 511 (HUCS, PACA, US); na descida da Serra do Vale do Rio das Antas, fl., fr., 17 Oct 1988, N. Silveira & C.J. Mansan 6066 (HAS); Caxias do Sul, Galópolis, fl., 31 Oct 1949, A. Sehnem 4027 (CORD); Pelotas, Instituto Agronômico do Sul, fl., fr., Mar 1955, Boeira 1188 (HAS); Rio Pelotas, BR-116, km 270, fl., 23 Nov 1980, A. Krapovickas & R. Vanni 36870 (CORD, CTES); São Francisco de Paula, Barragem Passo do Inferno, fl., fr., 28 Nov 1996, C. Mansan & M. Neves s.n. (HAS 36560); fl., 27 Oct 1997, C. Mansan 65 (HAS); Santa Maria do Herval, fl., fr., 1 Nov 1989, N. Silveira 9272 (HAS); Vacaria, entre Vacaria e Caxias do Sul, fl., fr., 26 Oct 1961, E. Pereira et al. 6527 (R, RB); à 8km do Rio Pelotas, rodovia para Lajes, fl., fr., s.dat., J. Mattos & S.N. Mattos 24682 (HAS); fl., fr., 29 Nov 1986, J. Mattos & S.N. Mattos 30378 (HAS); BR-116, divisa com Lages, fl., 12 Dec 2015, M.O.O. Pellegrini & R.F. Almeida 481 (RB); Veranópolis, fl., 18 Oct 1986, G. Grazziotin 2149 (HPBR, HUCS, US); 10 km S de Veranópolis, fl., 16 Sep 1971, J.C. Lindeman & B. Irgang 8107 (CORD, CTES, EFC, HAS, ICN); Estação Experimental Fitotécnica, fl., fr., 4 Nov 1982, J. Mattos & S.M.H. Bassan 28557 (HAS); no vale do Rio das Antas, fl., 27 Sep 1985, J. Mattos & S.M.H. Bassan 28542 (HAS); no vale do Rio das Antas, fl., 27 Sep 1985, J. Mattos & S.N. Mattos s.n. (HAS no. 67967); no vale do Rio das Antas, na rodovia para Veranópolis, fl., fr., 27 Oct 1987, J. Mattos & S.N. Silveira 31228 (HAS). **Santa Catarina**: Urubici, Rio dos Bugres, fl., fr., 11 Nov 2001, G. Hatschbach et al. 72640 (MBM); Comunidade de São Pedro, fl., 15 Nov 2008, Silva et al. 7171 (MBM).

##### Distribution and habitat.

*Tradescantia seubertiana* is endemic to the alpine region of Rio Grande do Sul and Santa Catarina, Brazil; in the Atlantic Forest and Pampa domains (Fig. 27). It can be found growing on wet rock walls, from ca. 700 to 1.800 metres above sea level.

##### Phenology.

It was found in bloom and fruit from September to March.

##### Etymology.

This species was named after the prominent German botanist Moritz August Seubert (1818–1878), in appreciation for his extensive contribution to Commelinaceae systematics, especially for his contributions to the knowledge of the Brazilian Commelinaceae.

##### Conservation status.

As stated by Pellegrini (2015), *T. seubertiana* is known from few localities and very small populations, none of which are inside any conservation unit. This species is directly threatened by habitat loss, since all known collections are found along roads and high roads, currently being enlarged. In the near future, most known subpopulations might become extinct. Thus, following the IUCN recommendations (IUCN 2001), this species should be considered Critically Endangered [CR, A3cd+C2a(i)].

##### Comments.

**Figure 26.**
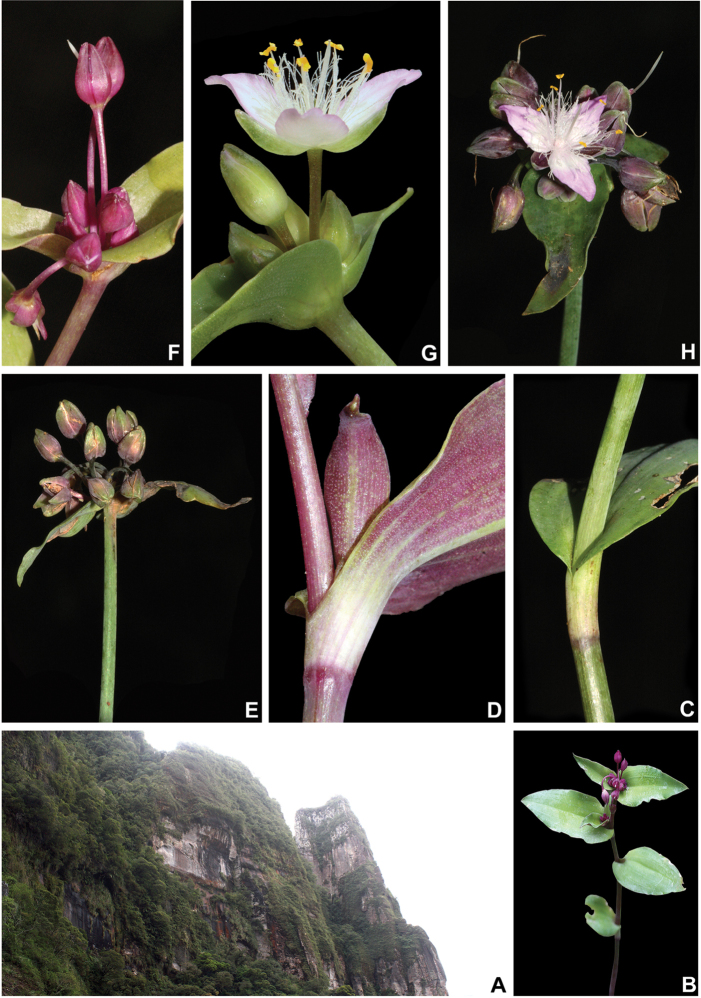
*Tradescantia seubertiana* M.Pell. **A** Serra do Corvo Branco, Santa Catarina, Brazil **B** habit, showing the erect stem **C** detail of the stem, leaf-sheath and leaf, showing the amplexicaulous leaf-blade base **D** detail of the stem, leaf-sheath and the abaxial side of the leaf-blade, showing the reddish-purple colouration and the complete absence of indumentum **E** detail of the inflorescence, showing the not saccate and unequal cincinni bracts **F** detail of the floral buds and flowers at post-anthesis, showing the reddish-purple colouration of the pedicels and sepals and the complete absence of indumentum **G** side view of a flower, showing the green sepals and pink petals **H** front view of a flower, showing the pistil longer than the stamens. Photos by M.O.O. Pellegrini.

*Tradescantia seubertiana* is a member of the well supported *T. crassula* group (Pellegrini 2017). This group is characterised by Pellegrini (2017) as comprising species with erect habit, definite base, convolute ptyxis, complicate to falcate and succulent leaves, generally with inconspicuous secondary veins (Fig. 26B–D), cincinni bracts non-saccate (Fig. 26E–G), pistil longer than the stamens (Fig. 26G–H), seeds cleft towards the embryotega, hilum longer than ½ the length of the seed and for preferentially inhabiting open areas and rocky outcrops. *Tradescantia seubertiana* is morphologically similar to *T. crassula* and *T. decora*, but only superficially similar to *T. cerinthoides*, especially due to its pink petals (which can range in *T. cerinthoides* from white to light pink to pink to lilac; Fig. 10N; Pellegrini 2015, 2017; Pellegrini et al. 2017). It can be differentiated from *T. crassula* and *T. decora* by its glabrous leaf-sheath margin – a very uncommon character in Commelinaceae – (*vs.* leaf-sheath margin ciliate in *T. crassula* and long ciliate in *T. decora*), leaf base cordate to slightly amplexicaulous to obtuse (*vs.* obtuse to truncate), ellipsoid flower buds (vs. broadly ovoid in *T. crassula* and *T. decora*), sepals glabrous (*vs.* setose along the keel in *T. crassula* and with minute caducous hairs at the apex of the sepals in *T. decora*) and by its light pink to pink petals (*vs.* white in *T. crassula*). It can be easily differentiated from *T. cerinthoides* by being almost entirely glabrous (*vs.* generally densely pubescent in *T. cerinthoides* with a mixture of glandular and eglandular, hyaline to light brown to golden hairs), inconspicuous secondary veins (*vs.* conspicuous in *T. cerinthoides*), its unequal cincinni bracts (*vs.* equal in *T. cerinthoides*), ellipsoid floral buds (*vs.* ovoid in *T. cerinthoides*) and keeled and glabrous sepals (*vs.* not keeled and densely pubescent in *T. cerinthoides* with a mixture of glandular and eglandular, hyaline to light brown to golden hairs). Additionally, *T. seubertiana* is found growing exclusively on wet rocky cliffs, while *T. cerinthoides*, *T. crassula* and *T. decora* are commonly found growing in open fields, sand dunes near the shore, dry rocky outcrops or understorey as terrestrial or epiphytes.

**Figure 27.**
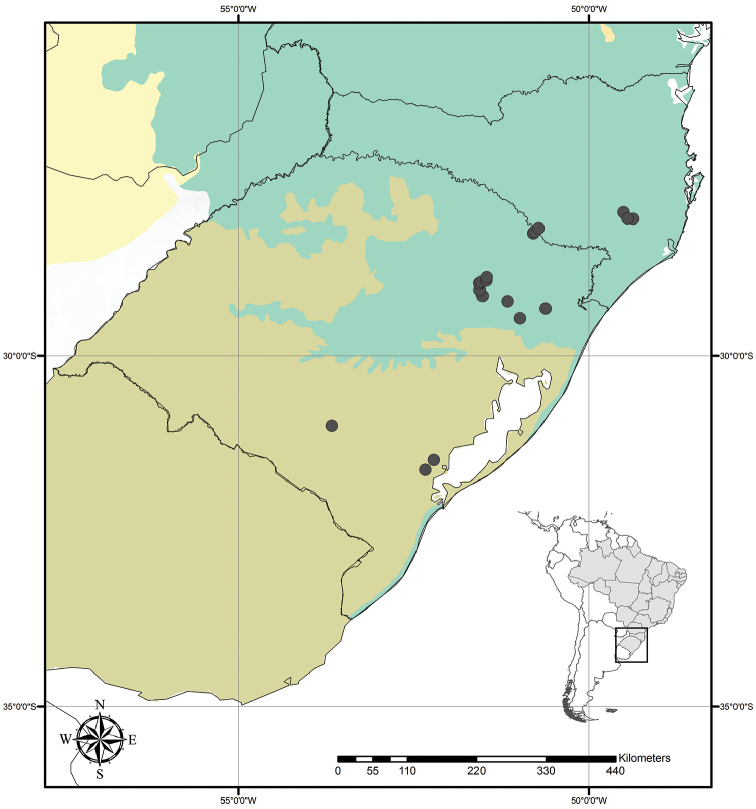
Distribution of *Tradescantia seubertiana* M.Pell. Yellow– Chaco and Pantanal; Olive-green– Pampa; Dark green– Atlantic Forest.

#### 11. Tradescantia tenella

Taxon classificationPlantaeORDOFAMILIA

Kunth, Enum. Pl. 4: 83. 1843.

Figs 28 , 29

Tradescantia fluminensis f. tenella (Kunth) Voss in Siebert and Voss, Vilm. Blumengärtn. ed. 3 1: 1143. 1895.
Tradescantia fluminensis var. tenella (Kunth) C.B.Clarke in De Candolle & De Candolle, Monogr. Phan. 3: 295. 1881. Holotype. URUGUAY. Montevideo, fl., fr., Dec 1836, F. Sellow d2290 (B barcode B100247280!).
Tradescantia anagallidea Seub. in Martius, Fl. bras. 3(1): 249. 1871. Lectotype (designated here). BRAZIL. s.loc., fl., fr., s.dat., F. Sellow 2801-67 (B barcode B100247281!; isolectotypes: IPA no. 54001!, MO barcode MO3020446!). **Syn. nov.**

##### Description.

*Herbs* ca. 5–50 cm tall, with a definite base, terrestrial, rupicolous or epiphytes. *Stems* erect, succulent, densely branched to fruticose; internodes 1.5–9.2 cm long at base, distally shorter, light to medium green to vinaceous, glabrous, sometimes glandular-pubescent or sparsely hirsute or a mixture of glandular and eglandular hairs, with a leaf-opposed longitudinal line of short, uniseriate, hyaline hairs in the terminal portion of the stems. *Leaves* distichously-alternate, sessile to subpetiolate; ptyxis involute; sheaths 0.3–1.3 cm long, light to medium green, glabrous to hirsute, margin densely setose, hairs hyaline to light brown; petiole 0.2–2.4 cm long to indistinct; blades (0.5–0.9–)1.2–14.8 × 0.5–3.6 cm, ovate to broadly ovate, rarely elliptic to lanceolate, flat, membranous to chartaceous, glabrous to sparsely hirsute on both sides, adaxially light to medium to dark green, sometimes with a vinaceous stripe along the midvein, abaxially light to medium green or vinaceous, turning olive-green to greyish-green when dry, base cordate to obtuse, rarely cuneate, margins green, ciliolate, slightly revolute, apex acute to acuminate; midvein conspicuous, adaxially impressed, secondary veins conspicuous, adaxially impressed, abaxially conspicuous, becoming more evident on both sides when dry. *Synflorescences* terminal or axillar in the distal portion of the stems, composed of a solitary main florescence, 1 per leaf axis. *Inflorescences (main florescences)* consisting of a pedunculate double-cincinni fused back to back; peduncles 1.9–5.3(–9.3) cm long, light to medium to dark green or vinaceous, glabrous, sometimes glandular-pubescent or sparsely hirsute, with a dense longitudinal line of short, uniseriate, hyaline to light brown hairs; cincinni bracts 0.4–8.1 × 0.2–3.1 cm, unequal to strongly unequal to each other, elliptic to ovate to broadly ovate, leaf-like, glabrous to sparsely hirsute, light to medium to dark green, sometimes with a vinaceous stripe along the midvein, abaxially light to medium green or vinaceous, base cordate to obtuse, saccate, margin ciliolate, slightly revolute, apex acute; double cincinni (4–)8–14-flowered. *Flowers* 0.4–1.0 cm diam., pedicels 0.4–1.7 cm long, green to vinaceous, sparsely glandular-pubescent to glandular-pubescent; floral buds ovoid; sepals 3–6.6 × 1.8–2.5 mm, dorsally keeled, green, sparsely glandular-pubescent to glandular-pubescent, rarely glabrous, hairs hyaline; petals 3.5–6.8 × 4–5.1 mm, white to white with pink apex to light pink to pink; filaments 3.8–4.1 mm long, anthers 0.9–1.1 × 1.2–1.8 mm; ovary 1.6–1.8 × 1.1–1.4 cm, style 3.5–3.8 mm long; pistil the same length as the stamens. *Capsules* 2.6–4.5 × 2.2–2.5 cm. *Seeds* 0.9–1.7 × 0.9–1.5 mm, testa grey to greyish-brown, not cleft towards the embryotega, rugose; hilum shorter than ½ the length of the seed.

##### Specimens seen.

**ARGENTINA. Buenos Aires**: Isla Martín García, fl., fr., 21 Oct 1992, J. Hurrell et al. 1244 (LP, MBM); Reserva Natural y Sitio Histórico Isla Martín García, fl., 30 Oct 2004, S. Torres Robles et al. 1987 (LP, US). **Corrientes**: s.loc., fl., fr., s.dat., A.J.A. Bonpland s.n. (P barcode P02173872); Capital, Corrientes, invernáculo del Instituto de Botánica del Nordeste, Facultad de Ciencias Agrarias, fl., fr., 10 Sep 2002, G. Seijo 2889 (CTES); Estancia, Santa Teresa, fr., 22 Oct 1949, T.M. Pedersen 440 (C, K, U, US); San Martín, Tres Cerros, Co. Capará, fl., fr., 15 Sep 1979, A. Schinini et al. 18456 (CORD, CTES); Sauce, 25 km SW de Sauce, ruta 126, fl., fr., 20 Oct 1977, O. Ahumada et al. 1174 (CTES). **Chaco**: Colonia Benítez, fl., 18 Sep 1942, A. Schinini 3539 (CORD, CTES). **Misiones**: Apostoles, San José, fl., fr., 14 Oct 1978, S.A. Renvoize 3090 (CORD, K, P, U, US, WAG); Cainguás, Salto del Tabay, fl., 12 Oct 1975, E.M. Zardini et al. 789b (LP, MO); Salto Golondrina, sobre Arroyo Guiray, fl., 8 Nov 2000, M.E. Múlgura et al. 2447 (CORD, SI); Candelaria, Santa Ana, fl., fr., 25 Nov 1945, J.E. Montes 1127 (K, LIL); Profundidad, fl., 19 Sep 1974, A. Krapovickas et al. 25712 (CORD, CTES); Iguazú, Puerto Istueta, fl., 27 Sep 1950, J.E. Montes 10201 (CORD, CTES, LIL); Parque Nacional do Iguazú, Sendero frente a Isla San Martín, fl., fr., 10 Aug 1995, R. Vanni et al. 4221 (CORD, CTES); Oberá, Salto Teodoro Cuenca, fl., 23 Sep 2000, M.E. Múlgura et al. 2282 (CORD, SI); Río Iguazú, fl., Sep 1900, G.E. Gerling s.n. (BA no. 16402); San Ignacio, San Ignacio, 3 a 5 km camino a Teyucuaré, fl., fr., 8 Nov 1913, M.N. Correa et al. 5371 (BAB, CTES, SI); San Pedro, Moconá, Prefectura Nacional Pepirí Mini, fl., 13 Oct 2003, D. Hojsgaard 307 (CTES, MNES). **Santa Fe**: Vera, campo de pruebas, FUNDAPAZ, fl., 11 Nov 2003, G. Marino 1906 (BA); próximo a La Gallareta, fl., 8 Jan 1997, J.F. Pensiero et al. 5215 (BA). **BRAZIL. Paraná**: Curitiba, Vila Nova, fl., fr., 21 Nov 1972, P. Occhioni 5238 (RFA); Foz do Iguaçú, fl., s.dat., J.G. Kuhlmann s.n. (RB no. 166655); fl., 7 Nov 1969, L.T. Dombrowski 2964 (MBM, US); Parque Nacional do Iguaçú, Cataratas do Iguaçú, fl., 27 Sep 1967, O. Boelcke 13449 (CORD, CTES); fl., 27 Sep 1967, A. Krapovickas 13373 (CTES); fl., 12 Aug 1969, G. Hatschbach 23166 (MBM, US); fl., 21 Aug 1985, G. Hatschbach & A.C. Cervi 49559 (MBM, UB, US); margem do Rio Iguaçú, abaixo das cataratas, próximo a administração do parque, fl., fr., 8 Oct 2012, R.C. Forzza et al. 7379 (RB, UPCB); Mangueirinha, Rio Iguaçú, Porto Fanor, fl., 20 Oct 1966, G. Hatschbach 15172 (MBM, NY, P, US, WAG); Morretes, Parque Estadual Pico do Marumbi, trilha de acesso ao Morro do Falcãozinho, fl., 19 Sep 1999, C. Kozera 1216 (UPCB); Guarapuava, Águas Santa Clara, fl., 1 Oct 1986, G. Hatschbach 50570 (MBM, US); Rio do Cedro, fl., 19 Oct 1967, G. Hatschbach 17490 (MBM, US); Morretes, Cadeado, fl., 14 Sep 1966, G. Hatschbach & A.O. Guimarães 14721 (MBM, US); Paranaguá, Porto Dom Pedro II, fl., fr., 26 Feb 1911, P. Dusén (NY, S); São José dos Pinhais, Serra dos Castelhanos, fl., 6 Nov 2004, A. Dunaiski Jr. 2595 (MBM); Serra do Mar, Desvio Ypiranga ad viam ferream, fl., fr., 23 Aug 1914, G. Jansson 885a (NY, S); Telêmaco Borba, Fazenda Monte Alegre, beira do Ribeirão Varanal, ponto 2, fl., 14 Oct 2004, T.I.N. Azevedo 6 (FUEL); fl., fr., 28 Aug 2006, T.I.N. Azevedo & S.I. Azevedo 400 (FUEL); fl., fr., 29 Aug 2006, T.I.N. Azevedo & S.I. Azevedo 433 (FUEL); margem da nascente do Ribeirão Varanal, fl., fr., 11 Nov 2005, T.I.N. Azevedo et al. 284 (FUEL); ponto médio do Ribeirão Varanal, fl., fr., 14 Oct 2004, T.I.N. Azevedo 7 (FUEL); fl., fr., 19 Jul 2005, T.I.N. Azevedo & J. Carneiro 151 (FUEL); fl., fr., 10 Nov 2005, T.I.N. Azevedo et al. 240 (FUEL); fl., 10 Nov 2015, T.I.N. Azevedo et al. 256 (FUEL). **Rio Grande do Sul**: Bom Jesus, Fazenda Bom Jardim, fl., 15 Nov 2003, R.A. Wasum & J. Bordin 2051 (HUCS, US); Caçapava do Sul, Guaritas, fl., fr., Sep 1985, M. Sobral & C. Grabauska 4291 (ICN, K); fl., fr., Sep 1985, M. Sobral & C. Grabauska 4295 (ICN, K); Camaquã, rodovia Dom Feliciano, fl., Nov 1978, J. Mattos et al. 19979 (HAS); Cambará do Sul, nos arredores de Ouro Verde, fl., Dec 1977, O.R. Camargo 5546 (HAS); Capivari do Sul, Águas Belas, fl., Oct 1989, V.F. Nunes & G. Rudh 495 (HAS); Charqueadas, Capão da Roça, fl., Sep 1986, J. Mattos & N. Mattos 30155 (HAS); Flores da Cunha, fl., 30 Oct 1999, L. Scur 102 (HUCS); Galópolis, Arroio Pinhal, fr., 29 Nov 1997, S. Diesel & E. Chavez 1039 (US); Guaíba, Fazenda São Maximiano, fl., 4 Oct 2009, N.I. Matzenbacher 3047 (ICN); Maquiné, Reserva Biológica da Serra Geral, Linha Solidão, trilha da casa do Sr. Lidorino, fl., Nov 2004, R. Schmidt 714 (HAS); Montenegro, Linha Campestre, fl., fr., 14 Nov 1948, A. Sehnem 3527 (PACA); fl., fr., 18 Oct 1950, A. Sehnem 4956 (CORD, FLOR, INPA, HBR, PACA, PEL, SI, UNOP); Polo Petroquímico, fr., Oct 1977, I. Ungaretti 758 (HAS); sub-região C, fl., Nov 1977, T. Buselato 113 (HAS); Osório, Terra de Areia, km 79, fl., Sep 1986, M. Bassan et al. 507 (HAS); Porto Alegre, Campo Novo, fl., fr., 14 Oct 2014, M.O.O. Pellegrini & F. Santos-Silva 449 (RB); Morro das Abertas, fl., fr., 30 Sep 1949, B. Rambo 43678 (HBR, K, PACA); Morro Santana, fl., fr., 5 Oct 1949, B. Rambo 43767 (HBR, PACA, US); São Francisco de Paula, RS-235, fl., 7 Nov 1999, R.A. Wasum 250 (HUCS, L, MBM); Reserva Biológica do Lami, fl., Sep 2003, A.S. Mello & D. Fuhro s.n. (HAS no. 45476); São Leopoldo, arredores de São Leopoldo, fl., Sep 1941, J. Eugênio 439 (NY); Torres, Arroio do Sal, fl., Sep 1985, N. Silveira et al. 3521 (HAS); Estiva, km 48 da estrada para Cidreira, fl., 22 Sep 1972, B. Irgang & L. Baptista 10224 (CORD, CTES, ICN); Itapuã, Granja Neugebauer, fl., fr., 11 Oct 1950, B. Rambo 48926 (HBR, PACA); fl., Oct 1984, N. Silveira et al. 2059 (HAS); fl., 8 Sep 1985, D.B. Falkenberg 3108 (FLOR, RB); Morro da Pedreira, fl., Oct 1979, O. Bueno 1847 (HAS); Vale do Sol, Morro do Espelho, Spielgerberg, fl., fr., 27 Oct 1992, J.L. Waechter 2529 (ICN, PEL). **Santa Catarina**: s.loc., fl., fr., s.dat., D. D’Urville s.n. (P barcode P02173875); Abelardo Luz, 4 km N of Abelardo Luz, fl., fr., 23 Oct 1964, L.B. Smith & R. Reitz 12878 (FLOR, HBR, P, R, US); Blumenau, Morro do Sapo, Parque Nacional Serra do Itajaí, fl., fr., 21 Jan 2013, L.A. Funez et al. 1610 (FURB); Bom Retiro, Fazenda Campo dos Padres, fl., fr., 17–19 Nov 1956, L.B. Smith & R. Klein 7726 (HBR, NY, P, R, RB, US); Fazenda Santo Antônio, Campo dos Padres, fl., fr., 23 Jan 1957, L.B. Smith & R. Reitz 10356 (HBR, P, US); Caçador, Pinheiral, fl., fr., 24 Nov 1956, L.B. Smith & R.M. Klein 7912 (HBR, P, R, NY, US); fl., fr., 21 Dec 1956, L.B. Smith & R. Reitz 8964 (HBR, R, US); Campo Novo, acesso alternativo ao Campo dos Padres, fl., fr., 12 Nov 2011, A.L. Gasper et al. 2916 (FURB, HURB); Cerro Negro, margem do Rio Tijolos, fl., fr., 22 Mar 2007, C.R. Grippa 33 (FURB); Grão Pará, Serra do Corvo Branco, fl., 4 Oct 2014, M.O.O. Pellegrini et al. 435 (RB); Itapema, São Paulinho, fl., 23 Sep 2009, A.S. Mello et al. 472 (FLOR); Itapiranga, near Rio Peperi-Guaçu, fl., fr., 17 Oct 1964, L.B. Smith & R. Reitz 12677 (HBR, NY, P, US); Joinville, estrada Dona Francisca, fl., fr., 4 Oct 1957, R. Reitz & R.M. Klein 4997 (HBR, US); Lauro Müller, Serra Rio do Rastro, fl., 14 Jan 1959, R. Reitz & R.M. Klein 8219 (HBR); Nova Trento, Rio Veado, fl., fr., 29 Sep 2010, A. Korte & A. Kniess 4496 (FURB, RB); Presidente Nereu, Braço Thieme, fl., fr., 15 Oct 2009, A. Korte & A. Kniess 433 (FURB, HURB); São Bento do Sul, Rio Natal, linha férrea, próximo ao CEPA Rugendas, fl., fr., 28 Sep 2014, M.O.O. Pellegrini & P. Schwirkowski 424 (RB); fl., fr., 30 Aug 2015, P. Schwirkowski 1204 (FURB); Serra Alta, Ponte do Rio Banhados, fl., P. Schwirkowski 197 (MBM); São Joaquim, fl., Oct 1961, J. Mattos s.n. (HAS no. 67802); Morro da Igreja, Campestre de Malacara, fl., Jan 1960, J. Mattos 7465 (HAS); Timbó, Parque Ecológico Freymund Germer, Morro Azul, fl., fr., 23 Aug 2012, L.A. Funez 775 (FURB, HUCS); fl., fr., 2 Oct 2014, M.O.O. Pellegrini et al. 431 (RB); Urubici, Avencal, Véu da Noiva, fl., fr., 15 Jan 2013, L.A. Funez & A.E. Zermiani 1400 (FURB, HUCS); fl., 5 Oct 2014, M.O.O. Pellegrini et al. 442 (RB); Morro da Igreja, fl., 4 Dec 1992, D.B. Falkenberg & F.A. Silva Filho 5859 (FLOR, RB). **PARAGUAY. Alto Paraná**: In regione fluminis, fl., 1909–1910, K. Fiebrig 5470 (SI). **Guairá**: Cordillera de Ybytyruzú, Ladero W del Cerro de Peró, 2 km E del Destacamento Tororo, fl., fr., 12 Nov 1988, E.M. Zardini 8010 (FCQ, K, MO). **URUGUAY. Lavalleja**: Cerro Arequita, fl., fr., 11 Oct 1970, H.A. Fabris & F.O. Zuloaga 7870 (LP); Penitente, fl., fr., 5 Nov 1933, W.G.H. Herter 1132a (U). **Montevideo**: s.loc., fl., fr., 1856, A. Courbon s.n. (P barcode P02173871); Bois de Santa Lucia, fl., 1 Nov 1969, M. Fruchard 930 (K, P); fl., fr., 1 Nov 1974, M. Fruchard s.n. (P barcodes P02174018, P02174023); fl., Mar 1976, M. Fruchard s.n. (P barcode P02174019, P02174022); Sierra de Animas, E of Montevideo, fl., 22 Nov 1981, L.R. Landrum, 3857 (US). **Río Negro**: Yapeyú, Paso Yapeyú, fl., fr., 15 Jun 1914, M.B. Berro 6061 (K). **Tacuarembó**: s.loc., fl., s.dat., W.G.F. Herter 1132 (BA, SI); Gruta de los Helechos, fl., fr., 28 Sep 1928, W.G.F. Herter 19 (SI).

##### Distribution and habitat.

*Tradescantia tenella* is known to occur in Argentina, Brazil (states of Paraná, Santa Catarina and Rio Grande do Sul), Paraguay and Uruguay; in the Atlantic Forest, Chaco and Pampa domains (Fig. 29). It can be found growing understorey in shaded and moist forests, between rocks near waterfalls and in clay ravines, as terrestrial, rupicolous or epiphyte.

##### Phenology.

It was found in bloom and fruit from August to January but peaking during October.

##### Etymology.

The epithet “*tenella*” means “delicate”, “small”, probably making reference to its delicate appearance and also to the small portion available to Kunth when describing the species.

##### Conservation status.

*Tradescantia tenella* possesses a wide EOO (ca. 791,707.235 km^2^), and generally forming dense subpopulations. Thus, following the IUCN recommendations (IUCN 2001), it should be considered Least Concern (LC).

##### Comments.

**Figure 28.**
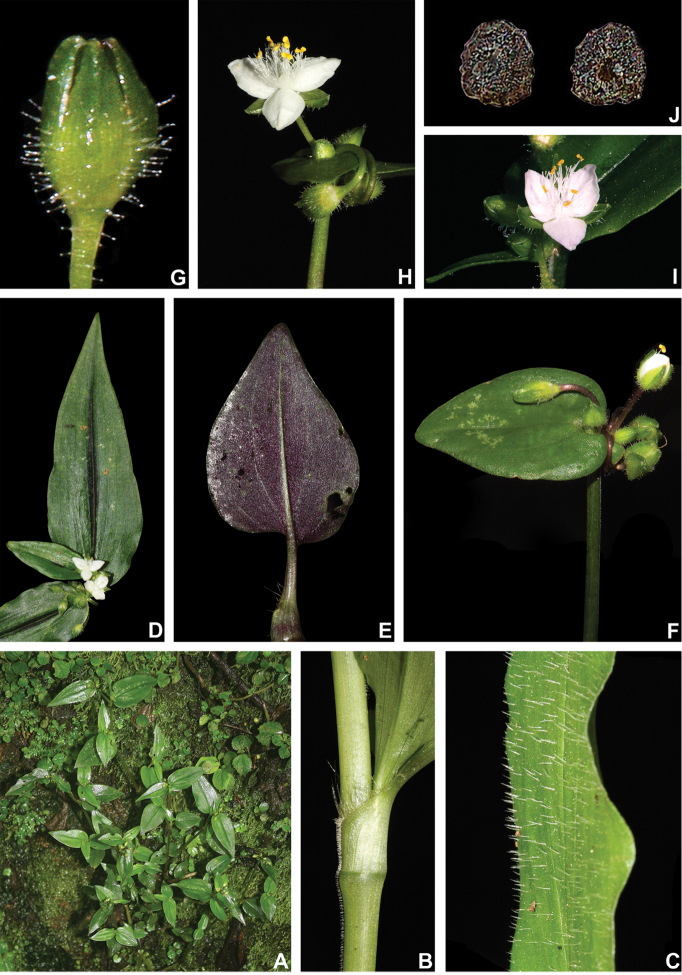
*Tradescantia tenella* Kunth. **A** habit, showing the definite base and fruticose and erect stems **B** detail of the stem and leaf-sheath, showing the leaf-opposed line of uniseriate hairs and the setose leaf-sheath margin **C** detail of the adaxial side of the leaf-blade, showing the hirsute indumentum **D** adaxial side of the leaf-blade, showing the impressed secondary veins and the vinaceous stripe along the midvein **E** abaxial side of the leaf-blade, showing the subpetiole and the vinaceous colouration **F** detail of the inflorescence, showing the saccate and strongly unequal cincinni bracts **G** flower at post-anthesis showing the glandular-pubescent indumentum and the dorsal keels of the sepals **H–I** flowers **H** white petalled flower **I** light pink petaled flower **J** dorsal and ventral views of the seed, showing the rugose testa not cleft towards the embryotega and the hilum shorter than ½ the length of the seed. Photos by M.O.O. Pellegrini.

*Tradescantia tenella* is one of the most peculiar species from the *T. fluminensis* clade. It possesses a definite base, erect and succulent stems (Fig. 28A) and flowers that range from white to pink (Fig. 28H, I), which are characters more commonly observed in species belonging to the *T. crassula* group. Nonetheless, it possesses involute ptyxis, subpetiolate leaves (Fig. 28E), saccate cincinni bracts (Fig. 28F, H, I), style as long as the stamens (Fig. 28H, I), seeds not cleft towards the embryotega and hilum shorter than ½ the length of the seed (Fig. 28J). These characters undoubtedly place *T. tenella* and related species (i.e. *T. atlantica* and *T. tucumanensis*) in the *T. fluminensis* group (Pellegrini 2017). Due to its peculiar morphology, *T. tenella* can be confused with species belonging to the *T. crassula* and *T. fluminensis* groups. *Tradescantia tenella* can be confused with *T. seubertiana* from the *T. crassula* group, due to their delicate habit, definite base, erect and succulent stems, unequal cincinni bracts and pink flowers. Nonetheless, it can be differentiated by its generally densely branched to fruticose stems (*vs.* little branched, branching at the base or rarely branching at the upper half in *T. seubertiana*), subpetiolate leaves with impressed secondary veins (*vs.* sessile with inconspicuous secondary veins), blades generally sparsely hirsute to hirsute with ciliolate margins (*vs.* always glabrous with glabrous margins), saccate cincinni bracts (*vs.* not saccate), pistil as long as the stamens (*vs.* longer than the stamens), seeds with testa rugose and not cleft towards the embryotega (*vs.* costate and cleft) and hilum shorter than ½ the length of the seed (*vs.* longer than ½ the length of the seed). In the *T. fluminensis* group, *T. tenella* can be confused with *T. mundula* due to their generally reduced stature, subpetiolate leaves and blades generally abaxially vinaceous. However, it can be easily differentiated due to its definite base (*vs.* indefinite in *T. mundula*), erect stems (*vs.* prostrate), leaf-blades generally sparsely hirsute to hirsute (*vs.* sparsely strigose to strigose), unequal cincinni bracts (*vs.* equal), sepals glandular pubescent (*vs.* velutine), petals ranging from white to pink (*vs.* always white), seeds with rugose testa (*vs.* costate) and hilum shorter than ½ the length of the seed (*vs.* equal to ½ the length of the seed). *Tradescantia tenella* is unquestionably morphologically closely related to *T. atlantica* and *T. tucumanensis*, forming the *T. tenella* complex. The species in this complex share the definite base, erect stems, chartaceous leaf-blades, flowers that range from white to pink, seeds with rugose testa and hilum always shorter than ½ the length of the seeds (Pellegrini 2017), being differentiated by stem consistency, the presence of a subpetiole, type and distribution of hairs in the sepals and petal morphology (see identification key).

**Figure 29.**
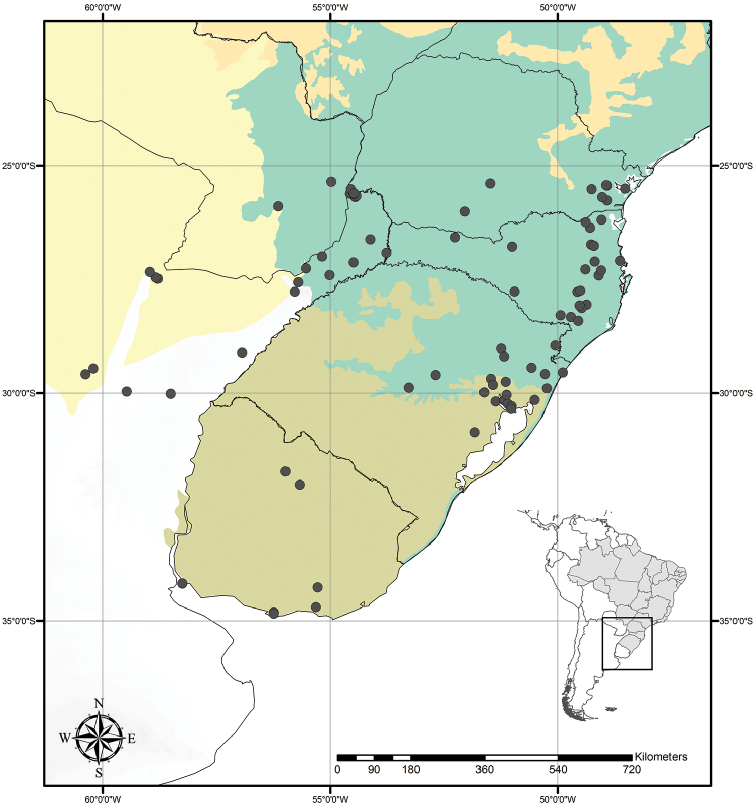
Distribution of *Tradescantia tenella* Kunth. Orange– Cerrado; Yellow– Chaco and Pantanal; Olive-green– Pampa; Dark green– Atlantic Forest.

#### 12. Tradescantia tucumanensis

Taxon classificationPlantaeORDOFAMILIA

M.Pell. sp. nov.

urn:lsid:ipni.org:names:77185917-1

Figs 30 , 31

##### Diagnosis.

Similar to *T. tenella* due to its definite base, densely branched stems, hirsute leaves, conspicuous secondary veins, saccate and strongly unequal cincinni bracts, keeled sepals, flat petals, pistil the same length as the stamens, seeds with rugose testa and hilum shorter than ½ the length of the seed. It can be differentiated by its prostrate stems with ascending apex, sessile to subpetiolate leaves, hyaline to light brown hairs, ellipsoid floral buds and sepals with a mixture of glandular and eglandular hairs, but exclusively hispid along the keel.

##### Type.

BOLIVIA. Santa Cruz: Florida, Tierras Nuevas, km 55 camino de Mairana a Postrevalle, fl., fr., 21 Nov 2004, J.R.I. Wood & H. Huayalla 21010 (holotype: K!; isotype: HSB!).

##### Description.

*Herbs* ca. 20–55 cm tall, with a definite base, terrestrial or rupicolous, rarely epiphytes. *Stems* erect, sometimes prostrate with ascending apex, succulent, little to densely branched; internodes 1.6–12.1 cm long at base, distally shorter, medium to dark green or vinaceous, sometimes with green longitudinal striations or spots, velutine to hispid, sometimes becoming glabrous with age, with a leaf-opposed longitudinal line of short, uniseriate, hyaline hairs in the terminal portion of the stems. *Leaves* distichously-alternate, sessile to subpetiolate; ptyxis involute; sheaths 0.4–1 cm long, medium green, with longitudinal vinaceous striations, sparsely velutine to hispid, margin setose, hairs hyaline to light brown; petiole 0.2–1.2 cm long to indistinct in the apical leaves; blades 1.1–8.7 × 0.9–3.2 cm, narrowly lanceolate to lanceolate or ovate to broadly ovate, flat, membranous to chartaceous, sparsely hispid to hispid on both sides, hairs hyaline to light brown, adaxially medium to dark green, abaxially light to medium green or vinaceous, turning medium brown to olive-green when dry, base cordate to round, margin green, ciliate, slightly revolute, apex acute to acuminate; midvein conspicuous, adaxially impressed, secondary veins conspicuous, adaxially impressed, abaxially inconspicuous, becoming evident when dry. *Synflorescences* terminal or axillar in the distal portion of the stems, composed of a solitary main florescence, 1 per leaf axis. *Inflorescences (main florescences)* consisting of a pedunculate double-cincinni fused back to back; peduncles (0.8–2.0–)3.4–8.2 cm long, velutine to hispid, with a leaf-opposed longitudinal line of short, uniseriate hairs, in the terminal portion of the stems, hairs hyaline to light brown; cincinni bracts 1.1–4.8 × (0.5–)1–2.7 cm, leaf-like, unequal to strongly unequal to each other, rarely similar to each other, elliptic or ovate to broadly ovate, sparsely hispid, hairs hyaline to light brown, adaxially medium to dark green, abaxially light to medium green or vinaceous, base cordate to obtuse, saccate, margins ciliolate to hispid, hairs hyaline to light brown, slightly revolute, apex acute to acuminate; double cincinni (4–)6–12-flowered. *Flowers* 1.2–1.6 cm diam., pedicels 0.7–2.2 cm long, vinaceous, with a mixture of glandular and eglandular hairs; floral buds ellipsoid; sepals 3.1–7.6 × 2.4–3.5 mm, keeled, green, with a mixture of glandular and eglandular hairs, hispid along the keel composed exclusively by eglandular hairs, hyaline to light brown; petals 6.6–7.2 × 3.3–5.2 mm, white to pink; filaments 5.6–6.3 mm long, anthers 0.6–0.7 × 0.8–0.9 mm; ovary 0.7–0.9 × 0.7–0.9 mm, style 5.4–6.5 mm long; pistil the same length as the stamens. *Capsules* 3.2–4.1 × 2.3–3.8 mm. *Seeds* 1.4–2.4 × 1–1.4 mm, testa grey, not cleft towards the embryotega, rugose; hilum shorter than or equal to ½ the length of the seed.

##### Specimens seen.

**(paratypes). ARGENTINA. Capital**: Villa Lujau, fl., 15 Nov 1922, S. Venturi 1981 (CORD, SI, US). **Catamarca**: Andalgalá, Esquena Grande, fl., 10 Nov 1916, P. Jörgensen 1817 (CORD, SI, US). **Chaco**: Colonia Benitez, fl., Dec 1935, A.G. Schulz 365 (CORD, SI); Laguna Blanca, fl., fr., 7 Dec 1939, M. Birabén 53 (LP). **Córdoba**: Villa General Belgrano, fl., 6 Sep 1985, M.E. Múlgura et al. 417 (CORD, MBM). **Corrientes**: Concepción, Carambola, fl., fr., 25 Sep 1971, T.M. Pedersen 9845 (L, SI); General Paz, G. Lomas de Vallejos, fl., 29 Aug 1973, A. Schinini & C. Quarín 6977 (CORD, CTES, SI); Ituzaingó, Ea. San Pedro, 27°45'S, 56°52'W, fl., fr., 12 Nov 1976, M.M. Arbo et al. 1172 (CORD, CTES); Mburucuyá, Estancia San Juan, fl., 22 Sep 1952, T.M. Pedersen 1835 (L); Mercedes, Ruta Nacional 14, laguna Iberá, 28°32'S, 57°10'W, fl., fr., 5 Nov 1973, M.N. Correa et al. 5306 (CORD, SI); Reserva Nat. Prov. Ibera, Paso Picada, costa W de la laguna Ibera, fl., fr., 11 Dec 1992, S.G. Tressens et al. 4342 (CTES, MBM, U); San Luis del Palmar, 10 km SE de San Luis del Palmar, Ruta 6, fl., 26 Sep 1973, C. Quarín & S.G. Tressens 1399 (CORD, CTES, SI); fl., 24 Oct 1976, A. Schinini & C.L. Cristóbal 13683 (SI). **Formosa**: Guaycolec, Monte, fl., Aug 1919, P. Jörgensen 3020 (BA, SI, US); Quebrada de Lules, fl., 21 Nov 1910, S. Venturi 1074 (BA, CORD, LP, SI, US). **Jujuy**: 13km del desvío de la Ruta Nacional 9, caminho a Tiraxi, fl., fr., 10 Aug 1998, O. Morrone et al. 3228 (CORD, SI); caminho a Tiraxi, 24°00'25"S, 65°22'19"W, fl., fr., 14 Nov 2005, F.O. Zuloaga et al. 8703 (CORD, SI); Ledesma, Abra de Cañas, caminho a Valle Grande, fl., fr., 31 Oct 1974, A.L. Cabrera et al. 25612 (LP); fl., fr., 7 Nov 1973, A.L. Cabrera et al. 23938 (CORD, LP, SI); fl., fr., 7 Nov 1973, A.L. Cabrera et al. 23945 (CORD, LP, SI); La Candelaria, El Trementinal, fl., Nov 1974, O. Marvin 19 (LP); caminho a Valle Grande, 24km NW de Libertador General San Martín, fl., 8 Nov 1974, A. Krapovickas et al. 26591 (CORD, CTES, SI); entre Mesada de las Colmenas y Abra Cañas, fl., 21 Oct 1979, A.L. Cabrera et al. 30908 (CORD, SI); caminho a Valle Grande desde Ruta Nacional 34, km 30, fl., fr., 16 Dec 1986, F.O. Zuloaga 2931 (CORD, SI); San Salvador de Jujuy, Zapla, fl., 9 Nov 1974, A.E. Burkart et al. 30419 (CORD, LP, SI); Sierra de Zapla, fl., 15 Nov 1980, A.L. Cabrera et al. 32027 (CORD, SI); fl., fr., 14 Nov 1992, R. Kiesling et al. 8263 (CORD, SI); Parque Nacional Calilegua, fl., 22 Nov 1986, O. Ahumada 5360 (CTES); fl., fr., 30 Sep 1994, O. Ahumada 7167 (CORD, CTES); monolito, 23°40'S, 64°54'W, fl., 3 Nov 1998, O. Ahumada & J. Agüero 8581 (CORD, SI); hasta el Arroyo Tres Cruces, por Ruta Provincial 83, 23°38'S, 64°56'W, fl., fr., 6 Nov 1998, O. Ahumada et al. 8764 (CORD, SI); Ruta Provincial 83, de a Abra de Cañas a Mesada de las Colmenas, 23°41'S, 64°54'W, fl., fr., 11 Nov 2002, F.O. Zuloaga et al. 7500 (CORD, SI); Santa Barbara, subida al Centinela, fl., fr., 12 Dec 1983, A. Rotman 939 (CORD, CTES, SI); Valle Grande, ruta 83, a 2km de San Francisco, fl., fr., 18 Jan 2002, G. Seijo et al 2755 (CORD, CESJ, CTES). **Salta**: Anta, Parque Nacional El Rey, fl., fr., 10 Nov 1974, T.M. Pedersen 10812 (CORD, L, SI); fl., fr., 14 Jan 1981, A. Brown et al. 1076 (CORD, SI); fl., Oct 2005, S. Chaluklan s.n. (SI barcode SI078836); Orán, Finca San Andrés, 23°05'53"S, 64°37'65"W, Sierra de Zenta, fl., 2 Nov 1997, A. Schinini et al. 33138 (CORD, CTES, SI); Salta, fl., 25 Oct 1985, C.A. Palací 221 (CORD); Santa Victoria, Ruta Provincial 19, 16km de Los Toldos, camino a Lipeo, fl., fr., 19 Nov 2001, O. Morrone et al. 3806 (CORD, SI). **Tucumán**: Chichigasta, Estancia Las Pavas, fl., fr., 9 Jul 1925, S. Venturi 4060 (CORD, SI, US); fl., 20 Nov 1926, S. Venturi 4705 (BA, CORD, SI, US); Entre Río Cochuna y Río Dulce, camino a Alpachiri, km 28.5, fl., 15 Oct 1966, N.M. Bacigalupo s.n. (CORD no. 467185, SI no. 26088). Famaillá, entre Famaillá y Santa Lucía, fl., fr., 4 Nov 2002, A.A. Cocucci 1982 (CORD). Ghieligasta, Puente del Rio Boehima, fl., 24 Nov 1938, D.C. O’Donell 10733 (US). Jafí, Jeoba Reserva, fl., 14 Nov 1923, S. Venturi 2386 (CORD, SI). Monteros, Rio de las Sosa, fl., 18 Oct 1948, L.B. Smith 4633 (US); km 33 La Helodera, fl., 10 Feb 1972, K. Jones et al. 10448 (CORD, SI). Rio Cochuna, fl., 24 Nov 1938, R. Schreiter 94754 (US); entre río Cochuna y río Dulce, camino a Alpachiri, fl., fr., 15 Oct 1966, N.M. Bacigalupo 26088 (SI). **BOLIVIA. Chuquisaca**: between Padilla and Monteagudo, between Leque Pampa and El Rosal, fl., 1 Jan 1995, J.R.I. Wood 9080 (K). **Santa Cruz**: M.M. Caballero, Comarapa, ca. 0.5–1 km pasando el Abra del Cerro Bravo, fl., fr., 20 Nov 2005, J.R.I. Wood & M. Mendoza 2209 (HSB, K). **Tarija**: Arce, valley of the Río Chillaguatas, below Rancho Nogalar on trail between Sidaras and Taroquia, 22°05'S, 64°25'W, fl., 14–16 Oct 1983, J.C. Solomon 11272 (K, MO, US); De Emboruzú a La Mamora, fl., fr., 22 Oct 1980, F.O. Zuloaga et al. 1208 (CORD, SI). **PARAGUAY. Without province**: s.loc., fl., May 1931, P. Jörgensen 4108 (SI, US). **Misiones**: Santiago, Estancia La Soledad, fl., 24 Oct 1959, T.M. Pedersen 5219 (L). **Guairá**: Iturbe, fl., fr., 8 Oct 1952, J.E. Montes 12776 (LIL, LP). **Ñeembucú**: Estancia Redondo, isla de bosque alto, fl., 25 Jan 2005, M. Vera et al. 230 (BM, FCQ).

##### Distribution and habitat.

*Tradescantia tucumanensis* is mainly distributed from Argentina to Bolivia, in the Tucumano-Boliviano Forest formation (Chaco and Andean Yungas domains), but also reaching Paraguay in the dry forests of the Atlantic Forest domain (Fig. 31). It can be found growing as a terrestrial or rupicolous herb and seems to be associated with considerably drier habitats than all other species from the *T. fluminensis* group.

##### Phenology.

It can be found in bloom and in fruit throughout the year but peaking during the rainy season.

##### Etymology.

The epithet makes reference to the species distribution, restricted to the Tucumano-Boliviano Forest formation in Argentina and Bolivia.

##### Conservation status.

*Tradescantia tucumanensis* possesses a wide EOO (ca. 873,174.442 km^2^), being widely distributed in Argentina and Paraguay, with few records in Bolivia. It seems to form dense subpopulations and, in accordance with the IUCN recommendations (IUCN 2001), it should be considered Least Concern (LC).

##### Comments.

**Figure 30.**
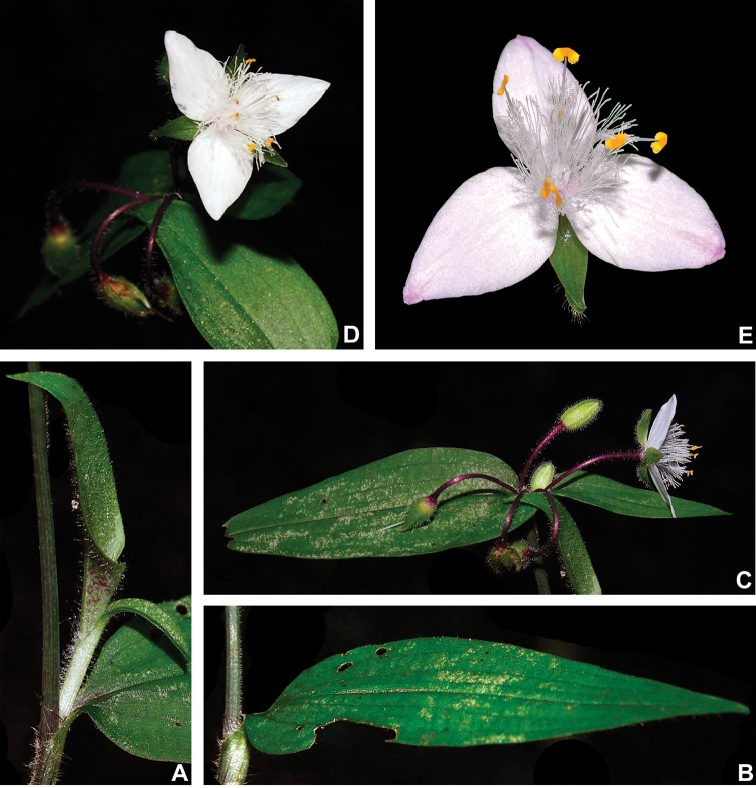
*Tradescantia tucumanensis* M.Pell **A** detail of the stem, leaf-sheath and young leaf **B** adaxial side of the leaf-blade, showing the impressed secondary veins **C** inflorescence, showing the unequal cincinni bracts, vinaceous pedicels, ellipsoid floral buds and the pubescence of the pedicels and sepals **D–E** flowers **D** white petalled flower **E** light pink petalled flower. **A–D** by G.A. Parada **E** by W.M. Ciesla.

*Tradescantia tucumanensis* was initially interpreted by me as a new species closely related to *T. chrysophylla*, due to its stems being sometimes prostrate with ascending apex, involute ptyxis, leaves velutine to hispid, indumentum drying light brown, unequal cincinni bracts (Fig. 30C), sepals with a mixture of glandular and eglandular hairs (Fig. 30C–E) and pistil as long as the stamens. Nonetheless, the similarities in gross morphology were quickly observed to be outnumbered by many more relevant differences: its definite base (*vs.* indefinite in *T. chrysophylla*), stems generally erect (*vs.* always prostrate), leaves generally subpetiolate (*vs.* always sessile), blades membranous to chartaceous with impressed secondary veins (*vs.* succulent with inconspicuous secondary veins), sepals keeled (*vs.* not keeled), hairs along the keel exclusively eglandular (*vs.* exclusively glandular or with a mixture of glandular and eglandular hairs), seeds with rugose testa (*vs.* costate) and hilum shorter than ½ the length of the seed (*vs.* equal ½ the length of the seed). Furthermore, *T. tucumanensis* is restricted to the Tucumano-Boliviano Forests from Argentina and Bolivia, while *T. chrysophylla* is endemic to the Atlantic Forest of Brazil. The seed morphology was key in making the connection between *T. tucumanensis* and *T. tenella*. Both species are morphologically more closely related due to their: definite base, erect stems (Fig. 30A), involute ptyxis, membranous to chartaceous leaf-blades (Fig. 30B), flowers ranging from white to pink (Fig. 30D, E), seeds with rugose testa and hilum shorter than ½ the length of the seeds. Nonetheless, both species can be differentiated based on the pubescence of the leaf-blades, shape of the floral buds, size of the flowers and pubescence of the sepals (see identification key). Out of these differences, floral bud shape and sepal pubescence have proven to be stable in reliable characters in the taxonomy of T. subg. Austrotradescantia. Furthermore, T. tucumanensis has a preference for drier environments than *T. tenella*, which is very commonly found growing in damp soil and moss carpets.

**Figure 31.**
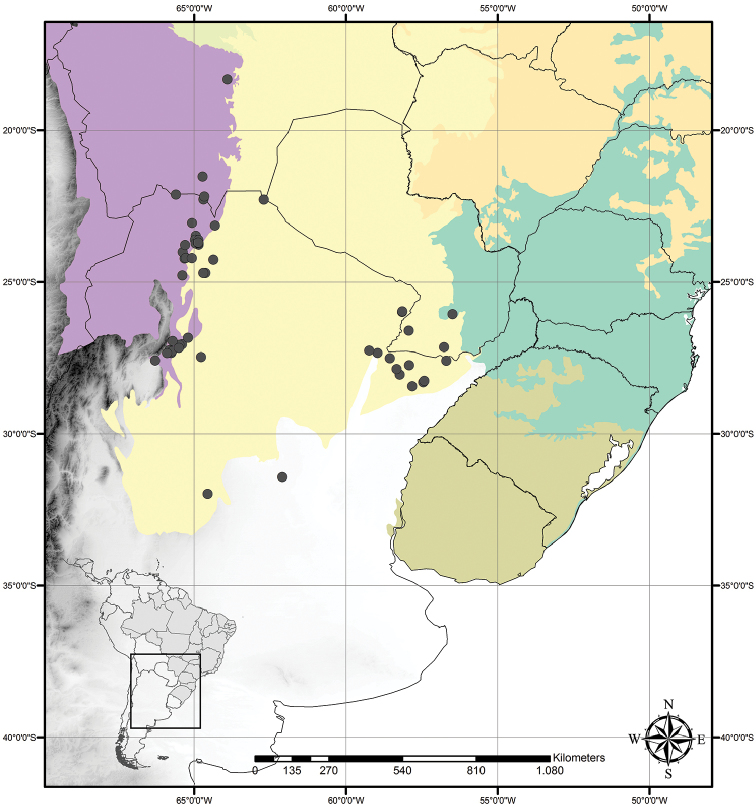
Distribution of *Tradescantia tucumanensis* M.Pell. Orange– Cerrado; Yellow– Chaco and Pantanal; Olive-green– Pampa; Dark green– Atlantic Forest; Purple– Andean Yungas.

#### 13. Tradescantia umbraculifera

Taxon classificationPlantaeORDOFAMILIA

Hand.-Mazz., Denkschr. Kaiserl. Akad. Wiss., Wien. Math.-Naturwiss. Kl. 79: 204. 1908.

Figs 32 , 33

Tradescantia fluminensis var. pubescens C.B.Clarke in De Candolle & De Candolle, Monogr. Phan. 3: 295. 1881. Lectotype (designated here). BRAZIL. Provincia de St. Paul and Rio, southern Brazil, fl., 1861–1862, J. Weir s.n. (K barcode K000363269!). **Syn. nov.**

##### Type material.

Lectotype (designated by Hassemer et al. 2017). BRAZIL. São Paulo: Campinas, fl., Jan 1900, J. Campos Novaes 1218 (WU barcode WU0061328!; isolectotype: US barcode US00046114!).

##### Description.

*Herbs* ca. 30–80 cm tall, with an indefinite base, terrestrial, rupicolous or epiphytes. *Stems* prostrate with ascending apex, succulent to slightly fibrous, little to densely branched; internodes 2.8–10 cm long at base, distally shorter, light to medium to dark green or reddish-purple to vinaceous, glabrous to sparsely pilose, with a leaf-opposed longitudinal line of short, uniseriate, hyaline to light brown hairs in the terminal portion of the stems. *Leaves* distichously-alternate, sessile; ptyxis involute; sheaths 0.5–2.3 cm long, light to medium to dark green or reddish-purple to vinaceous, glabrous to pilose to sparsely hispid, margin densely setose, hairs hyaline to light brown; blades 3.8–19.1 × 1–3.5 cm, linear lanceolate to lanceolate or narrowly lanceolate to lanceolate, flat, chartaceous, sometimes membranous, glabrous on both sides or adaxially glabrous and abaxially pilose, hairs hyaline to light brown, adaxially medium to dark green, abaxially light to medium green, rarely vinaceous, turning dark brown to black when dry, base truncate to amplexicaulous or round, margins green, ciliolate to ciliolate, flat, apex acuminate to caudate; midvein conspicuous, adaxially impressed, secondary veins conspicuous, adaxially slightly impressed to impressed, abaxially prominent, becoming more evident on both sides when dry. *Synflorescences* terminal or axillar in the distal portion of the stems, composed of a solitary main florescence, 1–4 per leaf axis. *Inflorescences (main florescences)* consisting of a pedunculate double-cincinni fused back to back; peduncles 0.5–4.3 cm long, light to medium to dark green, glabrous to pilose, with a dense longitudinal line of short, uniseriate, hyaline to light brown hairs; cincinni bracts 0.5–1.5(–2.3–6.2) × 0.2–1(–1.4) cm, similar to each other, cordate to broadly cordate, rarely lanceolate, spathaceous, rarely leaf-like, glabrous or pilose to sparsely hispid, medium to dark green, abaxially light to medium green, base cordate to obtuse, saccate, margins ciliolate to ciliolate, flat, apex acute, rarely acuminate; double cincinni (4–)8–12-flowered. *Flowers* 1.3–2.2 cm diam., pedicels 0.5–1.2 mm long, white to vinaceous, rarely green, glabrous to sparsely glandular-pubescent; floral buds ellipsoid; sepals 5.2–8.1 × 2–3.7 mm, dorsally keeled, green, glabrous or pilose, with hairs generally along the keel and at the base of the sepals close to the pedicel, hyaline to light brown; petals 0.7–1.2 × 0.3–0.6 cm, white; filaments 4.7–6.8 mm long, anthers 0.8–1 × 0.9–1.2 mm; ovary 1.2–2 × 0.8–1.3 mm, style 4.5–6.2 mm long; pistil longer than the stamens. *Capsules* 2.5–3.8 × 2.1–2.7 cm. *Seeds* 1.6–1.9 × 1.2–1.5 mm, testa grey to greyish-brown, not cleft towards the embryotega, costate; hilum longer than ½ the length of the seed.

##### Specimens see.

**ARGENTINA. Chaco**: 1° de Mayo, Colonia Benítez, fl., 10 Nov 1973, A.G. Schulz s.n. (CORD no. 469148, CTES no. 203094). **Corrientes**: Ituzaingó, Desembocadura del Arroyo Garapé em el Río Paraná, 45 km al E de Ituzaingó, fl., fr., 24 Apr 1975, A. Schinini et al. 11243 (CTES). **Misiones**: Apostoles, San Jose, fl., 11 Feb 1947, A.M.B. Huidobro 4981 (K, LIL); Cainguás, Salto Marvilla, fl., 4 May 1999, N.B. Deginani et al. 998 (CORD, CTES, SI); Candelaria, Cerro Azul, fl., fr., 16 Feb 1947, A.M.B. Huidobro 5282 (K, LIL); Loreto, fl., 12 May 1946, J.E. Montes 2223 (K, LIL); road from Bonpland to Oberá, fl., 23 Feb 1984, T.M. Pedersen 13668 (L); Picada Guemes, fl., fr., 21 Feb 1947, A.M.B. Huidobro 4859 (K, LIL); Profundidad, fl., 19 Sep 1974, A. Krapovickas et al. 25711 (CTES); Candelaria-Loreto, fl., fr., 22 Jul 1949, J.E. Montes 4222 (LIL, LP); Concepción de la Sierra, fl., 3 Feb 1948, A. Schinini 6979b (CORD, CTES); Eldorado, ruta 17, desvio 2 km a San Pedro, fl., 20 Jan 1973, A. Schinini & A. Fernandez 5933 (CORD, CTES); ruta 17, 89 km E de Eldorado, fl., 22 Jan 1973, A. Schinini & A. Fernandez 6002 (CORD, CTES); General Manuel Belgrano, 12 km S de Bernardo de Irigoyen, ruta 14, Cerro Tigre, fl., 15 Feb 1970, V. Maruñak 107 (CORD, CTES, LP); Guaraní, Predio Guaraní, picada al Arroyo Soberbio, fl., 15 Mar 1994, S. Tressens et al. 4881 (CORD, CTES); rumbo cerca de Papael Misionero, fl., 28 Apr 1999, S. Tressens et al. 6279 (CTES, K); Iguazú, Rio Paraná, 10 km S de Puerto Iguazú, frente a Puerto Bertoni, fl., 28 Mar 1970, A. Krapovickas et al. 15780 (CORD, CTES); Parque Nacional Iguazú, Sendero Macuco, fl., 6 Aug 1991, R. Vanni et al. 2666 (CORD, CTES); fl., 23 Apr 1996, J. Herrera 174 (CTES); Leandro M. Alem, 2 km al NE de Cerro Azul, fl., 10 Mar 1969, A. Krapovickas et al. 15070 (CORD, CTES); Libertador General San Martín, Predio UNLP, valle del arroyo Cuña Pirú, fl., fr., 19 Jul 1998, F. Biganzoli et al. 126 (LP, SI); Montecarlo, Arroyo Piray Guazú y ruta 12, fl., 15 Feb 1980, A. Schinini 19909 (CTES); San Ignacio, fl., 3 Feb 1947, J.E. Montes 822 (BA, LIL); Posadas, Jardín Botánico, fl., 17 Jul 2001, M. Grabiele 5 (CORD, CTES, MNES); San Pedro, 89 km E de Eldorado, fl., 22 Jan 1973, A. Schinini & A. Fernandez 6014 (CORD, CTES); ruta provincial 17, desvío 5 km a Tobuna por ruta provincial 224, fl., 26 Jan 1973, A. Schinini & A. Fernandez 6100 (CORD, CTES); entre San Pedro y Puerto Piray, 20 km de San Pedro, ruta provincial 16, fl., 28 Feb 1995, F.O. Zuloaga et al. 5063 (CORD, CTES, SI); cruzando el Puente sobre el Yabotí hacia los obrajes, ruta proyectada 102, fl., 9 May 1999, N.B. Deginani et al. 1213 (CORD, CTES, SI); Santa Anna, fl., 1907, s.leg. s.n. (LP no. 19175). **BRAZIL. Minas Gerais**: s.loc., fl., fr., 1816–1821, A. Saint-Hilaire D 548 (P); Aiuruoca, PCH-Aiuruoca, área de vazão reduzida, RPPN Cachoeira do Tombo, fl., 26 Mar 2009, P.H.A. Melo & D.M. Torres 3392 (ESAL, RB); Caldas, fl., 23 Mar 1868, A.F. Regnell 1445 (US); Cambuquira, Fazenda Esplanada, fl., fr., 25 Mar 2004, M.C. Weyland Vieira 2025 (RB); Carrancas, Serra de Bicas, fl., 30 Apr 1999, A.O. Simões et al. 821 (UEC); Lima Duarte, Parque Estadual da Serra do Ibitipoca, trilha entre a Gruta do Fugitivo e Gruta dos Três Arcos, fl., 11 Mar 2004, R.C. Forzza et al. 3173 (CEPEC, K, MBM, NY, RB, SPF, UEC); entre a Gruta dos Viajantes e a Gruta dos Três Arcos, fl., 20 Jan 2005, R.C. Forzza et al. 3965 (K, MBM, RB, SPF, UEC); Gruta dos Três Arcos, fl., 17 Mar 2005, R. Dias-Melo et al. 234 (CEPEC, NY, RB); Gruta dos Viajantes, córrego Monjolinho, fl., 25 Jan 2010, R. Mello-Silva 3239 (RB, SPF); São Thomé das Letras, próximo ao Pico do Gavião, fl., 22 Feb 1999, M.C. Assis et al. 596 (RB, SPF); APA São Thomé, Vale do Cantagalo, fl., 4 Feb 2015, E.R. Sattelmayer 22 (HUSC, RB). **Paraná**: Adrianópolis, Ponte do Ribeira, fl., fr., 20 Feb 1981, G. Hatschbach & A. Martínez 43629 (CORD, MBM, US); Rio Itapeva, fl., 19 Feb 1981, G. Hatschbach & A. Martínez 43622b (CORD, MBM, US); Antonina, Rio Cotia, fl., 24 Mar 1966, G. Hatschbach 14155 (MBM, P, RFA, US); Arapongas, Fazenda Solana, fl., 19 Feb 2009, M. Ferreira Jr. & E.M. Francisco 3 (FUEL); praça da igreja matriz, fl., 18 Jun 1988, L.V. Koga 10 (FUEL, HURB); Cerro Azul, cabeceira do Rio do Tigre, fl., 18 Jul 1984, G. Hatschbach 48100 (MBM, US); fl., 24 Apr 1987, G. Hatschbach et al. 51236 (MBM, US); Cornélio Procópio, Parque Estadual Mata São Francisco, fl., fr., 13 Mar 2009, G.M. Ferreira 358 (FUEL, UNIFIL); Foz do Iguaçú, Parque Nacional do Iguaçú, fl., 25 Apr 1949, J. Falcão 96 (RB); fl., 19 Feb 1960, E. Pereira 5364 (HB, RB); Londrina, Pirapó, fl., s.dat., G. Tessmann 1938 (FUEL, US); Londrina, estrada Paiquere, próximo à Fazenda Figueira, fl., 7 Feb 2003, D.A. Estevan et al. 1259 (FUEL); Fazenda Santa Helena, fl., fr., 5 Mar 2009, E.M. Francisco 2020 (FUEL); Parque Estadual Mata dos Godoy, fl., fr., 8 Mar 2010, E.F.S. Rossetto & E.M. Francisco 52 (FUEL); Mauá da Serra, Estância Manain, fl., fr., 1 Apr 2008, V.M. Cotarelli & E.M. Francisco 70 (FUEL); Rio Três Bocas, fl., fr., s.dat., R. Hertel s.n. (MBM no. 181892, US barcode US00046118); Rio Branco do Sul, Serra do Caete, fl., 10 Jan 1978, G. Hatschbach 40703 (MBM, US); fl., fr., 18 Feb 1981, G. Hatschbach & A. Martínez 43603 (CORD, MBM, US); Santo Antônio da Platina, Morro da Telepar, fl., 30 Jan 1974, R. Kummrow 516 (K, MBM); Sapopema, fl., 5 Apr 1996, L.P. Félix & A. Leforge 11 (RB, UEC); Telêmaco Borba, Fazenda Monte Alegre, estrada para a foz, fl., 20 Apr 2005, T.I.N. Azevedo et al. 81 (FUEL); estrada para a Ilha Surubim, fl., 20 Nov 1989, J.A. Pimenta et al. s.n. (FUEL no. 7628); à beira do Ribeirão Varanal, fl., 20 Apr 2005, T.I.N. Azevedo et al. 86 (FUEL); fl., 30 May 2006, T.I.N. Azevedo & S.I. Azevedo 324 (FUEL). **Rio de Janeiro**: Itatiaia, Maciço do Itatiaia, Parque Nacional do Itatiaia, estrada para o Pico das Agulhas Negras, fl., 15 Feb 1995, J.M.A. Braga et al. 2015 (RB); estrada região do planalto, fl., fr., 31 Jan 1966, S.V. Andrade 730 (RB); fl., 23 Jan 2012, M.O.O. Pellegrini 192 (RB); fr., 10 Jun 2012, M.L.O. Trovó et al. 569 (RB); fl., 2 Feb 2014, L.S.B. Calazans et al. 241 (RB); fl., 7 Feb 2015, R.G. Barbosa-Silva et al. 421 (RB); Agulhas Negras, fl., 17 Apr 1971, I.S. Gottsberger & G.K. Gottsberger 122-17471 (UB, US); Resende, Parque Nacional do Itatiaia, BR-485, Brejo da Lapa, fl., fr., 22 Feb 2014, L.S.B. Calazans et al. 241 (HRCB, RB, VIES); Rio de Janeiro, Realengo, fl., s.dat., C.V. Freire 397 (R); Teresópolis, Parque Nacional da Serra dos Órgãos, fl., fr., 16–17 Oct 1958, R. Schnell 8256 (BR, P); fl., 10 Jul 1966, A. Lourteig 1854 (K, P, R); fl., 3 Aug 1966, D.R. Hunt 6482 (K, US); fl., 5 Jul 1970, J. Barcia 82 (R); trilha para a Pedra do Sino, fl., 05 Dec 2001, F. Feres et al. 19 (RB, UEC); fl., fr., 12 Sep 2014, L.S.B. Calazans et al. 453 (RB); trilha do Rio Soberbo, fl., 22 Aug 2010, M. Nadruz et al. 2471 (RB). **Rio Grande do Sul**: Derrubadas, Tenente Portela, Parque Estadual do Turvo, Salto Yucunã, fl., 17 Mar 1977, K. Hagelund 11236 (CORD); fl., Mar 1980, J. Mattos et al. 21283 (HAS); fl., 1980, J.E.A. Mariath s.n. (CORD no. 467395, ICN no. 50979); Maquiné, Reserva Biológica da Serra Geral, Vale Linha Encantada, fl., Nov 2003, M.L. Abruzzi 5151 (HAS); São Leopoldo, Morro Sapucaia, fl., 5 Aug 1949, B. Rambo 42798 (HBR); fl., fr., 11 Oct 1955, B. Rambo 57048 (HBR). **Santa Catarina**: Blumenau, Morro do Sapo, Parque Nacional Serra do Itajaí, fl., 05 Nov 2012, L.A. Funez 1285 (FURB, HAVAT); Morro do Cachorro, fl., 31 Jan 2014, A.L. Gasper et al. 3439 (FUEL, FURB); Dona Emma, Gruta Nossa Senhora de Fátima, fl., 26 Jan 2012, L.A. Funez 309 (FURB); RPPN Bugerkopf, trilha principal, fl., fr., 6 Feb 2013, L.A. Funez 1681 (FURB); Florianópolis, trilha na margem sul da lagoa, Lagoa do Peri, fl., 3 Mar 2016, F.A. Silva Filho 963 (FLOR, RB); Ibirama, fl., 20 Oct 1953, R.M. Klein 614 (HBR, US); fl., 20 Sep 1956, R. Reitz & R.M. Klein 3692 (HBR, K, MBM, NY, UPCB); Itaiópolis, Arroio das Pombas, fl., 3 Feb 2010, A. Korte & A. Kniess 1631 (FURB); Itapiranga, Laranjeiras, SC-66, fl., 14 Feb 2009, M. Verdi et al. 1631 (FURB); Jaraguá do Sul, Serra do Jaraguá, fl., 31 Aug 1997, P. Schwacke 13350 (RB); Luiz Alves, Braço Joaquim, fl., 22 Mar 1956, R. Reitz & R.M. Klein 2886 (HBR, US); Mondaí, fl., 4 Feb 2009, M. Verdi et al. 2638 (FURB, RB); Linha Cascalho, fl., 5 Mar 2009, M. Verdi et al. 1746 (FURB, RB); Linha Capirara, BR-283, fl., 9 Mar 2009, M. Verdi et al. 77 (FURB, LUSC); Nova Teutônia, fl., 8 Apr 1944, F. Plaumann 444 (RB); Palmitos, 24 km E of Mondai, fl., 25 Feb 1957, L.B. Smith & R. Klein 11828 (HBR, NY, US); Paraíso, Rio das Flores, fl., 1 Mar 1964, A. Castellanos 24799 (CORD, K, RB); São Miguel D’Oeste, Peperi, fl., fr., 1 Mar 1964, R.M. Klein 5107 (HBR); Romelândia, Esperança, fl., 2 Mar 2009, A. Stival-Santos & S. Silveira 439 (FURB, RB); São Bento do Sul, Ano Bom/Braço Esquerdo, fl., 29 Jan 2010, S. Dreveck & F.E. Carneiro 1667 (FURB); Timbó, Castelo dos Padres, fl., 2 Oct 2014, M.O.O. Pellegrini et al. 429 (RB); Vidal Ramos, Sabiá, fl., 14 Jun 1957, R. Reitz & R.M. Klein 4284 (HBR, US); fl., 26 Nov 1957, R.M. Klein 2233 (HBR, NY, US). **São Paulo**: s.loc., fl., 1816–1821, A. Saint-Hilaire D 632 (P); Atibaia, Pedra Grande, fl., 29 Jan 1986, N. Taroda & K. Yamamoto 18324 (UEC); Barra do Turvo, 10 km de Barra do Turvo em direção a Pariquera-açú, fl., fr., 14 Feb 1995, J.P. Souza et al. 89 (ESA, SP, UEC, UFP); Cananéia, Parque Estadual da Jacupiranga, fl., fr., 24 Mar 2005, M. Carboni et al. 119 (ESA, UEC); Iepê, Fazenda C.A.P.I., about 5 km E of Porto Alvorada, along the rio Paranapanema, fl., 9 Feb 1965, G. Eiten et al. 5998 (EAC, INPA, MO, NY, SP, UB, US); Ilhabela, Parque Estadual da Ilhabela, trilha da Água Branca, fl., 23 Aug 1995, A. Rapini 48 (SP, SPF, UEC, UFP); São Paulo, Eldorado Paulista, Parque Estadual Jacupiranga, Núcleo Cedro, fl., fr., 14 Feb 1995, H.F. Leitão Filho et al. 33267 (HRCB, SP, SPF, UEC); Jardim Botânico e Parque do estado, fl., fr., 10 Aug 1967, R. Faria 40 (SP); Serra da Cantareira, fl., 13 Apr 1901, s.leg. 300 (SP); Serra da Cantareira, no Horto Florestal, fl., 30 Mar 1967, J. Mattos 14542 (HAS, K, RB, SP). **PARAGUAY. Without province**: s.loc., fl., s.dat., Fleischer s.n. (P barcode P02174039). **Alto Paraná**: s.loc., fl., 1909–1910, K. Fiebrig 6236 (K, L, US); Ciudaded del Este, Puerto Presidente Stroessner, fl., May 1983, L.C. Stutz 1773 (CORD, U, US); Puerto Embalse, fl., Mar 1942, L. Jiménez 122 (SCP); fl., Mar 1942, C.V. Pavetti Morin 488 (SCP); Vivero Florestal Itaipú, fl., 13 Jul 1980, G. Caballero Marmori 739 (CORD, CTES). **Canindeyú**: Jejui-mi, a unos 1500 m al N de la caseta, fl., 8 May 1996, G. Marín & B. Jiménez 191 (BM, CORD, CTES, FMB, PY). **Guairá**: Colonel Independencia, propriedad Vurtz, fl., 6 Oct 1967, A. Lourteig 1931 (P, US); Arroyo Guazú, camino a San Gervasio, fl., 27 Mar 1993, A. Schinini et al. 28031 (CORD, CTES); Reserva Ybytyruzú, fl., 27 Feb 2011, M. Vera et al. 2741 (FCQ); Tebicuary, Azucarera Paraguaya, fl., Jul 1942, C.V. Pavetti Morin 1186 (SCP); Villarica, Santa Barbara, fl., 26 Feb 1876, B. Balansa 590 (K, P). **Paraguarí**: Ybycui National Park, fl., 24 Jan 1989, E.M. Zardini & A. Aguayo 10090 (K, LP, MO, PY); fl., 15 May 1989, A. Aguayo 183 (CORD, US).

##### Distribution and habitat.

Argentina and Brazil (states of Minas Gerais, Rio de Janeiro, São Paulo, Paraná, Santa Catarina and Rio Grande do Sul) and Paraguay; in the Atlantic Forest, Cerrado and Chaco domains (Fig. 33). It can be found growing as terrestrial and epiphyte understorey in shaded and moist forests.

##### Phenology.

It was found in bloom and fruit throughout the year but peaking between August and February.

##### Etymology.

The epithet “*umbraculifera*” means “carrying several umbrellas”, making reference to the many inflorescences per leaf axil this species generally produces and, most importantly, to the its small spathaceous cincinni bract.

##### Conservation status.

*Tradescantia umbraculifera* possesses a wide EOO (ca. 764,678.067 km^2^), forming dense subpopulations in shady and moist understorey. Thus, in accordance with the IUCN recommendations (IUCN 2001), it should be considered Least Concern (LC).

##### Comments.

**Figure 32.**
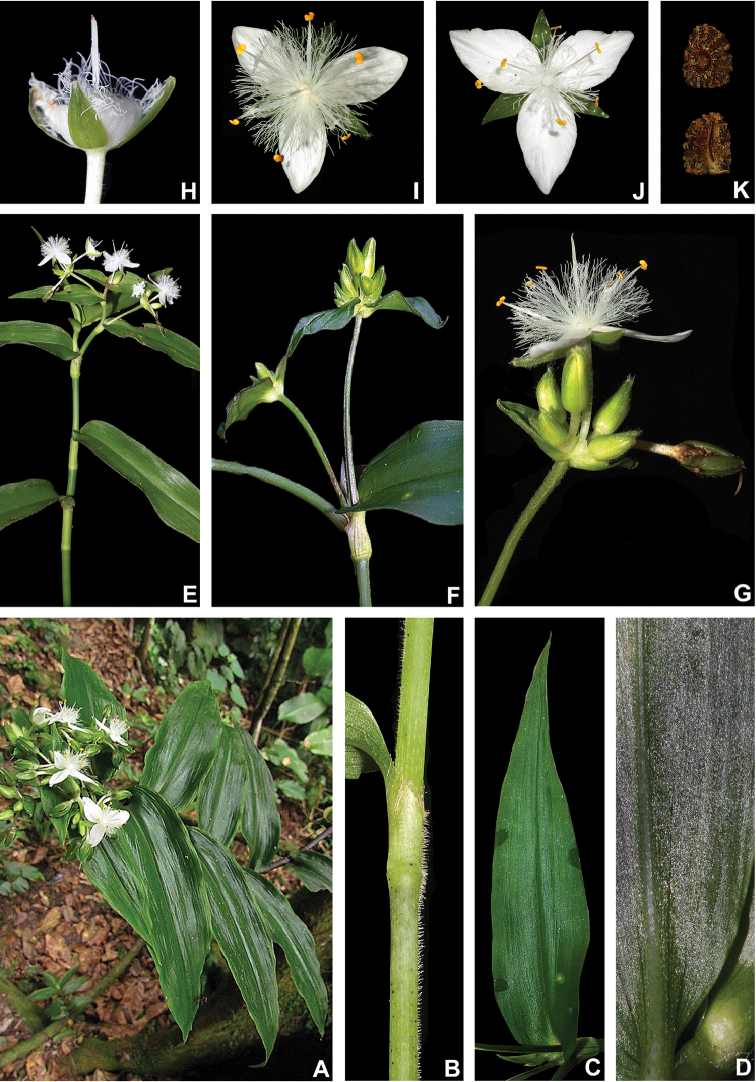
*Tradescantia umbraculifera* Hand.-Mazz. **A** habit, showing a specimen growing as an epiphyte **B** detail of the stem and the leaf-sheath, showing the leaf-opposed line of uniseriate hairs and the setose leaf-sheath margin **C** adaxial side of the leaf-blade, showing the impressed secondary veins **D** abaxial side of the leaf-blade **E** synflorescence **F** detail of the synflorescence, showing two inflorescences emerging from the same leaf axil and the spathaceous and saccate cincinni bracts **G** detail of the inflorescence, showing the spathaceous and saccate cincinni bracts and the side view of a flower, showing the pistil longer than the stamens **H** flower at post-anthesis showing the white pedicel and the glabrous sepals **I–J** flowers **I** front view of a flower of a typical specimen of *T. umbraculifera* **J** front view of a flower of a putative hybrid between *T. umbraculifera* and *T. fluminensis*, showing the leaf-like cincinni bracts, ovate petals, sparser filament hairs **K** dorsal and ventral views of the seed, showing the costate testa not cleft towards the embryotega and the hilum longer than ½ the length of the seed. Photos by M.O.O. Pellegrini, except **A** by L.A. Funez and **E, G–H, I** by H. Medeiros.

*Tradescantia umbraculifera* is a member of the *T. fluminensis* group (Pellegrini 2017), due to its indefinite base, prostrate stems (Fig. 32A), involute ptyxis, leaf-blades with impressed secondary veins (Fig. 32C), saccate cincinni bracts (Fig. 32E–G) and seeds not cleft towards the embryotega (Fig. 32K). Furthermore, dried specimens of *T. umbraculifera* acquire a peculiar dark brown to black colouration, which is recorded in T. subg. Austrotradescantia for *T. chrysophylla*, *T. cymbispatha* and some specimens of *T. fluminensis*. Nonetheless, it is one of the most peculiar species in T. subg. Austrotradescantia, due to its generally acuminate to caudate leaf-blades (Fig. 32C), numerous inflorescences per leaf axis (Fig. 32E, F), spathaceous cincinni bracts (Fig. 32E–G), white to vinaceous pedicels (Fig. 32H), pistil longer than the stamens (Fig. 32G) and hilum longer than ½ the length of the seed (Fig. 32K). This combination of characters differentiates *T. umbraculifera* from all remaining species of T. subg. Austrotradescantia. Smaller specimens of *T. umbraculifera* can be more easily confused with *T. fluminensis* but can be easily differentiated based on inflorescence and seed characters. Despite the distinction between *T. umbraculifera* and *T. fluminensis* being generally clean-cut, some specimens were especially challenging to certainly identify. This is mainly due to the presence of leaf-like, instead of spathaceous cincinni bracts (easily observable in herbarium specimens) and intermediate floral features between the two species (observable only in fresh specimens; Fig. 32J), such as: the colour of the pedicels, shape of the petals, length and density of the filaments hairs and pistil length. These specimens are hypothesised to represent naturally occurring hybrids between both species, since they were observed in areas where both *T. umbraculifera* and *T. fluminensis* were known to occur. However, due to the lack of reproductive, hybridisation and cytological studies, I have chosen to tentatively recognise these specimens under a broader sense of *T. umbraculifera*. Further studies might confirm my assumptions of the natural occurrence of hybrids between *T. umbraculifera* and *T. fluminensis*. Despite the small morphological resemblance, herbarium specimens of *T. umbraculifera* have been previously confused in Southern Brazil with *T. tenella*. Both species share only a handful of morphological characters, all of them being synapomorphies of the *T. fluminensis* group. *Tradescantia umbraculifera* can be easily differentiated from *T. tenella* due to its robust habit (*vs.* generally small in *T. tenella*), indefinite base (*vs.* definite), prostrate stems (*vs.* erect), sessile leaves (*vs.* subpetiolate), 1–4 main florescences per leaf axil (*vs.* always 1), cincinni bracts spathaceous and equal (*vs.* leaf-like and unequal) flowers 1.3–2.2 cm diam. (*vs.* 0.4–1 cm diam.), petals always white (*vs.* ranging from white to pink), pistil longer than the stamens (*vs.* equal), seeds with costate testa (*vs.* rugose) and hilum longer than ½ the length of the seed (*vs.* shorter than ½ the length of the seed).

Despite being in different morphological groups, herbarium specimens of *T. umbraculifera* have also been confused with *T. crassula*. This might be due these species robust habit, sessile leaves, sepals with hairs restricted to the dorsal keels, petals always white, pistil longer than the stamens and hilum longer than ½ the length of the seed. Nonetheless, *T. umbraculifera* can be easily differentiated by its indefinite base (*vs.* definite in *T. crassula*), prostrate stems (*vs.* erect), 1–4 main florescences per leaf axil (*vs.* always 1), cincinni bracts spathaceous and saccate (*vs.* leaf-like and non-saccate) and seeds not cleft towards the embryotega (*vs.* cleft).

**Figure 33.**
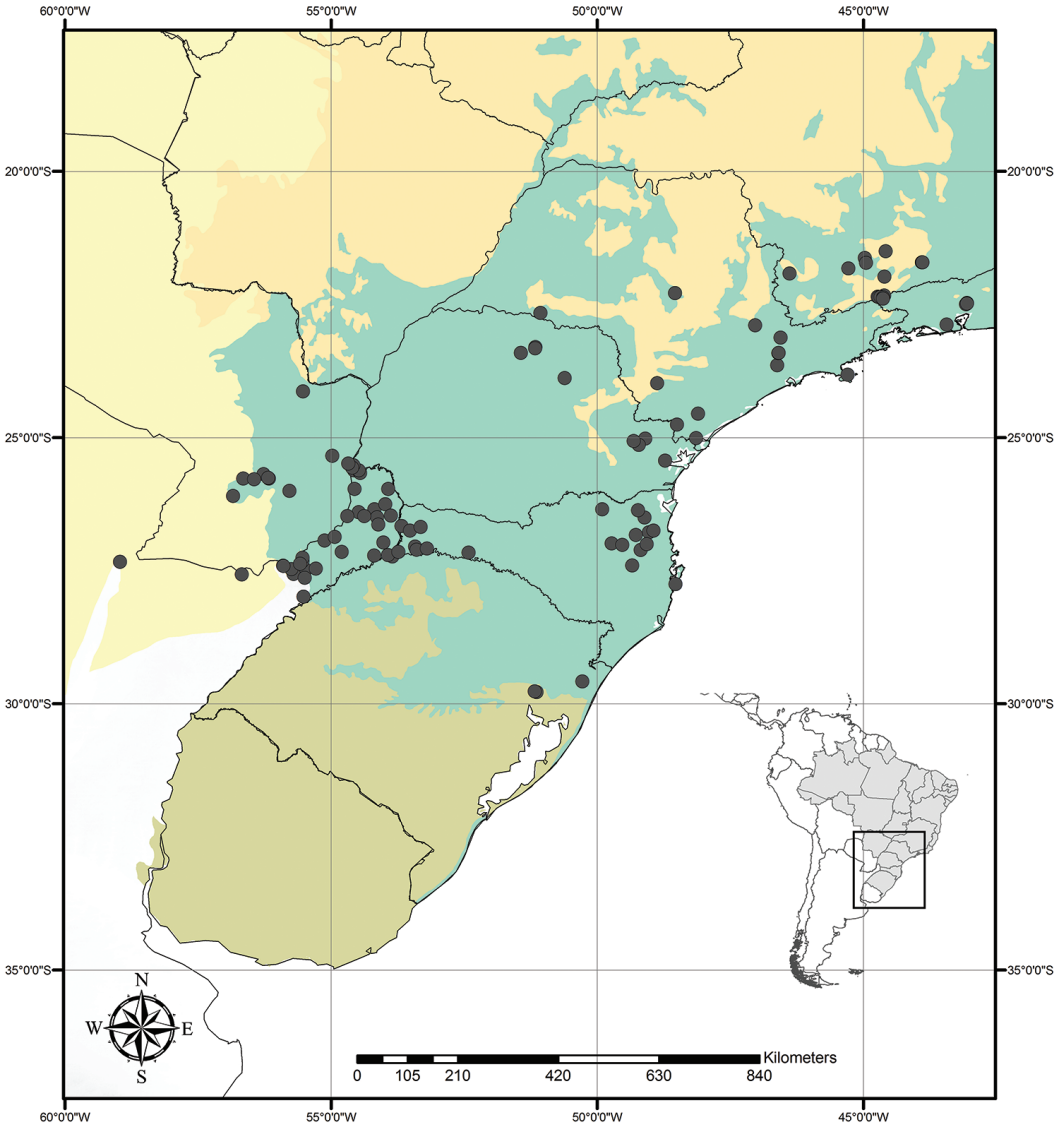
Distribution of *Tradescantia umbraculifera* Hand.-Mazz. Orange– Cerrado; Yellow– Chaco and Pantanal; Olive-green– Pampa; Dark green– Atlantic Forest.

## Final remarks

As stated by Pellegrini et al. (2017) and shown by Hunt (1975, 1980, 1986) and Pellegrini (2017), *Tradescantia* is a taxonomically complicated and morphologically diverse genus. Its morphology has hitherto been unsatisfactorily explored and many morphological characters historically used in its taxonomy are not completely reliable on their own. Further studies are still needed in *Tradescantia*, especially regarding the taxonomy within its subgenera. Tradescantia subg. Campelia, was considered by Pellegrini (2017) to be composed of ca. 15 species. However, ongoing studies in collaboration with Dr. David R. Hunt and Dr. Jason R. Grant, have revealed several undescribed species, and highlighted the need for more thorough studies in some species complexes (e.g. *T. commelinoides* and *T. zebrina*) and the need to revisit their taxonomy (Pellegrini et al. in prep.). Tradescantia subg. Mandonia is still poorly studied and understood and a taxonomic revision seems pressing. As exposed by Pellegrini (2017), T. subg. Tradescantia is still a taxonomically challenging group, with several poorly understood species, blurry specific boundaries and several putative natural hybrids. Nonetheless, the number of still undescribed species seems to be very low. Tradescantia subg. Setcreasea is taxonomically well-understood thanks to Hunt (1975, 1976), but lacks an updated identification key. Further studies in this subgenus should also address reproductive biology and population genetics studies.

This study presented the first complete taxonomic revision for one of the five subgenera of *Tradescantia*, proposed by Pellegrini (2017). I recognise 13 species, most of them widely distributed and presenting considerable morphological variation, as was also observed by Anderson and Woodson Jr. (1935) for the species of *Tradescantia*, native to the USA. The recognition of narrowly distributed and poorly-circumscribed taxa renders the taxonomy of *Tradescantia* unnecessarily complicated and most probably unnatural. The specific boundaries accepted by me for the species of T. subg. Austrotradescantia reflect extensive fieldwork throughout the Neotropical region, cultivation of almost all species of the subgenus and an understanding of the genus as a whole. My observations on dried and living specimens suggest that most variation in the genus might be ecologically related, with some changes being also putatively controlled by epigenetics and cytology. Unless focused studies on the reproductive and genetic features of these species are conducted, I strongly recommend that the broader species concepts herein proposed are followed.

## Supplementary Material

XML Treatment for Tradescantia subg. Austrotradescantia

XML Treatment for Tradescantia atlantica

XML Treatment for Tradescantia cerinthoides

XML Treatment for Tradescantia chrysophylla

XML Treatment for Tradescantia crassula

XML Treatment for Tradescantia cymbispatha

XML Treatment for Tradescantia decora

XML Treatment for Tradescantia fluminensis

XML Treatment for Tradescantia hertweckii

XML Treatment for Tradescantia mundula

XML Treatment for Tradescantia seubertiana

XML Treatment for Tradescantia tenella

XML Treatment for Tradescantia tucumanensis

XML Treatment for Tradescantia umbraculifera
